# Switchable foldamer ion channels with antibacterial activity†
†Electronic supplementary information (ESI) available: Synthetic procedures and compound characterisation. HPTS and PBC procedures and data. Antibacterial and haemolysis procedures. X-ray crystallography data. CCDC 1999499–1999501. For ESI and crystallographic data in CIF or other electronic format see DOI: 10.1039/d0sc02393k


**DOI:** 10.1039/d0sc02393k

**Published:** 2020-06-04

**Authors:** Anna D. Peters, Stefan Borsley, Flavio della Sala, Dominic F. Cairns-Gibson, Marios Leonidou, Jonathan Clayden, George F. S. Whitehead, Iñigo J. Vitórica-Yrezábal, Eriko Takano, John Burthem, Scott L. Cockroft, Simon J. Webb

**Affiliations:** a Department of Chemistry , University of Manchester , Oxford Road , Manchester M13 9PL , UK . Email: S.Webb@manchester.ac.uk; b Manchester Institute of Biotechnology , University of Manchester , 131 Princess St , Manchester M1 7DN , UK; c EaStCHEM School of Chemistry , University of Edinburgh , Joseph Black Building, David Brewster Road , Edinburgh EH9 3FJ , UK; d School of Chemistry , University of Bristol , Cantock's Close , Bristol BS8 1TS , UK; e Department of Haematology , Manchester Royal Infirmary , Manchester University NHS Foundation Trust , Manchester M13 9WL , UK; f Division of Cancer Sciences , School of Medical Sciences , University of Manchester , Manchester , UK

## Abstract

Synthetic ion channels may have applications in treating channelopathies and as new classes of antibiotics, particularly if ion flow through the channels can be controlled. Here we describe triazole-capped octameric α-aminoisobutyric acid (Aib) foldamers that “switch on” ion channel activity in phospholipid bilayers upon copper(ii) chloride addition; activity is “switched off” upon copper(ii) extraction. X-ray crystallography showed that CuCl_2_ complexation gave chloro-bridged foldamer dimers, with hydrogen bonds between dimers producing channels within the crystal structure. These interactions suggest a pathway for foldamer self-assembly into membrane ion channels. The copper(ii)-foldamer complexes showed antibacterial activity against *B. megaterium* strain DSM319 that was similar to the peptaibol antibiotic alamethicin, but with 90% lower hemolytic activity.

## Abstract 2

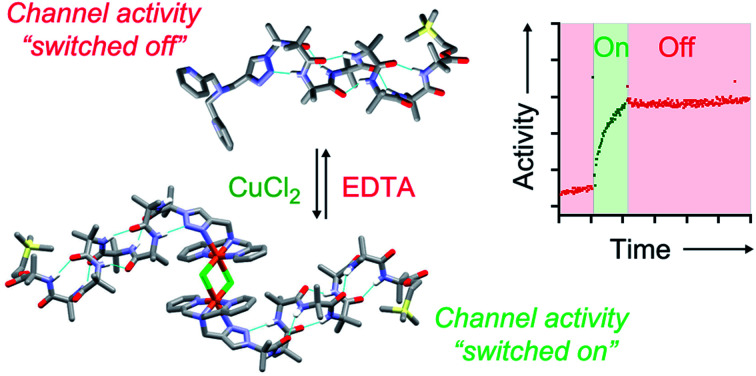
Triazole-capped α-aminoisobutyric acid (Aib) octameric foldamers formed very active ion channels in phospholipid bilayers after the addition of copper(ii) chloride, with activity “turned off” by copper(ii) extraction.

## Introduction

Natural ion channels carry out key functions in cellular membranes, including chemical and electrical signal transduction, control of cell volume, and maintenance of internal ion concentrations.^1^ Ion channel function is necessarily tightly regulated, as unregulated function can result in a disease state. Many diseases, such as epilepsy, myotonia, ataxia cardiac, arrhythmia, and cystic fibrosis, arise because of ion channel dysfunctions (channelopathies).^2^,^3^

Artificial ion channels are less structurally complex than natural protein ion channels,^4^ so their behaviour can be more easily analysed. Furthermore, synthetic ionophores could potentially alleviate the symptoms of channelopathies^5^,^6^ or provide new antibiotic classes able to overcome the growing problem of resistance.^7^ Of the wide range of artificial ion channels developed and studied,^8^ several show either cation selectivity^9^ or anion selectivity.^10^ However, a significant challenge in the field is to “switch” any channel activity,^11^ so that, like natural examples, activity can be controlled by external stimuli, such as light or chemical messengers. Closing channels by blocking the lumen with an added ligand is known, but examples of ligand-induced channel opening are less common.^12^ These have typically involved ligand-mediated self-assembly of channels in a membrane; for example, dialkoxynaphthalene addition to rigid-rod *p*-octiphenyl staves gave weakly anion selective channels.^13^ Metal ions, such as palladium(ii), have also been used to assemble channels within membranes.10a,^14^,^15^,^16^ Alternatively, multivalent ligands outside the membrane can drive the lateral assembly of individual membrane-spanning staves. This was elegantly shown by Matile and co-workers, who used coordination of external polyhistidine to copper(ii)(iminodiacetate)-terminated *p*-septiphenyls to open K^+^-selective channels.^17^

α-Aminoisobutyric acid (Aib) foldamers have recently shown promise for synthetic ion channel^18^ and signal transduction activities.^19^ These highly hydrophobic foldamers contain high proportions of Aib, a residue that favours folding into 3_10_ helices.^20^ Simple Aib foldamers in phospholipid bilayers show length-dependent ion channel and antibiotic activity,^18^ with properties that mimic the ionophoric behaviour of the Aib-rich antimicrobial peptide alamethicin, the archetypical peptaibol.^21^ These easy-to-modify Aib foldamers make an interesting platform for developing switchable ionophores that may also show antibacterial activity. For example, Cu(ii)-capped Aib foldamers might cluster upon binding to an external polyvalent ligand (*e.g.* polyhistidine),^22^ perhaps “switching on” ion channel activity that is reversible by ligand sequestration. Alternatively, Cu(ii) complexation might itself promote self-assembly into active ion channels.^15^

**Fig. 1:**
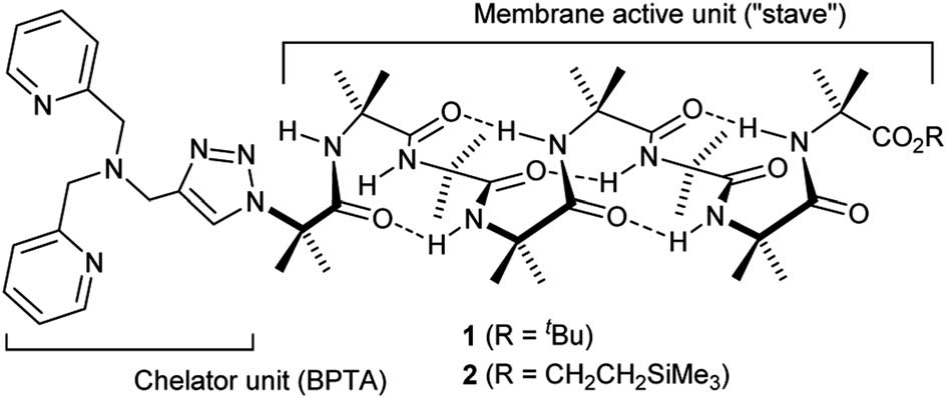
Structure of Aib foldamers **1** and **2**.

The design of the Cu(ii) complexing Aib foldamers **1** and **2** (Fig. 1) combines structural elements from previous studies. Since the ionophoric activity of Aib foldamers is strongly length-dependent, an octameric (Aib_8_) foldamer was used as the core, which had been found to be the minimum length needed to give good activity.18*b* This hydrophobic octamer would fold into a ∼1.6 nm long 3_10_ helix, which would be capped at the N-terminus by an *N*,*N*-bis(pyridin-2′-ylmethyl)-*N*-((1,2,3-triazol-4-yl)methyl)amine (BPTA) chelator unit. The tetrameric Aib foldamer **7** would provide a shorter control compound. We have shown that the structurally related *N*,*N*-bis(quinolin-2-ylmethyl)-*N*-((5′-carboxypyridyl)methyl)amine (BQPA) motif at the N-terminus of foldamers complexes tightly to Cu(ii) and Zn(ii).19b,^23^ Much like related *N*,*N*,*N*-tris(pyridin-2-ylmethyl)amine (TPA) complexes,^24^ the BPTA group should adopt a trigonal pyramidal geometry around the metal ion with at least one potential coordination site for external ligands. It should also be accessible in one synthetic step from readily available Aib foldamers with an N-terminal azido group but different C-terminal groups. Since hydrophobic groups at the C-terminus generally enhance the activity of foldamers in phospholipid vesicles and bacteria,18*a* foldamers **1** and **2** bear respectively a ^*t*^Bu and a CH_2_CH_2_SiMe_3_ terminus.

## Results and discussion

### Synthesis

**Scheme 1:**
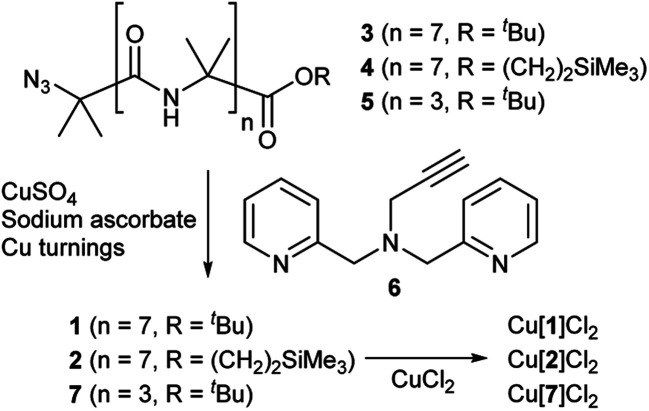
Synthesis of Aib foldamers **1**, **2** and **7** and their corresponding copper(ii) chloride complexes.

Octameric Aib foldamers **3** and **4** were produced by a modification of previous synthetic procedures.^25^ Copper-catalysed azide–alkyne cycloaddition chemistry^26^ was then used to produce the BPTA chelator unit at the N-terminus and give target compounds **1** and **2** (Scheme 1). Tetrameric Aib foldamer **7** was produced from **5** using a similar procedure, providing the control compound with a much shorter membrane-active unit (the “stave”).

Mixing **1** or **2** with CuCl_2_ or CuCl provided green or blue complexes respectively. The green complexes with CuCl_2_, proposed to be Cu[**1**]Cl_2_ and Cu[**2**]Cl_2_, displayed relatively high solubility in water that was unlike other octameric Aib foldamers. The blue complexes from the addition of CuCl gave very broad NMR spectra even after preparing fresh samples, which was ascribed to *in situ* oxidation of Cu(i); Cu(i)(TPA) complexes are very efficient one electron donors and will rapidly reduce alkyl halides^27^ and oxygen.^28^ Furthermore these blue products gave green solids when dried (see the ESI, Fig. S2†).

### X-ray crystallography

**Fig. 2:**
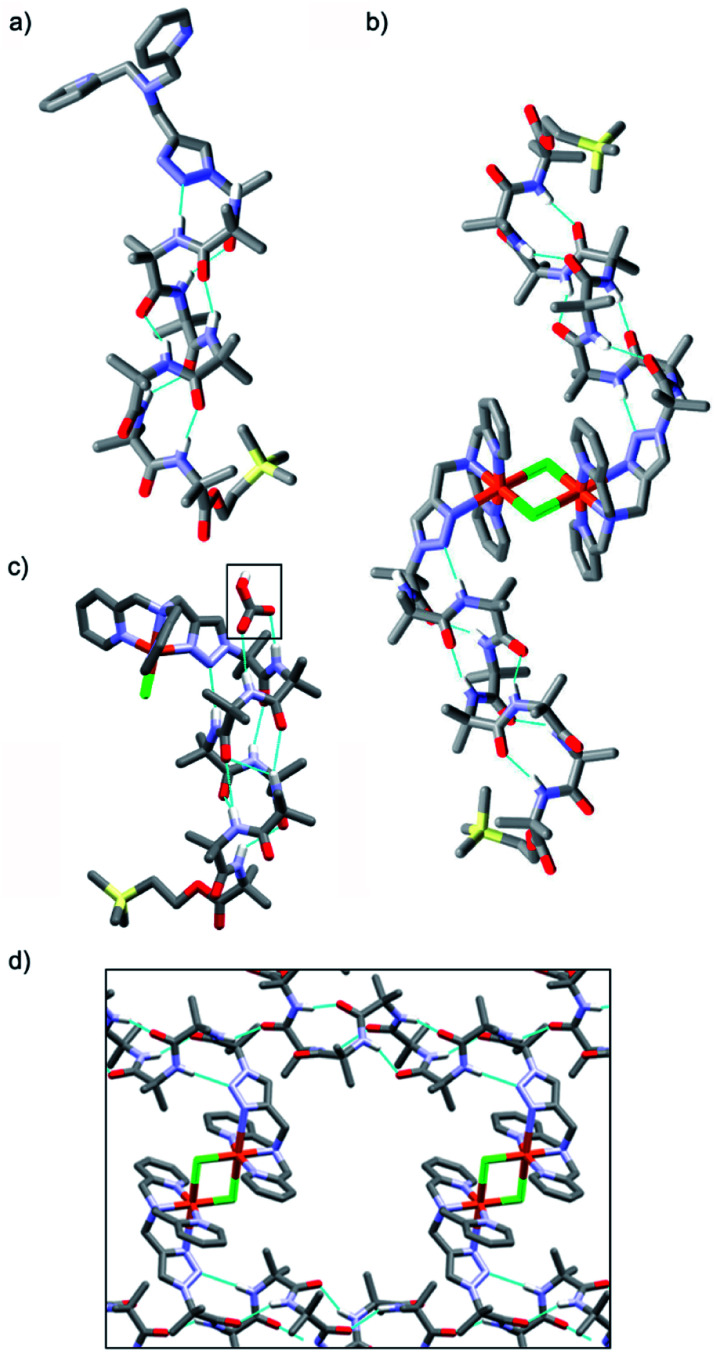
(a and b) X-ray crystal structures of (a) **2**, (b) [Cu(ii)[**2**](μ-Cl)]_2_Cl_2_ and (c) [Cu(ii)[**2**]Cl]·HCO_3_, with bicarbonate ion indicated (boxed). (d) Channels within the X-ray crystal structure of [Cu(ii)[**2**](μ-Cl)]_2_Cl_2_.

Crystal structures were obtained for three compounds: foldamer **2**, the green complex from the addition of CuCl_2_ to **2** and the blue complex from the addition of CuCl to **2**. The solid state structure of **2** shows a 3_10_ helical structure in the foldamer body (Fig. 2a) and a head-to-tail distance of 2.1 nm, which is too short to easily span the hydrophobic width of a typical bilayer (*e.g.* for a typical EYPC/cholesterol bilayer, *ca.* 2.8 nm).^29^

The green complex, Cu[**2**]Cl_2_, that resulted from the addition of CuCl_2_ to **2** crystallized as a dimer of foldamers (Fig. 2b). Two octahedral metal centres share two chloride ligands to give a dimer with a 3.5 nm end-to-end length between the C-termini of the linked 3_10_ helices. The screw sense of the Aib helix inverts at the dicopper centre. The copper ions show a Jahn–Teller distortion that is consistent with Cu(ii), with elongation of one Cu–Cl bond (2.836(2) Å) compared to the other (2.241(2) Å); this distortion around the Cu(ii) centres is similar to that reported in a [Cu(ii)[Ph-TPA](μ-Cl)]_2_Cl_2_ complex.^30^ Electron paramagnetic resonance (EPR) data also showed that unpaired electrons were present (consistent with Cu(ii)). The Cu(BPTA) interacts with the foldamer body through a hydrogen bond between the triazole N2 and the NH of Aib(3). A head-to-tail intermolecular hydrogen bond between dimers (from the NH of Aib(2) to the C=O of Aib(7)) along with side-to-side packing of the dimers in the solid state produces channels (Fig. 2d) that run through the crystal and were filled with disordered electron density. The counterions of the dimeric Cu(ii) complex were not located crystallographically, but an extra chloride was identified by elemental analysis. It is proposed that these chloride anions are located (along with solvent) in a region of disordered electron density that lies in the channels behind the cationic headgroups.

The solid state structure was also determined for crystals obtained from the pale blue product that resulted from CuCl addition to **2**. The bond lengths around the metal ion indicate that a copper(ii) centre and not a copper(i) centre is present (see ESI†). The copper complex has a trigonal bipyramidal geometry, with the copper displaced outwards from the pocket by 0.322 Å, a Cu–Cl bond length of 2.203(3) Å and a copper to central nitrogen distance of 2.055(8) Å. In this structure, the counterion is located (boxed, Fig. 2c). Bicarbonate is found in a region of disordered electron density behind the N-terminal headgroup, where it is hydrogen bonded to both the NH of Aib(2) and the NH of Aib(3) in the foldamer body. This counterion is proposed to result from hydroxide formation during aerial oxidation of an intermediate Cu(i) complex,^28^ followed by sequestration of CO_2_. The foldamer body shows a hydrogen bond between the triazole N2 and the NH of Aib(4). The helix displays a mixture of a 3_10_- and α-helical structure that leads to a shorter end-to-end length of 1.9 nm compared the parent foldamer **2**.^31^ The observation of this monomeric species suggests that the formation of the μ-Cl bridged dimer occurs through a reversible process, which would be influenced by the surrounding environment.

### Ionophoric activity

The membrane activity of **1**, **2**, Cu[**1**]Cl_2_ and Cu[**2**]Cl_2_ was assessed in phospholipid vesicle membranes using 8-hydroxypyrene-1,3,6-trisulfonate (HPTS) assays. HPTS assays can show ionophoric activity that occurs *via* any combination of M^+^/H^+^ antiport, X^–^/OH^–^ antiport, M^+^/OH^–^ symport and X^–^/H^+^ symport.

The HPTS assays of **1**, **2**, Cu[**1**]Cl_2_ and Cu[**2**]Cl_2_ used 1 : 4 cholesterol : egg yolk phosphatidylcholine (EYPC) vesicles in MOPS buffer (pH 7.4) with 100 mM of an appropriate salt (*e.g.* KCl, KBr, NaCl), following previously reported procedures.^16^ The resulting normalised data were fitted to pseudo first-order rate equations as an approximation (see the ESI†).^32^ Although the change in fluorescence after the “burst phase” is likely to arise from multiple processes, including inter-vesicle transfer of foldamers,^33^ this fitting allows the relative effectiveness of each compound to be compared.

**Fig. 3:**
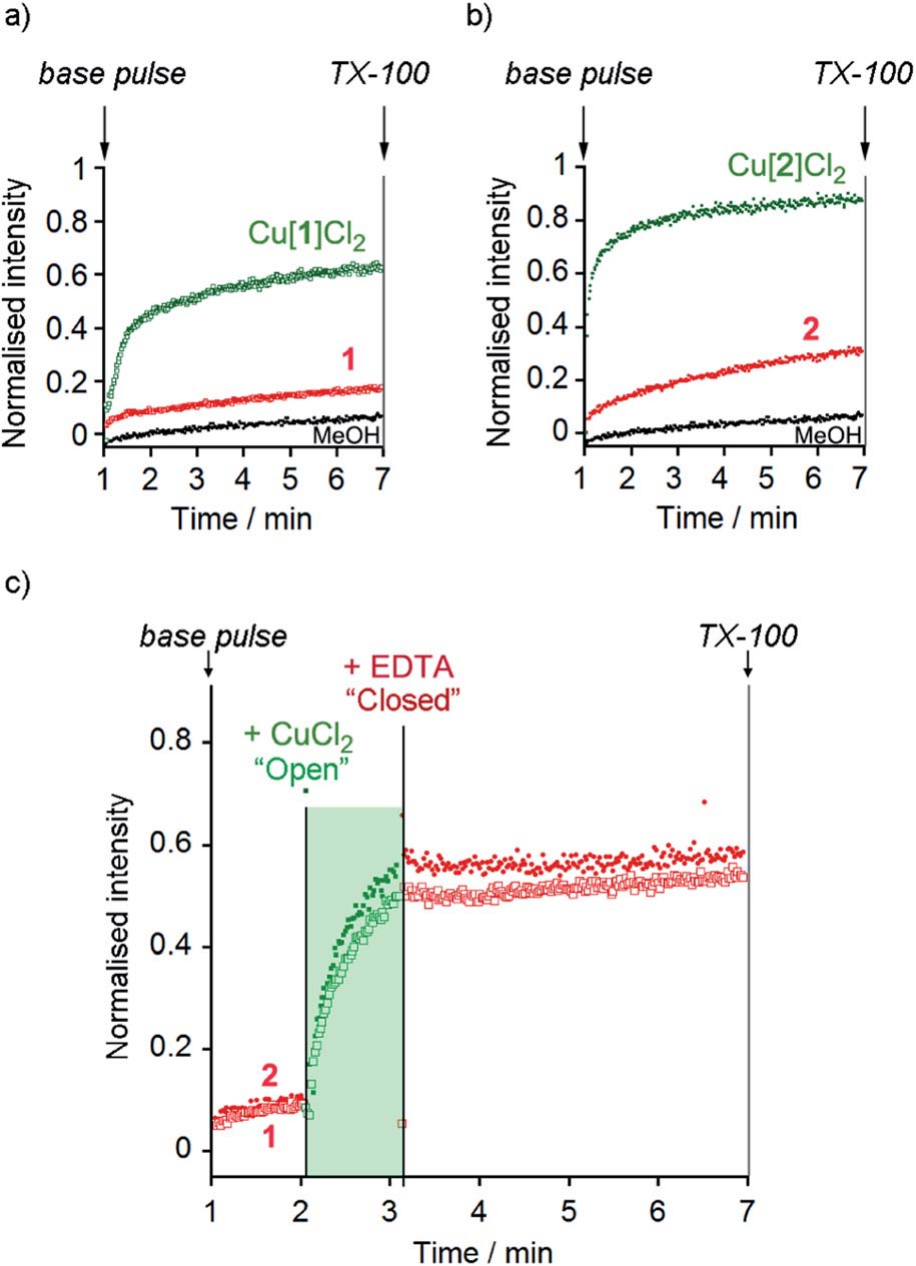
(a and b) HPTS assays of (a) MeOH (•), **1** (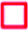), Cu(ii)[**1**]Cl_2_ (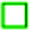) and (b) MeOH (•), **2** (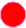) and Cu(ii)[**2**]Cl_2_ (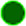) (all 10 μM except Cu(ii)[**2**]Cl_2_ 6 μM) in phospholipid vesicles (760 μM lipid) in the presence of KCl (100 mM). Compounds added at 0 min, base pulse at 1 min, TX-100 added at 7 min allows data normalisation. (c) Switching of ionophoric activity for **1** (10 μM) and **2** (6 μM) in the presence of KCl (100 mM); CuCl_2_ addition (2 eq.) at 120 s followed by EDTA addition (2.2 eq.) at 180 s. Compounds added at 0 min, base pulse at 1 min, TX-100 added at 7 min allows data normalisation. The corresponding data for the addition of MeOH (20 μL) has been subtracted from these data in (c).

At 10 μM foldamer, ion transport by the parent foldamers **1** and **2** was just above the leakage rate cause by addition of the methanol control (an observed constant rate constant, *k*_obs_, of 1 × 10^–3^ s^–1^, Fig. 3a and b). For example, in the presence of KCl, *k*_obs_ was 3 × 10^–3^ s^–1^ for compound **1** and 3.3 × 10^–3^ s^–1^ for compound **2**. The *k*_obs_ values for **1** and **2** in the presence of KBr, KNO_3_ and NaCl were similar to, or less than, *k*_obs_ for KCl (see the ESI†). The CH_2_CH_2_OSiMe_3_ C-terminus (in **2**) led to higher activity than the ^*t*^Bu C-terminus (in **1**), an effect that has previously been noted for other Aib foldamers.18*a*

The Cu(ii) complexes displayed markedly higher activity than the parent foldamers; Cu(ii)[**1**]Cl_2_ (10 μM) showed *ca.* 5-fold higher ionophoric activity for KCl transport (*k*_obs_ = 10.8 × 10^–3^ s^–1^) than **1** after accounting for background leakage. Cu(ii)[**2**]Cl_2_ was too active to be accurately measured at 10 μM, therefore the concentration was reduced to 6 μM. Even at this lower concentration, Cu(ii)[**2**]Cl_2_ showed *ca.* 8-fold higher ionophoric activity for KCl transport (*k*_obs_ = 16.5 × 10^–3^ s^–1^) than **2** at 10 μM. Ionophoric activity was dependent on oligomer length, with the shorter Cu(ii)-Aib tetrameric analogue, Cu(ii)[**7**]Cl_2_, showing a >50% drop in rate compared to Cu(ii)[**1**]Cl_2_ (see the ESI†).

### Switching of ionophoric activity

The marked difference in activity between **1** and **2** and their corresponding Cu(ii) species Cu(ii)[**1**]Cl_2_ and Cu(ii)[**2**]Cl_2_ provides the opportunity to “switch” ion transport on and off. “Switching on” of activity should be induced by addition of CuCl_2_ to compounds **1** and **2**, producing an active Cu(ii) species *in situ*. “Switching off” ionophoric activity might then be possible by subsequent extraction of the Cu(ii) from foldamer **1** or **2**. To demonstrate the switching of ionophoric activity, the HPTS assay was modified so that 2 eq. CuCl_2_ was added at 2 min, followed by addition of 2.2 eq. EDTA at 3 min. As a control, the addition of CuCl_2_ to a methanol-containing vesicle suspension (no foldamer present) showed CuCl_2_ did not significantly affect the HPTS assay or produce leakage (see the ESI†).

Both compounds **1** and **2** were “switched on” by CuCl_2_ addition (Fig. 3c), with a rapid burst of activity that suggests fast ion transport through pores or channels.10*e* Subsequent EDTA addition promptly “switched off” ion transport, only ten seconds after EDTA addition (Fig. 3c). The rapid “switch off” of activity upon the addition of EDTA suggests that the Cu(ii)-foldamer complexes are not fixed at a location deep in the membrane, as EDTA would not be expected to be able to partition deep into the hydrophobic region.

### Characterisation of ionophoric activity

**Fig. 4:**
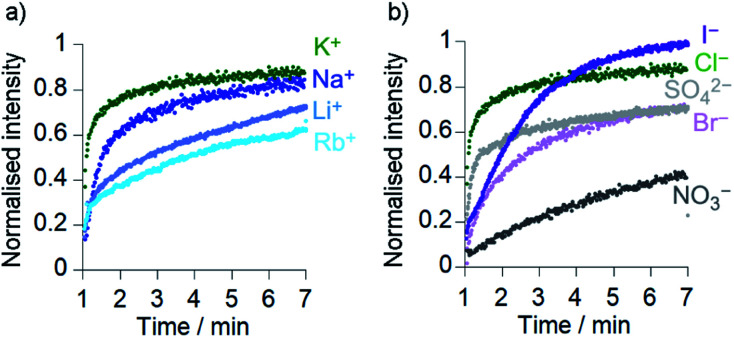
Ionophoric activity of Cu(ii)[**2**]Cl_2_ (6 μM) determined through HPTS assays in the presence of different salts (all 100 mM): (a) KCl (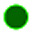), NaCl (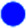), LiCl (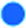), RbCl (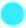); (b) KCl (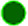), KI (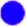), K_2_SO_4_ (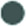), KBr (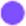), KNO_3_ (•). Compounds added at 0 min, base pulse at 1 min, TX-100 addition at 7 min allows data normalisation.

Comparison of the rates of cation transport by Cu(ii)[**2**]Cl_2_ (Li^+^, Na^+^, K^+^ and Rb^+^, Fig. 4a) and by Cu(ii)[**1**]Cl_2_ (Na^+^ and K^+^, see the ESI†) showed some difference in transport rates for different M^+^, *e.g. k*_obs_ for NaCl was 63% the value of *k*_obs_ for KCl for Cu(ii)[**2**]Cl_2_. However larger rate differences were observed for the transport of anions (Cl^–^, Br^–^, I^–^, NO_3_^–^ and SO_4_^2–^) by Cu(ii)[**2**]Cl_2_, *e.g. k*_obs_ for KNO_3_ was 6% of the value for KCl (Fig. 4b). Cu(ii)[**1**]Cl_2_ shows similar differences between anions, with *k*_obs_ for KCl double that of KBr and 5-fold greater than that of KNO_3_ (see ESI†).

The addition of a selective proton transporter, carbonyl cyanide-4-(trifluoromethoxy)phenylhydrazone (FCCP) only gave a very small increase in the rate of transport by Cu(ii)[**1**]Cl_2_ and Cu(ii)[**2**]Cl_2_ (although significant rate increases were observed for transport by **1** and **2**; see the ESI†). The small effect of FCCP on Cu(ii)[**1**]Cl_2_ and Cu(ii)[**2**]Cl_2_ activity suggests proton transport is not rate limiting.^34^

**Fig. 5:**
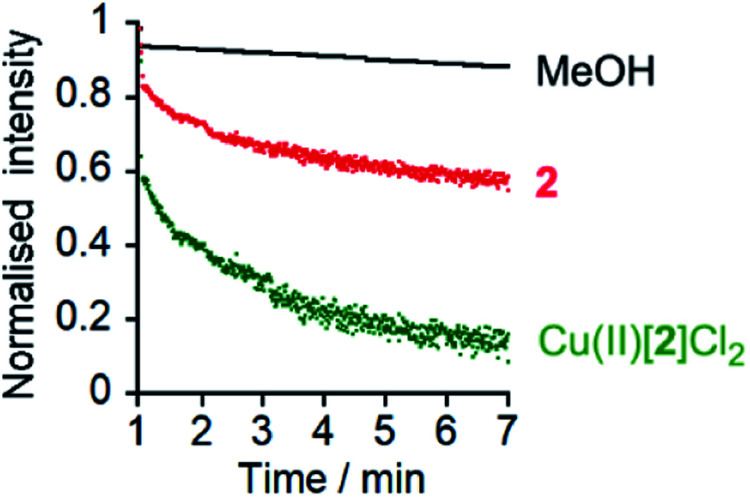
Lucigenin assays of Cl^–^ transport by MeOH (•), **2** (10 μM, 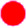) and Cu(ii)[**2**]Cl_2_ (10 μM, 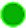) with interior NaNO_3_ (200 mM) and exterior NaCl (2 M). Compounds added at 0 min, NaCl added at 1 min, TX-100 addition at 7 min allows data normalization.

To confirm that chloride could pass through the bilayers, lucigenin assays5*a* were performed for **2**, Cu(ii)[**1**]Cl_2_ and Cu(ii)[**2**]Cl_2_ (Fig. 5 and ESI, Fig. S10†). The same lipids were used as for the HPTS assays and lucigenin was encapsulated with NaNO_3_ in the vesicles. Both Cu(ii)[**1**]Cl_2_ and Cu(ii)[**2**]Cl_2_ showed faster Cl^–^ transport than **2**, albeit with a smaller difference between **2** and Cu(ii)[**2**]Cl_2_ than in the HPTS assays (Fig. 5, *cf.*Fig. 3). The chloride transport activities are lower than those of the thioureas developed by Gale, Davis and co-workers^35^ but comparable to those of Talukdar and co-workers fumaramides (up to 40% transport after 3 minutes at 30 μM fumaramide).^36^

### Mechanism of ionophoric activity

Cation/anion carrier,^37^ ion channel/pore,21a,^38^ and membrane disruption mechanisms^39^ have all been observed for Aib-rich ionophores, so a series of assays were performed to determine which mechanism predominated for these foldamers.

To assess if these compounds can act as cation carriers, U-tube experiments for sodium picrate transport were performed on **1**, Cu(ii)[**1**]Cl_2_ and Cu(ii)[**2**]Cl_2_. No transport across the bulk phase was observed for any of these foldamers after 24 h, unlike the dibenzo-18-crown-6 positive control (see the ESI, Fig. S11†). Similarly, a U-tube experiment for chloride transport was performed using lucigenin.^4^,^40^ This showed no chloride was carried through the organic phase by **2**, Cu(ii)[**1**]Cl_2_ and Cu(ii)[**2**]Cl_2_, unlike the 2-aminopentane positive control (see the ESI, Fig. S12†). These assays suggest that these Aib foldamers do not predominantly act *via* an ion carrier mechanism.

A 5/6-carboxyfluorescein (5/6-CF) release assay18*b* was then performed to assess if Cu(ii)[**1**]Cl_2_ and Cu(ii)[**2**]Cl_2_ disrupt the membrane or form very large pores able to accommodate the passage of 5/6-CF (∼10 Å diameter from molecular modelling).^41^ Despite high activity in the HPTS assays at 10 μM foldamer, dye release was insignificant in both cases (see the ESI, Fig. S13†), indicating that membrane disruption is not extensive.

To assess if the most active compound, Cu(ii)[**2**]Cl_2_, and its parent foldamer **2** form channels, planar bilayer conductance (PBC) studies were carried out. The observation of discrete conductance events can indicate channel formation and provide information on the nature of these channels.

**Fig. 6:**
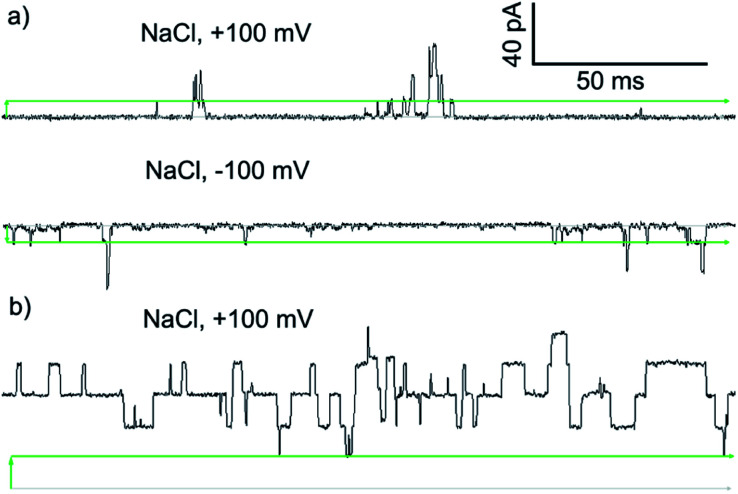
Step changes in conductance induced by Cu(ii) complexes of **2** added to *cis* side of the membrane. Membranes were formed from EYPC lipid/cholesterol (4 : 1, w/w) in MOPS buffer at room temperature (20 mM MOPS, 100 mM NaCl, pH 7.4). Change from 0 pA (grey bars) indicated by green arrows. Green bars mark the approximate level of a single quantized current-step. (a) Green complex ([Cu(ii)[**2**](μ-Cl)]_2_Cl_2_, final concentration 8.3 μM). (b) Blue complex ([Cu(ii)[**2**]Cl]·HCO_3_, final concentration 8.3 μM).

PBC experiments were performed in a custom-built cell, with either **2** or Cu(ii)[**2**]Cl_2_ (5–10 μL of a 1 mM solution in MeOH) added to the ground well. Characterisation sweeps were continued for 2 h, or until substantial channel-forming activity was observed. Single channel experiments were conducted under an applied potential of +100 or –100 mV in 50 s sweeps. Under these conditions, parent foldamer **2** did not display any ion channel behaviour after 2 h under these conditions (see the ESI, Fig. S16†). In contrast, discrete channel-forming behaviour was observed for Cu(ii)[**2**]Cl_2_ (Fig. 6a), with current levels only slightly higher in KCl than in NaCl. Large current levels and reproducible well-defined quantized steps from 0.5 to 5 ms in duration (similar to the “flicker” behaviour described by Chui and Fyles^42^) were measured (Fig. 6). Greater increases in macroscopic current value were observed under a negative applied potential rather than a positive applied potential, which could suggest a positive charge is being driven into (or through) the membrane by the applied negative potential; analogous behaviour has been observed for other channel-forming compounds bearing positive charges.^43^ This could imply [Cu(ii)[**2**]Cl]^+^ cations^44^ are involved in the channel-forming species. Channel events have multilevel conductances, both at +100 mV (∼0.08 nS, ∼0.14 nS) and at –100 mV potentials (∼0.08 nS, ∼0.15 nS).

The *I*–*V* curve for Cu(ii)[**2**]Cl_2_ in KCl is not linear, and shows sharp increases in conductance as the potential is increased to +100 mV or –40 mV with a progressive increase in current after repeated sweeps between these potentials (see the ESI, Fig. S17†). The increase in conductance over time may be due to slow insertion of the foldamer into the membrane, a process that is accelerated as the potential difference increases (especially for negative potentials).^45^

The blue product from the addition of CuCl to **2** was also assessed using PBC experiments. Under the same conditions used for Cu(ii)[**2**]Cl_2_, Cu(ii)[**2**]Cl·HCO_3_ showed a mixture of behaviours. Short-lived “flicker”-type openings could be observed, which displayed conductance levels (0.07 ± 0.01 nS, 0.16 ± 0.01 nS, see the ESI†) similar to those observed for Cu(ii)[**2**]Cl_2_. In addition, much longer lived “square topped” openings could also be observed (Fig. 6b). These very regular well-defined quantized current steps were open for up to 20 ms at +100 mV, with multiple stepwise increases in conductance with ∼0.18 nS increments.^46^ A linear increase in the conductance of these current steps was observed with increasingly positive applied potential, suggesting channels with a symmetrical charge distribution are formed (see the ESI†).7*b*

Application of Hille's equation^47^ to estimate the inner channel diameters for both Cu(ii)[**2**]Cl_2_ and Cu(ii)[**2**]Cl·HCO_3_ provided approximate pore diameters of 1.5 nm, 2.1 nm and 2.3 nm for the different conductance levels (see the ESI†). These diameters are comparable to those proposed for different alamethicin pores (1.1 nm for hexameric pores; 1.8 nm for octameric pores).^38^,^48^

### Antibiotic activity

Several synthetic ion channels have been shown to exhibit biological activity, most commonly in the form of antibiotic activity, which correlates with ion channel activity in non-biological membranes.8b,10h,14f,^49^ Long Aib_*n*_ foldamers (*n* > 10) show good activity against the Gram-positive *B. megaterium* strain DSM319.18*a* N-terminal functionalisation of such Aib foldamers with BPTA will provide a new type of amino terminal Cu(ii)- and Ni(ii)-binding (ATCUN) motif, which may improve antimicrobial properties.^50^ Like other ATCUN motifs, the Cu(ii) will be tightly complexed in the BPTA pocket of **1** and **2** (the stability constant for Cu(ii) with TPA is >10^16^ M^–1^),50a,^51^ preventing the release of potentially toxic free Cu(ii) ions into the blood.

**Fig. 7:**
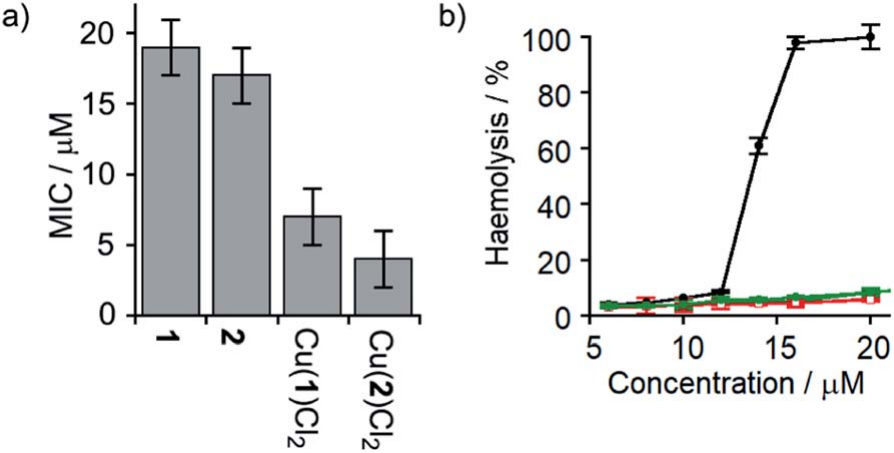
(a) MIC against *B. megaterium* strain DSM319. (b) Haemolysis of human erythrocytes caused by alamethicin (•) and **1** (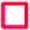), Cu(ii)[**1**]Cl_2_ (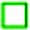), **2** (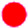) and Cu(ii)[**2**]Cl_2_ (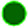).

The antibiotic activities of **1**, **2**, Cu(ii)[**1**]Cl_2_ and Cu(ii)[**2**]Cl_2_ were measured against *B. megaterium* strain DSM319 (see the ESI†). The complexes Cu(ii)[**1**]Cl_2_ and Cu(ii)[**2**]Cl_2_ showed much lower minimum inhibitory concentrations (MICs) than **1** and **2** (Fig. 7a), which correlates inversely with the relative ionophoric activities measured by HPTS assays. Interestingly, the MIC for Cu(ii)[**2**]Cl_2_ is the same within error as that for alamethicin under the same conditions against this Gram-positive bacterial strain (alamethicin showed a MIC of 6 ± 2 μM, see ESI†).

Although alamethicin is a very active channel former, its use in the clinic is hampered by its high haemolytic activity.^52^ Since Cu(ii)[**2**]Cl_2_ shows similar antibiotic activity to alamethicin and also bears a cationic centre (which often produce haemolysis),^53^ its haemolytic activity against human erythrocytes was assessed. The dose response curve for haemoglobin release gave an EC_50_ value for haemolysis by alamethicin of 1.4 μM, similar to its reported EC_50_ value (1.45 μM in phosphate buffer after 1 h incubation).^54^ Interestingly, all foldamers showed much lower haemolytic activity under these conditions than alamethicin, although no EC_50_ values could be determined for the Aib foldamers due to their solubility limits (20 μM for **1** and **2**; the more water soluble Cu(ii) complexes produced only 10% haemolysis at 25 μM). It is clear however that Cu(ii)[**1**]Cl_2_ and Cu(ii)[**2**]Cl_2_ both produce low haemolysis below 20 μM, despite their positively charged centres^55^ and high ionophoric/antibiotic activity at this concentration. It is known that the haemolytic activity of amphipathic peptides, including alamethicin, decreases substantially on a reduction of peptide hydrophobicity and hydrophobic moment.^56^ We suggest that the lower haemolytic activity of Cu(ii)[**1**]Cl_2_ and Cu(ii)[**2**]Cl_2_ compared to alamethicin is due to their lower hydrophobicity, arising from the shorter length and the cationic N-termini of the Cu(ii) complexes of **1** and **2**, yet ionophoric activity is maintained due to stronger interactions between foldamers.

## Conclusions

The chelating Aib foldamers described here show remarkably efficient “switching” of ion transport activity. As observed for unfunctionalised Aib octamers,18*b* BPTA-capped Aib octamers showed measurable ionophoric activity at micromolar concentrations. However complexation to CuCl_2_ markedly increased this ionophoric activity, with the Cu(ii) complexes showing 5- to 8-fold increases in activity compared to the parent octamers. There was also clear length dependence, with a shorter Aib tetramer showing markedly lower activity. Complexation of Cu(ii) was reversible, allowing the ionophoric activity of the Aib octamers **1** and **2** to be activated *in situ* by addition of CuCl_2_ and deactivated by extraction of the Cu(ii) by EDTA. The ionophoric activity of the CuCl_2_ complex of **2** approaches that measured for the archetypical peptaibol alamethicin.18*b* Channels formed by these CuCl_2_/Aib octamer complexes appear to transport both cations and anions, and showed good chloride transport activity in lucigenin assays. PBC studies showed square-topped conductance profiles for [Cu(ii)[**2**](μ-Cl)]_2_Cl_2_ and [Cu(ii)[**2**]Cl]·HCO_3_ but no conductance for the parent foldamer **2**, consistent with multimeric ion channel formation by the Cu(ii) complexes.

The observation of hydrogen bonds and Cu–Cl–Cu bridges between foldamers in the solid state (Fig. 2b) indicates the Cu(ii) complexes of the foldamers can form multiple strong intermolecular interactions. These interactions will promote self-assembly of monomeric foldamers into multimeric channels within the membrane, perhaps aided by the absence of competing ligands like water and the high effective concentrations that result after partitioning into the membrane.^57^ The solid-state structure of [Cu(ii)[**2**]Cl]·HCO_3_ also suggests the Aib foldamer body has a role in facilitating the passage of ions through the bilayer, and may favour one ion over another. This structure shows that the bicarbonate counterion is not bound to the Cu(ii) centre but is located behind the headgroup, where it is hydrogen-bonded to the Aib foldamer body.

The antibiotic activities of **1**, **2**, Cu(ii)[**1**]Cl_2_ and Cu(ii)[**2**]Cl_2_ correlate well with their relative ion channel activity. The MICs of Cu(ii)[**1**]Cl_2_ and Cu(ii)[**2**]Cl_2_ were similar to that of alamethicin. However, these ionophores did not show the high haemolytic activity of alamethicin, a significant barrier to the adoption of peptaibols in the clinic.

These Aib foldamers show remarkably efficient switching of channelling activity upon Cu(ii) complexation/decomplexation. This activity switching in synthetic membranes, although not easily applied *in vivo*, may indicate a pathway towards switchable non-haemolytic peptaibol antibiotics and switchable drugs for treating the symptoms of channelopathies.

## Author contributions

ADP and SJW conceived the study. ADP and ML performed the synthesis with advice from JC. GFSW and IJV-Y carried out the crystallography. ADP and FdS performed the ionophoric activity assays in vesicles. SB, DFC-G and SLC performed and analysed the PBC assays. ADP and ET carried out the antibiotic studies. ADP and JB carried out the haemolysis assays. ADP, FdS and SJW wrote the manuscript with input from all authors.

## Conflicts of interest

There are no conflicts to declare.

## Supplementary Material

Supplementary information

Crystal structure data
