# Supplementary material for: Switchable foldamer ion channels with antibacterial activity
Source: Chem Sci. 2020 Jun 4;11(27):7023–30. doi: 10.1039/d0sc02393k (PMC7481839; doi:10.1039/d0sc02393k)
Supplement: Supplementary file 1 [file SC-011-D0SC02393K-s001.pdf]

Electronic Supplementary Material (ESI) for Chemical Science.
This journal is © The Royal Society of Chemistry 2020

Electronic Supporting Information

Switchable foldamer ion channels with antibacterial activity

Anna D. Peters,[a,b] Stefan Borsley,[a,c] Flavio della Sala,[a,b] Dominic F. Cairns-Gibson,[c] Marios Leonidou,[a,b] Jonathan Clayden,[d] George F. S. Whitehead,[a] Iñigo J. Vitórica-Yrezábal,[a] Eriko Takano,[a,b] John Burthem,[e,f] Scott L. Cockroft,[c] and Simon J. Webb[a,b,*]

a) *Department of Chemistry, University of Manchester, Oxford Road, Manchester M13 9PL, United Kingdom.*
b) *Manchester Institute of Biotechnology, University of Manchester, 131 Princess St, Manchester M1 7DN, United Kingdom. E-mail: S.Webb@manchester.ac.uk; Tel: +44 161 306 4524.*
c) *EaStCHEM School of Chemistry, University of Edinburgh, Joseph Black Building, Edinburgh, UK*
d) *School of Chemistry, University of Bristol, Cantock's Close, Bristol BS8 1TS, UK.*
e) *Department of Haematology, Manchester Royal Infirmary, Manchester University NHS Foundation Trust, Manchester M13 9WL, United Kingdom*
f) *Division of Cancer Sciences, School of Medical Sciences, University of Manchester, Manchester, United Kingdom*

* Email: S.Webb@manchester.ac.uk

TABLE OF CONTENTS

1. Chemical synthesis: instruments **4**

2. Chemical synthesis: materials **5**

2.1 Abbreviations 5

2.2 Materials 5

2.3 General experimental procedures 5
2.3.1 General procedure 1: Hydrogenation of N_3Aib_nOR (for n = 1-4) 5
2.3.2 General procedure 2: Synthesis of N_3Aib_nOR 5
2.3.3 General procedure 3: Synthesis of N_3Aib_nOH 6
2.3.4 General procedure 4: Formation of the active ester with EDC·HCl 6
2.3.5 General procedure 5: Synthesis of $N_3Aib_nO^tBu$ by reaction of active ester 6

3. Synthetic procedures **7**

3.1 2-Bromo-2-methylpropionic acid 7

3.2 N_3AibO^tBu 7

3.3 NH_2AibO^tBu 7

3.4 $N_3Aib_2O^tBu$ 7

3.5 $H_2NAib_2O^tBu$ 7

3.6 $N_3Aib_3O^tBu$ 7

3.7 $NH_2Aib_3O^tBu$ 8

3.8 $N_3Aib_4O^tBu$ 8

3.9 $NH_2Aib_4O^tBu$ 8

3.10 N_3AibOH 8

3.11 $NH_2AibO(CH_2)_2TMS$ 8

3.12 $N_3Aib_2O(CH_2)_2TMS$ 9

3.13 $NH_2Aib_2O(CH_2)_2TMS$ 9

3.14 $N_3Aib_3O(CH_2)_2TMS$ 9

3.15 $NH_2Aib_3O(CH_2)_2TMS$ 9

3.16 $N_3Aib_4O(CH_2)_2TMS$ 9

3.17 $NH_2Aib_4O(CH_2)_2TMS$ 9

3.18 $N_3Aib_8O^tBu$ 10

3.19 $N_3Aib_8O(CH_2)_2TMS$ 10

3.20 *N*,*N*-Bis(pyridin-2-ylmethyl)propargylamine 10

3.21 Aib foldamer **1** 11

3.22 Aib foldamer **2** 12

3.23 Aib foldamer **7** 12

3.24 Conditions for complexation of CuCl with foldamer **1** 13

3.25 Conditions for complexation of CuCl with foldamer **2** 13

3.26 Conditions for complexation of CuCl with foldamer **7** 14

3.27 ^1H NMR spectrum of product from complexation of CuCl with foldamer **2** 14

3.28 Discolouration of product from complexation of CuCl with foldamer **7** 14

3.29 Complex Cu(II)[**1**]Cl_2 15

3.30 Complex Cu(II)[**2**]Cl_2 15

3.31 Complex Cu(II)[**7**]Cl_2 16

4. ^{1}H and ^{13}C NMR spectra of synthesized compounds 17

4.1.1 Foldamer **1** 17
4.1.2 Foldamer **2** 19
4.1.3 Foldamer **7** 20

5. Vesicle studies 21

5.1 Preparation of large unilamellar vesicles for HPTS assays 21

5.2 Procedure for HPTS assays 21
5.2.1 Procedure for the determination of first order rate constants 22
5.2.2 $CuCl_2$ gating of ionophoric activity of **1** and **2** 23
5.2.3 Effect of $CuCl_2$ addition on the HPTS assay 23
5.2.4 HPTS curves for **1**, **2**, Cu(II)[**1**]Cl_2, Cu(II)[**2**]Cl_2, Cu(II)[**7**]Cl_2 with different salts 25

5.3 Carbonyl cyanide-*p*-trifluoromethoxyphenylhydrazone (FCCP) data 26

5.4 Preparation of large unilamellar vesicles for lucigenin assays 27

5.5 Procedure for lucigenin assays 27

5.6 Curve fitting of lucigenin assays and data for Cu(II)[**1**]Cl_2 28

5.7 Preparation of large unilamellar vesicles for CF release assays 29

5.8 U-tube metal picrate transport experiments 29

5.9 U-tube lucigenin transport experiments 29

5.10 Procedure for CF assays 30

6. Planar bilayer conductance (PBC) studies 31

6.1 Procedure 31

6.2 PBC data for foldamer **2** and complex Cu(II)[**2**]Cl_2 33

6.3 I-V curves for EYPC lipid/cholesterol bilayers with and without foldamer **2** 34

6.4 Repeated I-V curves for EYPC lipid/cholesterol bilayers with complex Cu(II)[**2**]Cl_2 35

6.5 PBC data for the product from complexation of CuCl with **2** (Cu(II)[**2**]Cl.HCO_3) 36
6.5.1 Short lived openings 37
6.5.2 Long lived openings 38

6.6 Estimation of channel diameter 41

7. Antimicrobial assays 43

7.1 Antimicrobial assays: minimum inhibitory concentrations (MIC) 43

7.2 Haemolysis assays 43

8. Crystal data and structure refinement 45

8.1 Foldamer **2** 45

8.2 Copper complexes 47
8.2.1 Complex Cu(II)[**2**]Cl.HCO_3 47
8.2.2 Complex Cu(II)[**2**]Cl_2 49

9. Electron paramagnetic resonance (EPR) studies 51

10. References 53

1. Chemical synthesis: instruments

Nuclear Magnetic Resonance (NMR) spectra were performed on a Bruker Ultrashield 400 MHz spectrometer in appropriate deuterated solvents. Tetramethylsilane (TMS) was used as an internal standard to record ^{1}H and ^{13}C NMR. Coupling constants (*J*) are reported in Hertz (Hz) and rounded to 0.1 Hz. Chemical shifts (δ) were measured in parts per million (ppm). Splitting patterns are illustrated as follows: singlet (s), doublet (d), triplet (t), multiplet (m), broad (br) or some combination of these. Assignments of the peaks were performed by chemical shifts, coupling constants, DEPT, COSY, HSQC and HMBC data.

Infrared spectra were measured on a Thermo Scientific Nicolet iS5 FTIR Spectrometer.

Mass spectra were recorded by staff at the University of Manchester on a Waters Platform II using ElectroSpray Ionisation (ESI).

Semi-preparative high pressure liquid chromatography (HPLC) purification was performed on HPLC Agilent 1100 series equipped with a semi-preparative C18 column Agilent eclipse XDB-C18, 5 µm, 9.4 mm × 250 mm.

Optical rotation ($[\alpha]_D^T$) measurements were taken on a AA-100 polarimeter at 20 °C using a cell with a pathlength of 0.25 dm. The solvent and concentration are stated with individual readings. Melting points (mp) were determined on a GallenKamp apparatus and are uncorrected. Infra-red spectra (IR) were recorded on an ATi Perkin Elmer Spectrum RX1 FT-IR. Only absorption maxima (ν_{max}) of interest are reported and quoted in wavenumbers (cm^{-1}). Low- and high-resolution mass spectra were recorded by staff at the University of Manchester. Electrospray (ES) spectra were recorded on a Waters Platform II. High-resolution mass spectra (HRMS) were recorded on a Thermo Finnigan MAT95XP and are accurate to ±0.001 Da. HPLC analyses were performed on an Agilent 1100 Series instrument equipped with a Chiralpak® AD-H (0.46 × 25 cm) column.

2. Chemical synthesis: materials

2.1 Abbreviations

The following abbreviations have also been used; NEt_3 = triethylamine, DMAP = 4-dimethylaminopyridine, Aib = aminoisobutyric acid, DIPEA = *N,N*-diisopropylethylamine, EDC·HCl = *N*-(3-dimethylaminopropyl)-1-*N′*-ethylcarbodiimide, EtOAc = ethyl acetate, EtOH = ethanol, HOBt = 1-hydroxybenzotriazole, MeOH = methanol.

2.2 Materials

All reactions were carried out in oven-dried glassware under an atmosphere of nitrogen using standard anhydrous techniques. Anhydrous THF was obtained by drying with activated 4Å molecular sieves. Anhydrous acetonitrile and *N*,*N*-dimethylformamide (DMF) were purchased from Sigma-Aldrich. Petrol refers to the fraction of light petroleum ether boiling between 40 and 60 °C. Dichloromethane and toluene were dried over molecular sieves 4 Å under a nitrogen atmosphere. All other solvents and commercially available reagents were used as received without further purification

All products were dried on a rotary evaporator followed by connection to a high vacuum system to remove any residual solvent. Flash chromatography was performed on silica gel (Merck 60H, 40-60 nm, 230–300 mesh). Analytical thin layer chromatography (TLC) was performed on Macherey Nagel alugram SIL G/UV254 and were visualised by UV (254 nm), ninhydrin or potassium permanganate dyes where appropriate.

2.3 General experimental procedures

2.3.1 General procedure 1: Hydrogenation of N_3Aib_nOR (for n = 1-4)

N_3Aib_nOR (1 eq) was dissolved in EtOH (5 mL/1 mmol) under a nitrogen atmosphere. Pd/C (10%, 10mg/1 mmol) was added and the reaction mixture stirred under an atmosphere of H_2 until IR indicated complete consumption of the starting material (24 h). The mixture was washed through a pad of Celite with EtOAc and the filtrate concentrated.

2.3.2 General procedure 2: Synthesis of N_3Aib_nOR

N_3AibOH (2 eq) was dissolved in dry CH_2Cl_2 (4 mL/1 mmol). EDC*HCl (2 eq) was added in portions and stirred for 15 min. Afterwards, HOBt (0.1 eq) and Et_3N (1.2 eq) was added dropwise and stirred for another 15 min. NH_2Aib_nOR (1 eq) was added and stirred at room temperature overnight. The reaction mixture was washed with 5% aq. $KHSO_4$ solution (2 × 20 mL), sat. $NaHCO_3$ solution (2 × 20 mL). The aqueous layer was washed with EtOAc (2 × 10 mL). The organic layers were combined and dried over $MgSO_4$, filtrated and the solvent was removed under reduced pressure.

2.3.3 General procedure 3: Synthesis of N_3Aib_nOH

$N_3Aib_nO^tBu$ (1 eq) was dissolved in a 1:2 mixture of trifluoroacetic acid (1 mL/1 mmol) and CH_2Cl_2 (2 mL/1mmol). The resulting reaction mixture was stirred at room temperature for 24 h under a nitrogen atmosphere. The solvent was removed and the yellow high viscous oil was re-dissolved in Et_2O (5 × 30 mL) and the solvent was removed.

2.3.4 General procedure 4: Formation of the active ester with EDC·HCl

Aib_nOH (1 eq) in was dissolved in dry CH_2Cl_2 (2 mL/1mmol) and EDC·HCl (2 eq) was added slowly. To the resulting reaction mixture was stirred at room temperature overnight under a nitrogen atmosphere. The solution was diluted with CH_2Cl_2 (5 mL/1mmol) and the organic phase was washed with sat. $NaHCO_3$ (2 × 5 mL/mmol) and brine (5 mL/mmol). The organic layer was dried over $MgSO_4$, filtered and the solvent removed.

2.3.5 General procedure 5: Synthesis of $N_3Aib_nO^tBu$ by reaction of active ester

Aib_n-active ester (1 eq) was dissolved in MeCN (2 mL/1 mmol) and NEt_3 (1.2 eq) was added slowly. Afterwards the appropriate amine (1.0 eq) was added. The resulting reaction mixture was heated to reflux and stirred for 48 h. The solvent was removed and the resulting residue was purified by flash column chromatography on silica (SiO_2) with the appropriate solvent system.

AibOH	NH_2AibO^tBu	EDC	HOBt	NEt_3	Yield %
1.5	1.0	1.2	0.2	1.5	65
1.5	1.0	1.4	0.2	1.5	65
1.5	1.0	1.6	0.2	1.5	65
1.5	1.0	1.8	0.2	1.5	71
1.5	1.0	2.0	0.2	1.5	75
2.0	1.0	1.2	0.1	2.0	80
2.0	1.0	1.4	0.1	2.0	84
2.0	1.0	1.6	0.1	2.0	84
2.0	1.0	1.8	0.1	2.0	85
2.0	1.0	2.0	0.1	2.0	89

3. Synthetic procedures

3.1 2-Bromo-2-methylpropionic acid

2-Bromo-2-methylpropionic acid (15.0 g, 89.9 mmol) was dissolved in dry DMF (60 mL). To this solution sodium azide (8.75 g, 134.7 mmol) was slowly added and the resulting mixture stirred at room temperature for 72 h under a nitrogen atmosphere. The reaction mixture was diluted with H_2O (40 mL) forming a clear solution and acidified to pH = 2 with 1 M HCl (30 mL) and extracted with *tert*-butyl methyl ether (3 × 50 mL). The organic layer was washed with 1 M HCl (4 × 20 mL), dried over $MgSO_4$, filtered and the solvent removed to yield the title compound as a pale yellow oil, which was used in the next step without further purification (11.34 g, 87.9 mmol, 98%). **^{1}H NMR** (400 MHz, $CDCl_3$, 298 K): δ = 1.46 (s, 9 H, 2 × CH_3) ppm; **^{13}C NMR** (100 MHz, $CDCl_3$, 298 K): δ = 178.9 (CO), 62.8 (C), 24.3 (CH_3) ppm. Spectroscopic data is consistent with the reported data in the literature.[1]

3.2 N_3AibO^{t}Bu

Sodium azide (6.65 g, 100.8 mmol) was dissolved in dry DMF (50 mL). To the resulting white suspension tert-butyl-2-bromo-2-methylpropionate (12.5 mL, 67.2 mmol) was added and the reaction mixture was stirred at room temperature for 72 h under a nitrogen atmosphere. The resulting white suspension was diluted with H_2O (40 mL) and acidified to pH = 2 with 1M HCl (30 mL) and extracted with tert-butyl methyl ether (3 × 50 mL). The organic layer was washed with 1M HCl (4 × 20 mL), dried over $MgSO_4$, filtered and the solvent removed to yield the title compound as a pale yellow oil, which was used in the next step without further purification (12.10 g, 64.8 mmol, 96%). **^{1}H NMR** (400 MHz, $CDCl_3$, 298 K): δ = 1.43 (s, 9 H, 3 × CH_3), 1.35 (s, 6 H, 2 × CH_3) ppm; **^{13}C NMR** (100 MHz, $CDCl_3$, 298 K): δ = 171.8 (CO), 82.4 (C), 63.4 (C), 27.9 (CH_3), 24.3 (CH_3) ppm. Spectroscopic data is consistent with the reported data in the literature.[1]

3.3 NH_2AibO^{t}Bu

N_3AibO^{t}Bu (5.90 g, 30.75 mmol) was hydrogenated using procedure 1 and the product was isolated as a pale yellow oil. (4.82 g, 30.27 mmol, 98%). **^{1}H NMR** (400 MHz, $CDCl_3$, 298 K): δ = 1.43 (s, 9 H, 3 × CH_3), 1.35 (s, 6 H, 2 × CH_3) ppm; **^{13}C NMR** (100 MHz, $CDCl_3$, 298 K): δ = 177.4 (CO), 80.6 (C), 55.0 (C), 27.9 (CH_3), 27.7 (CH_3) ppm. Spectroscopic data is consistent with the reported data in the literature.[1]

3.4 N_3Aib$_2$O^{t}Bu

H_2N-AibO^{t}Bu (2.40 g, 15.1 mmol) was used following procedure 2 and the product was isolated as a pale yellow oil. (3.7 g, 14.1 mmol, 93%).**^{1}H NMR** (400 MHz, $CDCl_3$, 298 K): δ = 7.03 (s, 1 H, NH), 1.45 (s, 6 H, 2 × CH_3), 1.44 (s, 6 H, 2 × CH_3), 1.39 (s, 9 H, 3 × CH_3) ppm; **^{13}C NMR** (100 MHz, $CDCl_3$, 298 K): δ = 173.5 (CO), 171.1 (CO), 81.7 (C), 56.7 (C), 27.8 (CH_3), 24.3 (CH_3) ppm. Spectroscopic data is consistent with the reported data in the literature.[1]

3.5 H_2NAib$_2$O^{t}Bu

N_3Aib$_2$O^{t}Bu (1.92 g, 7.10 mmol) was hydrogenated following procedure 1 and the product was isolated as a white solid (1.56 g, 6.38 mmol, 90 %). **^{1}H NMR** (400 MHz, $CDCl_3$, 298 K): δ = 7.98 - 8.03 (m, 1 H), 1.44 (s, 6H, 2 × CH_3), 1.39 (s, 9 H, 2 × CH_3), 1.28 (s, 6H, 2 × CH_3) ppm; **^{13}C NMR** (100 MHz, $CDCl_3$, 298 K): δ = 176.0 (CO), 174.0 (CO), 81.2 (C), 56.2 (C), 54.8 (C), 29.0 (CH_3), 27.9 (CH_3), 24.5 (CH_3) ppm. Spectroscopic data is consistent with the reported data in the literature.[1]

3.6 N_3Aib$_3$O^{t}Bu

H_2N-Aib$_2$OtBu (3.41 g, 13.9 mmol) was coupled following procedure 2 and purified by flash chromatography (SiO_2/petrol ether:EtOAc (85:15)). The product was isolated as a white solid (3.15 g, 11.6 mmol, 83 %). **TLC**

SiO_2/Petrolether/EtOAc (85:15) R_f = 0.20; ^{1}**H NMR** (400 MHz, $CDCl_3$, 298 K): δ = 7.14 (s, 1 H, NH), 6.92 (s, 1H, NH), 1.49 (s, 6 H, 2 × CH_3), 1.47 (s, 6 H, 2 × CH_3), 1.46 (s, 6 H, 2 × CH_3), 1.39 (s, 9 H, 3 × CH_3) ppm; ^{13}**C NMR** (100 MHz, $CDCl_3$, 298 K): δ = 174.1 (CO), 172.9 (CO), 171.8 (CO), 81.8 (C), 64.4 (C), 57.1 (C), 57.0 (C), 27.8 (CH_3), 24.9 (CH_3), 24.3 (CH_3), 24.0 (CH_3) ppm. Spectroscopic data is consistent with the reported data in the literature.[1]

3.7 $NH_2Aib_3O^tBu$

$N_3Aib_3O^tBu$ (0.50 g, 1.45 mmol) was hydrogenated following procedure 1 and the product was isolated as a white solid (0.42 g, 1.27 mmol, 88 %). ^{1}**H NMR** (400 MHz, $CDCl_3$, 298 K): δ = 8.10 (s, 1 H, NH), 7.40 (s, 1 H, NH), 1.45 (s, 6H, 2 × CH_3), 1.42 (s, 6 H, 2 × CH_3), 1.37 (s, 9H, 3 × CH_3), 1.28 (s, 6 H, 2 × CH_3) ppm; ^{13}**C NMR** (100 MHz, $CDCl_3$, 298 K): δ = 177.7 (CO), 174.0 (CO), 173.4 (CO), 81.2 (C), 56.9 (C), 56.5 (C), 54.9 (C), 28.9 (CH_3), 27.8 (CH_3), 25.1 (CH_3), 24.2 (CH_3) ppm. Spectroscopic data is consistent with the reported data in the literature.[1]

3.8 $N_3Aib_4O^tBu$

$NH_2Aib_3O^tBu$ (0.40 g, 1.21 mmol) was coupled following procedure 2 and the product was isolated as a white solid (0.40 g, 0.9 mmol, 75 %). ^{1}**H NMR** (400 MHz, $CDCl_3$, 298 K): δ = 6.97 (s, 1 H, NH), 6.92 (s, 1 H, NH), 6.45 (s, 1 H, NH), 1.50 (s, 12 H, 4 × CH_3), 1.45 (s, 12 H, 4 × CH_3), 1.37 (s, 9 H, 3 × CH_3) ppm; ^{13}**C NMR** (100 MHz, $CDCl_3$, 298 K): δ = 173.1 (CO), 172.6 (CO), 172.5 (CO), 172.3 (CO), 81.0 (C), 63.6 (C), 57.1 (C), 56.9 (C), 56.6 (C), 27.8 (CH_3), 25.1 (CH_3), 25.0 (CH_3), 24.4 (CH_3), 24.3 (CH_3) ppm. Spectroscopic data is consistent with the reported data in the literature.[1]

3.9 $NH_2Aib_4O^tBu$

$N_3Aib_4O^tBu$ (0.25 g, 0.58 mmol) was hydrogenated following procedure 1 and the product was isolated as a white solid (0.18 g, 0.43 mmol, 74 %) ^{1}**H NMR** (400 MHz, $CDCl_3$, 298 K): δ = 8.05 (s, 1 H, NH), 7.20 (s, 1 H, NH), 6.54 (s, 1 H, NH), 1.43 (s, 6H, 2 × CH_3), 1.41 (s, 6 H, 2 × CH_3), 1.37 (s, 9 H, 3 × CH_3), 1.30 (s, 6 H, 2 × CH_3) ppm; ^{13}**C NMR** (100 MHz, $CDCl_3$, 298 K): δ = 178.0 (CO), 173.9 (CO), 173.2 (CO), 172.8 (CO), 80.5 (C), 56.8 (C), 56.6 (C), 56.4 (C), 54.8 (C), 28.9 (CH_3), 27.8 (CH_3), 25.4 (CH_3), 25.0 (CH_3), 24.4 (CH_3) ppm. Spectroscopic data is consistent with the reported data in the literature.[1]

3.10 N_3AibOH

$N_3Aib_4O^tBu$ (0.10 g, 0.23 mmol) was deprotected following procedure 3 and the product was isolated as a white solid (0.075 g, 0.2 mmol, 87 %). ^{1}**H NMR** (400 MHz, $CDCl_3$, 298 K): δ = 7.31 (s, 1H, NH), 6.81 (s, 1H, NH), 6.08 (s, 1H, NH), 1.50 (s, 6 H, 2 × CH_3), 1.45 (s, 6 H, 2 × CH_3), 1.41 (s, 6 H, 2 × CH_3), 1.39 (s, 6 H, 2 × CH_3) ppm; ^{13}**C NMR** (100 MHz, $CDCl_3$, 298 K): δ = 176.9 (CO), 174.6 (CO), 173.6 (CO), 173.0 (CO), 63.9 (C), 57.3 (C), 57.0 (C), 56.9 (C), 25.1 (CH3), 25.0 (CH3), 24.8 (CH3), 24.2 (CH3) ppm. Spectroscopic data is consistent with the reported data in the literature.[1]

3.11 $NH_2AibO(CH_2)_2TMS$

N_3AibOH (0.65 g, 5 mmol) and 2-(trimethylsilyl)ethanol (0.86 mL, 6 mmol) were dissolved in DCM (25 mL). To the resulting clear solution EDC·HCl (1.25 g, 6.5 mmol) and DMAP (60 mg, 0.5 mmol) were added successively The reaction mixture was stirred at room temperature for 18 h under a nitrogen atmosphere. The clear solution was then diluted with DCM (50 mL) and the organic layer was successively washed with 5% $KHSO_4$ (2 × 20 mL), sat. $NaHCO_3$ (2 × 20 mL) and brine (1 × 10 mL). The organic phase was dried over $MgSO_4$, filtrated and the solvent removed. The crude was dissolved in MeOH (5 mL) and Pd/C (50 mg) was added. The mixture was stirred under hydrogen atmosphere for 24 h and was then filtered on Celite® (eluent EtOAc). The solvent was removed and the resulting residue was dissolved in Et_2O (5 mL) and 2 M HCl in Et_2O (3 mL) was added slowly. The mixture was stirred at room temperature for 5 min and the

solvent was removed and hexane was added. The white crystalline product slowly precipitated out. Filtrating and washing the compound with hexane yielded in a white solid (0.55 g, 2.3 mmol, 46%). **^{1}H NMR** (400 MHz, $CDCl_3$, 298 K): δ = 4.16 - 4.24 (m, 2 H, CH_2CH_2TMS), 1.40 (s, 6 H, 2 × CH_3), 0.93 - 1.03 (m, 2 H, CH_2TMS), 0.00 (s, 9 H, $Si(CH_3)_3$) ppm; **^{13}C NMR** (100 MHz, $CDCl_3$, 298 K): δ = 173.2 (CO), 66.8 (CH2), 58.9 (C), 25.5 (CH_3), 18.9 (CH_2), 0.00 (3 × CH_3) ppm; **IR** $ν_{max}$: 3431, 2953, 1746, 1523, 1248, 1193, 1173, 933, 834, 695, 510 $cm^{−1}$; **MS** (ES^+, MeCN): *m/z* = 204 ($[M+Cl]^+$, 80%). Spectroscopic data is consistent with the reported data in the literature.[2]

3.12 $N_3Aib_2O(CH_2)_2$TMS

HCl·H_2NAibO$(CH_2)_2$TMS (135 mg, 0.56 mmol) was coupled to N_3AibOH following procedure 2 and the product was isolated as a pale yellow liquid (130 mg, 0.41 mmol, 73 %). **^{1}H NMR** (400 MHz, $CDCl_3$, 298 K): δ = 6.99 (s, 1 H, NH), 4.15 – 4.19 (m, 2H, CH_2CH_2TMS), 1.50 (s, 6 H, CH_3), 1.47 (s, 6 H, CH_3), 0.90 - 0.99 (m, 2 H,CH_2TMS),0.03 (s, 9 H, $Si(CH_3)_3$) ppm; **^{13}C NMR** (100 MHz, $CDCl_3$, 298 K): δ = 174.7 (CO), 170.9 (CO), 64.3 (C), 64.0 (CH_2), 56.4 (C), 24.5 (CH_3), 24.3 (CH_3), 17.3 (CH_2), −1.5 (3 × CH_3) ppm. Spectroscopic data is consistent with the reported data in the literature.[2]

3.13 $NH_2Aib_2O(CH_2)_2$TMS

$N_3Aib_2O(CH_2)_2$TMS (130 mg, 0.41 mmol) was hydrogenated following procedure 1 and the product was isolated as a white solid (115 mg, 0.40 mmol, 98 %). **^{1}H NMR** (400 MHz, $CDCl_3$, 298 K): δ = 8.07 (s, 1H, NH), 4.18 - 4.25 (m, 2H, CH_2CH_2TMS), 1.54 (s, 6H, 2 × CH_3), 1.35 (s, 6H, 2 × CH_3), 0.98 - 1.03 (m, 2H, CH_2TMS), 0.04 (m, 9H, $Si(CH_3)_3$) ppm.

3.14 $N_3Aib_3O(CH_2)_2$TMS

NH_2Aib_2TMS (115 mg, 0.40 mmol,) was coupled following procedure 2 and the product was isolated as a white solid (135 g, 0.34 mmol, 85 %). **^{1}H NMR** (400 MHz, $CDCl_3$, 298 K): δ = 7.14 (s, 1H, NH), 7.04 (s, 1H, NH), 4.17 - 4.28 (m, 2H, CH_2CH_2TMS), 1.56 (s, 6H, 2 × CH_3), 1.56 (s, 6H, 2 × CH_3), 1.54 (s, 6H, 2 × CH_3), 0.98 - 1.03 (m, 2H, CH_2TMS), 0.05 (s, 9H, $Si(CH_3)_3$) ppm. Spectroscopic data is consistent with the reported data in the literature.[2]

3.15 $NH_2Aib_3O(CH_2)_2$TMS

N_3Aib_3OTMS (135 g, 0.34 mmol) was hydrogenated following procedure 1 and the product was isolated as a white solid (108 mg, 0.29 mmol, 85 %). **^{1}H NMR** (400 MHz, $CDCl_3$, 298 K): δ = 8.16 (s, 1H, NH), 7.63 (s, 1H, NH), 4.17 - 4.24 (m, 2H, CH_2CH_2TMS), 1.54 (s, 6H, 2 × CH_3), 1.53 (s, 6H, 2 × CH_3), 1.38 (s, 6H, 2 × CH_3), 0.97 - 1.03 (m, 2H, CH_2TMS), 0.05 (m, 9H, $Si(CH_3)_3$) ppm.

3.16 $N_3Aib_4O(CH_2)_2$TMS

HCl·H_2NAibO$(CH_2)_2$TMS (14 mg, 0.05 mmol) was coupled to N_3Aib_3OH following procedure 4 and the product was isolated as a pale yellow solid (30 mg, 0.015 mmol, 30 %). **^{1}H NMR** (400 MHz, $CDCl_3$, 298 K): δ = 7.10 (s, 1H, NH), 7.01 (s, 1H, NH), 6.98 (s, 1H, NH), 4.03-4.27 (m, 2H, CH_2CH_2TMS), 1.52 (s, 6H, 2 × CH_3), 1.51 (s, 6H, 2 × CH_3), 1.50 (s, 6H, 2 × CH_3), 1.48 (s, 6H, 2 × CH_3), 1.47 (s, 6H, 2 × CH_3), 0.90-0.99 (m, 2 H,CH_2TMS), 0.00 (s, 9H, $Si(CH_3)_3$) ppm; **^{13}C NMR** ($CDCl_3$, 100 MHz): δ = 175.9 (2 × CO), 174.3 (CO), 173.1 (CO), 65.8 (C), 65.6 (C),60.6 (CH_2), 58.8 (C), 57.9 (C), 26.4 (CH_3), 26.1 (CH_3), 26.0 (CH_3), 25.8 (CH_3), 25.6 (CH_3), 25.6 (CH_3), 18.7 (CH_2), -0.2 (3 × CH_3) ppm. Spectroscopic data is consistent with the reported data in the literature.[2]

3.17 $NH_2Aib_4O(CH_2)_2$TMS

N_3Aib_4OTMS (0.25 g, 0.58 mmol) was hydrogenated following procedure 1 and the product was isolated as a white solid (0.18 g, 0.43 mmol, 74 %) Spectroscopic data is consistent with the reported data in the literature.[2]

3.18 $N_3Aib_8O^tBu$

N_3Aib_4OH (30 mg, 0.078 mmol) was activated to an azlactone following procedure 4 and coupled to $NH_2Aib_4O^tBu$ following procedure 5. The crude product was purified by flash chromatography (SiO_2/Petrolether/EtOAc (85:15) then Petrolether/EtOAc (50:50)) and isolated as a white solid (32 mg, 0.041 mmol, 53 %). **TLC** SiO_2/Petrolether/EtOAc (50:50) R_f = 0.81; **^{1}H NMR** (400 MHz, $CDCl_3$, 298 K): δ = 7.48 (s, 1 H, NH), 7.45 (s, 1 H, NH), 7.42 (s, 1 H, NH), 7.30 (s, 1 H, NH), 7.28 (s, 1 H, NH), 6.89 (s, 1 H, NH), 6.12 (s, 1 H, NH), 1.49 (s, 6 H, 2 × CH_3), 1.46 (s, 6 H, 2 × CH_3), 1.43 (s, 6 H, 2 × CH_3), 1.42 (s, 6 H, 2 × CH_3), 1.41 (s, 6 H, 2 × CH_3), 1.40 (s, 6 H, 2 × CH_3), 1.39 (s, 6 H, 2 × CH_3), 1.37 (s, 9 H, 3 × CH_3), 1.36 (s, 6 H, 2 × CH_3) ppm; **^{13}C NMR** (100 MHz, $CDCl_3$, 298 K): δ = 175.2 (CO), 174.8 (CO), 174.2 (CO), 174.1 (2 × CO), 173.9 (CO), 173.2 (CO), 173.1 (CO), 79.7 (C), 64.0 (C), 60.4 (C), 56.9 (C), 56.8 (C), 56.7 (C), 56.6 (C), 56.6 (C), 56.0 (C), 27.9 (CH_3), 24.8 (CH_3), 24.3 (CH_3) ppm; **IR** ν_{max}: 3289, 2982, 2982, 2941, 2126, 1727, 1659, 1536, 1466, 1455, 1382, 1362, 1227, 1169, 1143 cm^{-1}; **MS** (ES^+, MeCN): *m/z* = 782 ($[M+H]^+$, 100%), 804 ($[M+Na]^+$, 90%). Spectroscopic data is consistent with the reported data in the literature.[3]

3.19 $N_3Aib_8O(CH_2)_2TMS$

N_3Aib_3OH (115 mg, 0.4 mmol) was activated to an active ester following procedure 4 and $NH_2Aib_5O(CH_2)_2TMS$ (204 mg, 0.4 mmol, synthesized by hydrogenolysis of the corresponding azide following general procedure 6) were dissolved in dry acetonitrile (3 mL) and the mixture was heated at reflux for 2 days. The solvent was removed under reduced pressure and purified by column chromatography (SiO_2, EtOAc/CH_2Cl_2 6:4) provided the title compound (255 mg, 0.31 mmol, 78%) as a white solid. **^{1}H NMR** (400 MHz, $CDCl_3$, 298 K): δ = 7.55 (s, 1H, NH), 7.51 (s, 1H, NH), 7.48 (s, 1H, NH), 7.41 (s, 1H, NH), 7.35 (s, 1H, NH), 6.93 (s, 1H, NH), 6.12 (s, 1H, NH), 4.15 - 4.21 (m, 2H, CH_2CH_2TMS), 1.57 (s, 6H, 2 × CH_3), 1.53 (s, 12H, 4 × CH_3), 1.51 (s, 6H, 2 × CH_3), 1.49 (s, 6H, 2 × CH_3), 1.47 (m, 12H, 4 × CH_3), 1.44 (s, 6H, 2 × CH_3), 0.97 - 1.04 (m, 2H, CH_2TMS), 0.04 (s, 9H, $Si(CH_3)_3$) ppm. **^{13}C NMR** (100 MHz, $CDCl_3$, 298 K) δ 176.7 (CO), 176.6 (CO), 176.2 (CO), 176.0 (CO), 175.4 (CO), 175.2 (CO), 174.6 (CO), 174.5 (CO), 65.5 (C), 64.2 (C), 58.3 (C), 58.3 (C), 58.2 (C), 58.1 (C), 58.1 (C), 58.0 (C), 57.1 (C), 26.4 (CH_3), 25.8 (CH_3), 18.5 (CH_2), 0.0 (3 × CH_3) ppm. Spectroscopic data is consistent with the reported data in the literature.[2]

3.20 *N*,*N*-Bis(pyridin-2-ylmethyl)propargylamine

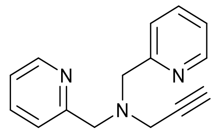

N,*N*-Bis(pyridin-2-ylmethyl)amine (0.1 g, 0.5 mmol) were dissolved in dry THF (2 mL) and K_2CO_3 (70 mg, 0.5 mmol) were added. To the resulting suspension 3-Bromopropyne (0.04 mL, 0.5 mmol) was added. The reaction mixture was stirred at 70 °C for 18 h. The solvent was removed under reduced pressure to give a yellow crude product. The product was further purified using flash chromatography on a silica gel column (eluent: ethyl acetate–ethanol–triethylamine = 15 : 1 : 0.1) to produce a yellow oily liquid (70 mg, 58 %). **^{1}H NMR** (400 MHz, $CDCl_3$, 298 K): δ = 8.59 (d, *J* = 4.7 Hz, 2H, py), 7.70 (td, *J* = 7.6, 1.6 Hz, 2H, py), 7.55 (d, *J* = 7.8 Hz, 2H, py), 7.21–7.17 (m, 2H, py), 3.94 (s, 4H, CH_2), 3.45 (d, *J* = 2.3 Hz, 2H, CH_2), 2.32 (t, *J* = 2.3 Hz, 1H, CH). Spectroscopic data is consistent with the reported data in the literature.[4]

3.21 Aib foldamer 1

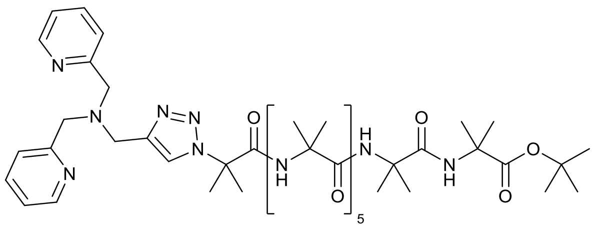

$N_3Aib_8O^tBu$ (20 mg, 0.025 mmol) and *N*,*N*-bis(pyridin-2-ylmethyl)propargylamine (7 mg, 0.03 mmol) were dissolved in EtOH (3mL). Sodium ascorbate (0.5 mL of 20 mM aq. solution) Copper turnings (5 mg) and $CuSO_4$ (0.5 mL of 10 mM aq. solution) was added. The yellow solution was stirred overnight. The resulting dark brown solution was diluted with EtOAc (20 mL) and washed with sat. EDTA-solution (10 mL,pH adjusted to pH = 7 with NaOH). The organic layer was dried over $MgSO_4$, filtered and the solvent removed under reduced pressure. The crude brown solid was purified on a semi-preparative high pressure liquid chromatography (HPLC) purification was performed on HPLC Agilent 1100 series equipped with a semi-preparative C18 column Agilent eclipse XDB-C18, 5 µm, 9.4 mm × 250 mm with a flow rate of 1 mL/min, resulting in a white solid (5 mg, 0.005 mmol, 20%), R_f = 32 min.

HPLC method:

Time [min]	MeCN (%)	Water (%)
0	30	70
10	70	30
20	80	20
30	95	5
70	95	5

^{1}H NMR (400 MHz, $CDCl_3$, 298 K): δ = 8.56 (d, *J* = 4.5 Hz, 2 H, 2 × ArH), 7.90 (s, 1 H,CH (triazole)), 7.74 (td, *J* = 7.6, 1.7 Hz, 2 H, 2 × ArH), 7.61 (s, 2 H, NH), 7.57 (d, *J* = 7.7 Hz, 2 H, 2 × ArH), 7.40 (s, 2 H, NH), 7.38 (br. s, 1H, NH), 7.28 (s, 1 H, NH), 7.25 - 7.23 (3 H, 2 × ArH, NH), 6.92 (br. s., 2 H, 2 × ArH), 3.97 (s, 2 H, CH_2), 3.94 (s, 4 H, 2 × CH_2), 1.89 (s, 6 H, 2 × CH_3), 1.52 (s, 6 H, 2 × CH_3), 1.49 (s, 24 H, 8 × CH_3), 1.46 (s, 6 H, 2 × CH_3), 1.43 - 1.45 (m, 15 H, 5 × CH3) ppm; **^{13}C NMR** (100 MHz, $CDCl_3$, 298 K): δ = 175.5 (CO), 175.1 (CO), 174.8 (CO), 174.3 (CO), 174.2 (CO) 173.9 (CO), 171.57 (CO), 171.1 (CO), 158.63 (2 × C), 148.4 (2 × CH), 144.4 (C, triazole), 137.6 (2 × CH), 124.2 (2 × CH), 122.9 (2 × CH), 122.6 (CH, triazole), 79.8 (C), 65.1 (C), 59.0 (CH_2), 58.7 (C), 56.82 (C), 56.79 (C), 56.7 (C), 56.6 (C), 56.0 (C), 48.5 (2 × CH_2), 27.9 (3 × CH_3), 26.2 (2 × CH_3) , 24.8 (12 × CH_3), 24.6 (2 × CH_3) ppm. **MS** (ES+, MeOH): *m/z* = 1041 ($[M + Na]^+$, 100%), 1057 ($[M + K]^+$, 35%). **HRMS** (ESI): m/z calcd. for $[C_{51}H_{79}O_9N_{13} + H]^+$: 1018.6196, found: 1018.6179.

3.22 Aib foldamer 2

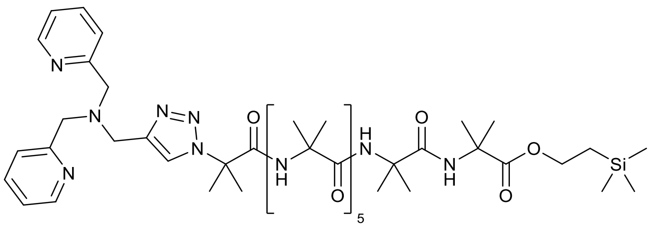

N_3Aib_8OTMS (20 mg, 0.023 mmol) and *N,N*-bis(pyridin-2-ylmethyl)propargylamine (7 mg, 0.03 mmol) were dissolved in EtOH (3 mL). Sodium ascorbate (0.5 mL of 20 mM aq. solution), copper turnings (5 mg) and $CuSO_4$ (0.5 mL of 10 mM aq. solution) was added. The yellow solution was stirred overnight. The resulting dark brown solution was diluted with EtOAc (20 mL) and washed with sat. EDTA-solution (10 mL,pH adjusted to pH = 7 with NaOH). The organic layer was dried over $MgSO_4$, filtered and the solvent removed under reduced pressure. The crude brown solid was purified via HPLC (as for **1** previously) resulting in a white solid (2.5 mg, 0.005 mmol, 22%), R_f = 38 min. **^{1}H NMR** (400 MHz, $CDCl_3$, 298 K): δ = 8.55 (d, *J* = 4.3 Hz, 2 H, 2 × ArH), 7.88 (s, 1 H, CH (triazole)), 7.70 (td, *J* = 7.6, 1.7 Hz, 2 H, 2 × ArH), 7.60 (d, *J* = 5.0 Hz, 2 H, 2 × ArH), 7.54 (s, 1 H, NH), 7.52 (s, 1 H, NH), 7.43 (s, 1 H, NH), 7.36 (s, 2 H, 2 × NH), 7.18 - 7.23 (m, 3 H, 2 × ArH, NH), 6.72 (s, 1 H, NH), 4.13 - 4.20 (m, 2 H, CH_2CH_2TMS), 3.95 (s, 2 H, CH_2), 3.88 (s, 4 H, 2 × CH_2), 1.89 (s, 6 H, 2 × CH_3), 1.52 (s, 12 H, 4 × CH_3), 1.49 (s, 18 H, 6 × CH_3), 1.45 (s, 6 H, 2 × CH_3), 1.44 (s, 6 H, 2 × CH_3), 0.95 - 1.02 (m, 2 H, CH_2TMS), 0.02 (s, 9 H, $Si(CH_3)_3$). **^{13}C NMR** (100 MHz, $CDCl_3$, 298 K): δ = 175.4 (CO), 175.2 (CO), 175.0 (CO), 174.6 (CO), 174.6 (CO), 174.2 (CO), 173.6 (CO), 171.5 (CO), 158.7 (2 × C), 149.1 (CH), 144.6 (C, triazole), 136.7 (CH), 123.6 (CH), 122.4 (CH), 122.1 (CH, triazole), 65.0 (C), 62.8 (**C**H_2CH_2TMS), 59.5 (CH_2), 57.5 (C), 56.9 (C), 56.8 (C), 56.7 (C), 56.6 (C), 55.6 (C), 48.5 (2 × CH_2) 30.3 (C), 26.25 (2 × CH_3), 24.6-26.7 (16 × CH_3), 17.1 (CH_2), −1.5 (3 × CH_3). **MS** (ES+, MeCN): *m/z* = 1063 ($[M + H]^+$, 100%). **HRMS** (ESI): m/z calcd. for $[C_{52}H_{83}O_9N_{13}Si_1 + H]^+$: 1062.6284, found: 1062.6211.

3.23 Aib foldamer 7

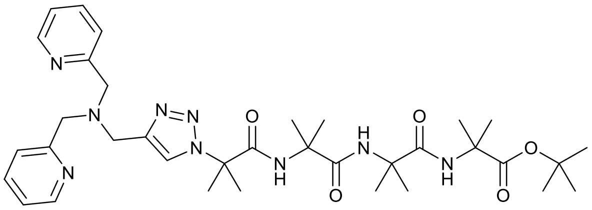

Two methods were used to synthesise this compound:

Method 1: $N_3Aib_4O^tBu$ (10 mg, 0.023 mmol) and *N,N*-bis(pyridin-2-ylmethyl)propargylamine (4 mg, 0.017 mmol) were dissolved in EtOH (1.5 mL). Sodium ascorbate (0.25 mL of 20 mM aq. solution), copper turnings (2.5 mg) and $CuSO_4$ (0.25 mL of 10 mM aq. solution) was added. The yellow solution was stirred overnight. The resulting dark brown solution was diluted with EtOAc (10 mL) and washed with sat. EDTA solution (10 mL, pH adjusted to pH = 7 with NaOH). The organic layer was dried over $MgSO_4$, filtered and the solvent removed under reduced pressure. The crude brown solid was purified via HPLC (as for **1** previously) resulting in a white solid (2 mg, 0.003 mmol, 13%).

Method 2: *N*-Propargyl-di(2-picolyl)amine (28.5 mg, 0.12 mmol), $N_3Aib_4O^tBu$ (44 mg, 0.1 mmol) and copper acetate (3 mg, 0.04 mmol) were dissolved in dry DMF (1 mL) in a round bottom flask, and the solution was heated to 80°C for 1 h under an argon atmosphere. The DMF was co-evaporated with toluene (3 ×) under reduced pressure and the resulting solid was purified by column chromatography on alumina (10% MeOH in CH_2Cl_2) to give a brown solid. This brown solid was purified by semi-preparative high performance liquid

chromatography (HPLC) on an Agilent 1100 series HPLC equipped with a semi-preparative C18 column Agilent eclipse XDB-C18, 5 µm, 9.4 mm × 250 mm with a flow rate of 1 mL/min. The product containing fractions were combined and the organic solvent was removed under reduced pressure. The aqueous solution was freeze-dried to give the product as a white solid (32 mg, 0.47 mmol, 47%).

Time [min]	MeCN (%)	Water (%)
0	30	70
10	70	30
20	80	20
30	95	5
50	95	5

^{1}H NMR (400 MHz, $CDCl_3$, 298 K): δ = 8.55 (d, *J* = 4.3 Hz, 2 H, 2 × ArH), 7.87 (s, 1 H, CH (triazole)), 7.68 (td, *J* = 7.6, 1.7 Hz, 2 H, 2 × ArH), 7.56 (d, *J* = 7.8 Hz, 2 H, 2 × ArH), 7.16 - 7.21 (td, 2 H, 2 × ArH), 6.98 (s, 1 H, NH), 6.64 (s, 1 H, NH), 6.44 (s, 1 H, NH), 3.94 (s, 2 H, CH_2), 3.88 (s, 4 H, 2 × CH_2), 1.90 (s, 6 H, 2 × CH_3), 1.49 (s, 6 H, 2 × CH_3), 1.44 (s, 9 H, 3 × CH_3), 1.40 (s, 6 H, 2 × CH_3) ppm; **^{13}C NMR** (100 MHz, $CDCl_3$, 298 K): δ = 173.7 (CO), 173.1 (CO), 172.1 (CO), 171.1 (CO), 158.8 (2 × C), 149.1 (2 × CH), 146.6 (C, triazole), 136.6 (2 × CH), 123.5 (2 × CH), 122.3 (2 × CH), 122.0 (CH, triazole), 80.6 (C), 65.3 (C), 59.6 (CH_2), 57.5 (C), 56.9 (C), 56.4 (C), 48.6 (2 × CH_2), 27.8 (3 × CH_3), 26.1 (2 × CH_3), 25.2 (2 × CH_3), 24.9 (2 × CH_3), 24.5 (2 × CH_3) ppm. **MS** (ES+, MeOH): *m/z* = 701 ([M+Na]+, 100%), 717 ([M+K]+, 60%). **HRMS** (ESI): m/z calcd. for $[C_{35}H_{51}O_5N_9 + K]^+$: 716.3645, found: 716.3636.

3.24 Conditions for complexation of CuCl with foldamer 1

Foldamer **1** (6 mg, 6 µmol) was dissolved in MeOH (3 mL). Afterwards a CuCl solution in MeOH (50 mL, 0.1 mM) was added over 1 h. The resulting solution was stirred for 1 h and the solvent was reduced to 5 mL. The resulting solution was stored in a sealed jar containing a small amount of diethylether. After three days blue crystals were formed. The crystals were stored in solution to try and prevent oxidation. Before use the crystals were filtered and dried in a ventilated oven at 60 °C to obtain 2 mg (2 µmol, 30%) of blue crystals. **MS** (ES+, MeCN): *m/z* = 1082 ([Cu(**1**) + 2H]$^+$, 100%).

3.25 Conditions for complexation of CuCl with foldamer 2

Foldamer **2** (6 mg, 6 µmol) was dissolved in MeOH (3 mL). Afterwards a CuCl solution in MeOH (50 mL, 0.1 mM) was added over 1 h. The resulting solution was stirred for 1 h and the solvent was reduced to 5 mL. The resulting solution was stored in a sealed jar containing a small amount of diethyl ether. After three days blue crystals were formed. The crystals were stored in solution to try and prevent oxidation. Before use the crystals were filtered and dried in a ventilated oven at 60°C to obtain 2 mg (2 µmol, 30%) of blue crystals. **MS** (ES+, MeCN): *m/z* = 1125 ([Cu(**2**)]$^+$, 5%), 2321 ([Cu_2(**2**)$_2Cl_2$ + 2H]$^+$, <5%). **HRMS** (ESI): m/z calcd for $[C_{52}H_{83}N_{13}O_9Si_1Cu]^+$ = 1124.5502, found: 1124.5504.

3.26 Conditions for complexation of CuCl with foldamer 7

Foldamer **7** (4 mg, 6 µmol) was dissolved in degassed MeOH (3 mL) under an argon atmosphere. Then a suspension of CuCl in MeOH (0.1 mM, 50 mL) was added over 1 h. The resulting pale blue suspension was stirred for 1 h before the solvent volume was reduced under reduced pressure to 0.5 mL. To this solution was added $CHCl_3$ (2 mL). The resulting pale blue solution was placed within a vial inside in a sealed jar containing diethyl ether. A blue solid formed after one day, which was stored *in situ*. Before use, the solution was removed from the solid by vacuum filtration and the resulting solid was dried at room temperature under vacuum. The product was obtained as a blue solid (2 mg, 2.6 µmol, 43%). **MS** (ES−, MeCN): m/z = 847.5 ($[Cu(\mathbf{7})Cl_3]^-$, <5%). (ES+, MeCN): m/z = 775.5 ($[Cu(\mathbf{7})Cl]^+$, 5%), 785.5 ($[Cu(\mathbf{7})(formate)]^+$, 2%), . **HRMS** (ESI): m/z calcd for $[Cu(\mathbf{7})Cl]^+$, $[C_{35}H_{51}N_9O_5CuCl]^+$ = 775.2992, found: 775.2955. m/z calcd for $[Cu_2(\mathbf{7})_2ClOH]^{2+}$ $[C_{70}H_{103}N_{18}O_{11}Cu_2Cl]^{2+}$ = 766.3162, found: 766.3298. **UV-Visible:** λ_{max} = 292, 658 nm (MeOH), λ_{max} = 765 nm ($CHCl_3$).

3.27 ¹H NMR spectrum of product from complexation of CuCl with foldamer 2

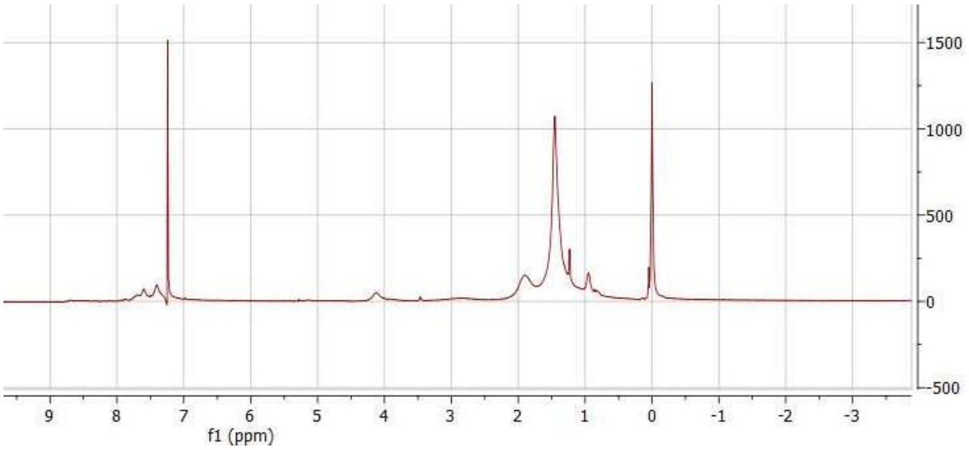

Figure S1: ^{1}H NMR spectrum of the product from complexation of CuCl with foldamer **2** showing broadening, proposed to be due to aerial oxidation to Cu(II).

3.28 Discolouration of product from complexation of CuCl with foldamer 7

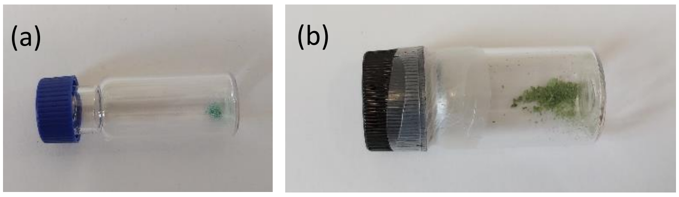

Figure S2: Images of product from complexation of CuCl with foldamer **7** (a) soon after synthesis and (b) 2 h after synthesis and drying.

3.29 Complex Cu(II)[1]Cl_2

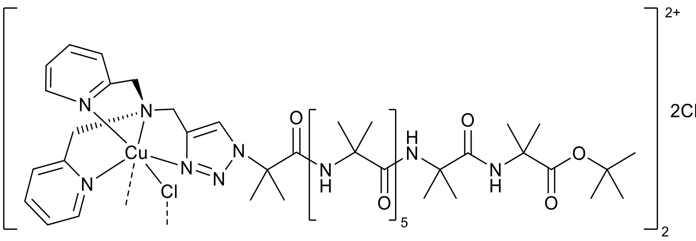

Foldamer **1** (10 mg, 10 µmol) was dissolved in MeOH (3 mL). Afterwards a $CuCl_2$ solution in MeOH (5 mL, 2 mM) was added. The resulting solution was stirred for 10 min and the resulting solution was stored in a sealed jar containing a small amount of diethyl ether. After two days a green solid was formed. Diethylether (4 mL) was carefully added on top of the MeOH solution and left for one more day to mix. The solid was filtered off and air dried for two days and afterwards further dried in a ventilated oven at 60 °C to obtain 8 mg (7 µmol, 70 %) of a green solid. **MS** (ES+, MeCN): *m/z* = 1116 ([M − Cl]$^+$, 50%); For monomer: **HRMS** (ESI): m/z calcd for [$C_{51}H_{79}N_8O_5Cu_1Cl_2$ – Cl]$^-$ = 1115.5108, found: 1115.5213.

3.30 Complex Cu(II)[2]Cl_2

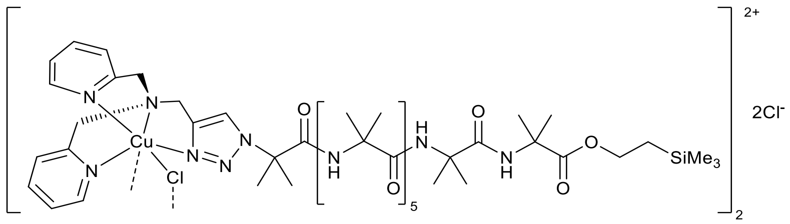

Foldamer **2** (10 mg, 10 µmol) was dissolved in MeOH (3 mL). Afterwards a $CuCl_2$ solution in MeOH (5 mL, 2 mM) was added. The resulting solution was stirred for 10 min and the resulting solution was stored in a sealed jar containing a small amount of diethyl ether. After two days green crystals were formed. Diethyl ether (4 mL) was carefully added on top of the MeOH solution and left for one more day to mix. The solid was filtered off and air dried for two days and afterwards further dried in a ventilated oven at 60 °C to obtain 8 mg (7 µmol, 70%) of green crystals. **MS** (ES$^+$, MeCN): *m/z* = 1160 ([M – Cl]$^-$, 65%); For monomer: **HRMS** (ESI): m/z calcd for [$C_{52}H_{83}N_{13}O_9Si_1Cu_1Cl_2$ – Cl]$^-$ = 1159.5191, found: 1159.5332. For dimer: **HRMS** (ESI): m/z calcd for [$C_{104}H_{166}N_{26}O_{18}Si_2Cu_2Cl_4$ – Cl]$^-$ = 2355.0103, found: 2354.9918. Elemental analysis for $C_{104}H_{166}N_{26}O_{18}Si_2Cu_2Cl_4$ + $4H_2O$ + $0.5CH_2Cl_2$ + $2CH_3CN$: expected: C, 50.31; H, 7.04; N, 15.14; Cl, 6.84; Cu, 4.91; found: C: 50.41, H: 7.14, N: 15.33, Cl: 6.35, Cu: 5.23.

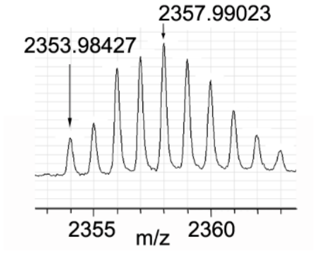

Figure S3: Mass spectroscopic data of [Cu(II)[**2**](µ-Cl)]$_2$Cl$_2$. showing the molecular ion for the partially reduced dimer [Cu(II)$_2$[**2**]$_2$Cl$_2$]$_2$$^+$ at monoisotopic m/z 2353.98427.

3.31 Complex Cu(II)[7]Cl$_2$

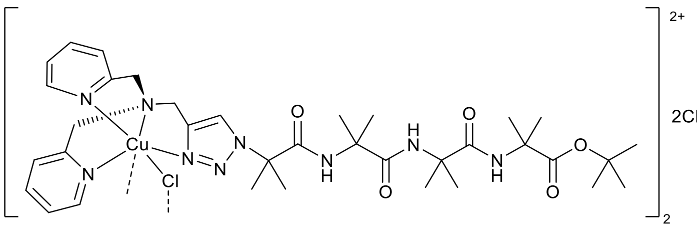

Foldamer **7** (4 mg, 6 µmol) was dissolved in MeOH (3 mL), then a $CuCl_2$ solution in MeOH (2 mM, 2.5 mL) was added. The resulting blue solution was stirred for 10 min before the solvent was removed under reduced pressure. The resulting green oil was dissolved in $CHCl_3$ (5 mL) and the green solution was placed within a vial inside in a sealed jar containing diethyl ether. After one day a green solid had formed. Diethyl ether (2 mL) was carefully layered on top of the $CHCl_3$ solution and left for a further day. The solution was removed from the solid by vacuum filtration and the resulting solid was air dried for two days before further drying in a ventilated oven at 60 °C. The product was obtained as a green solid (3.2 mg, 4 µmol, 67%). **MS** (ES+, MeCN): *m/z* = 775 ([M - Cl]$^+$, 5%), **HRMS** (ESI): m/z calcd for [$C_{35}H_{51}N_9O_5Cu_1Cl_2$ – Cl]$^-$ = 775.2992, found: 775.2960. UV-vis: λ_{max} = <200, >800 nm (MeOH), λmax = 259, 305, 475 nm ($CHCl_3$).

4. ^{1}H and ^{13}C NMR spectra of synthesized compounds

4.1.1 Foldamer 1

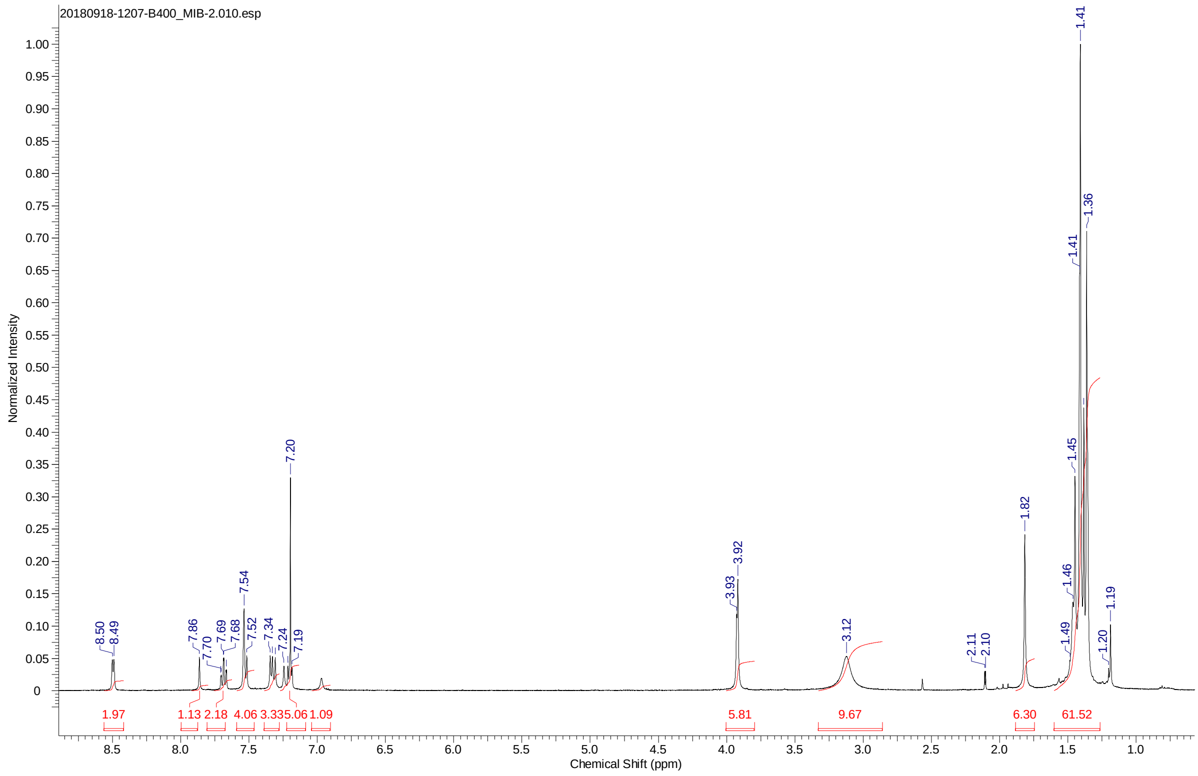

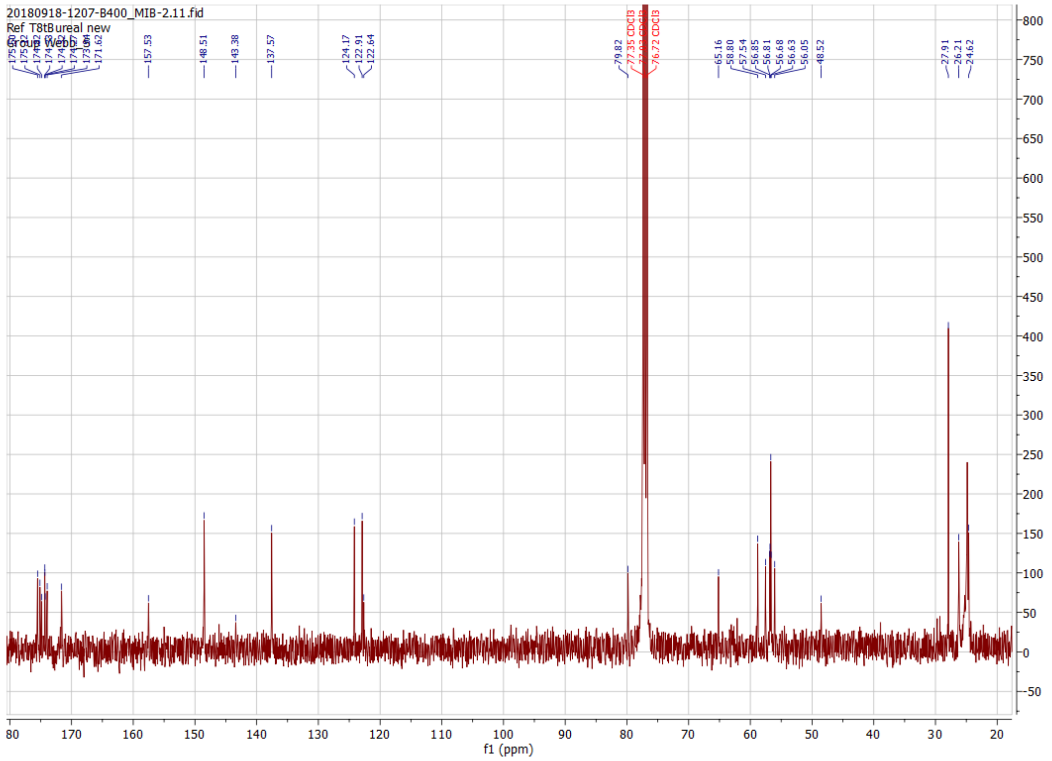

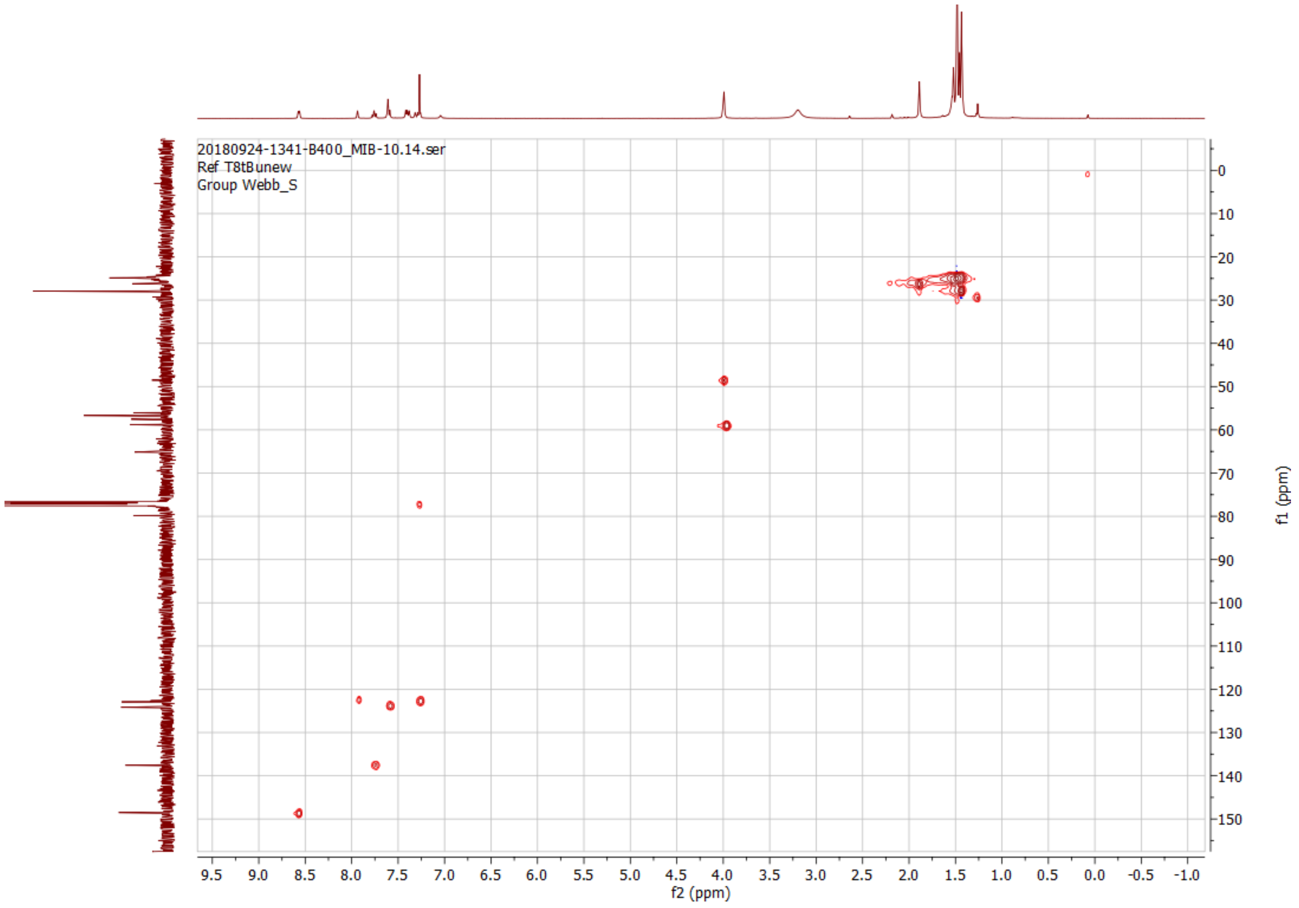
20180924-1341-B400_MIB-10.14.ser
Ref T8tBunew
Group Webb_S
f2 (ppm)
f1 (ppm)

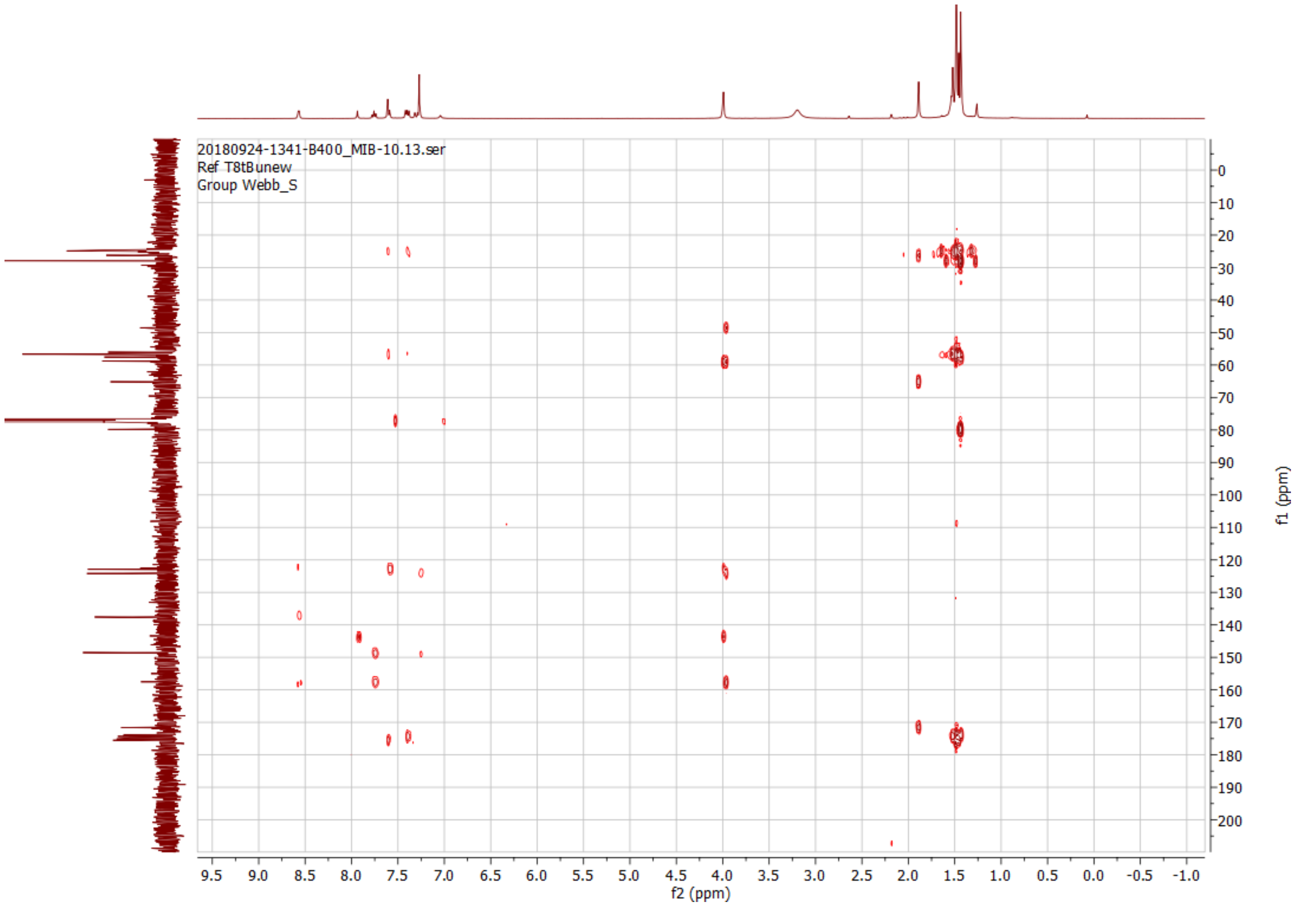
20180924-1341-B400_MIB-10.13.ser
Ref T8tBunew
Group Webb_S
f2 (ppm)
f1 (ppm)

4.1.2 Foldamer 2

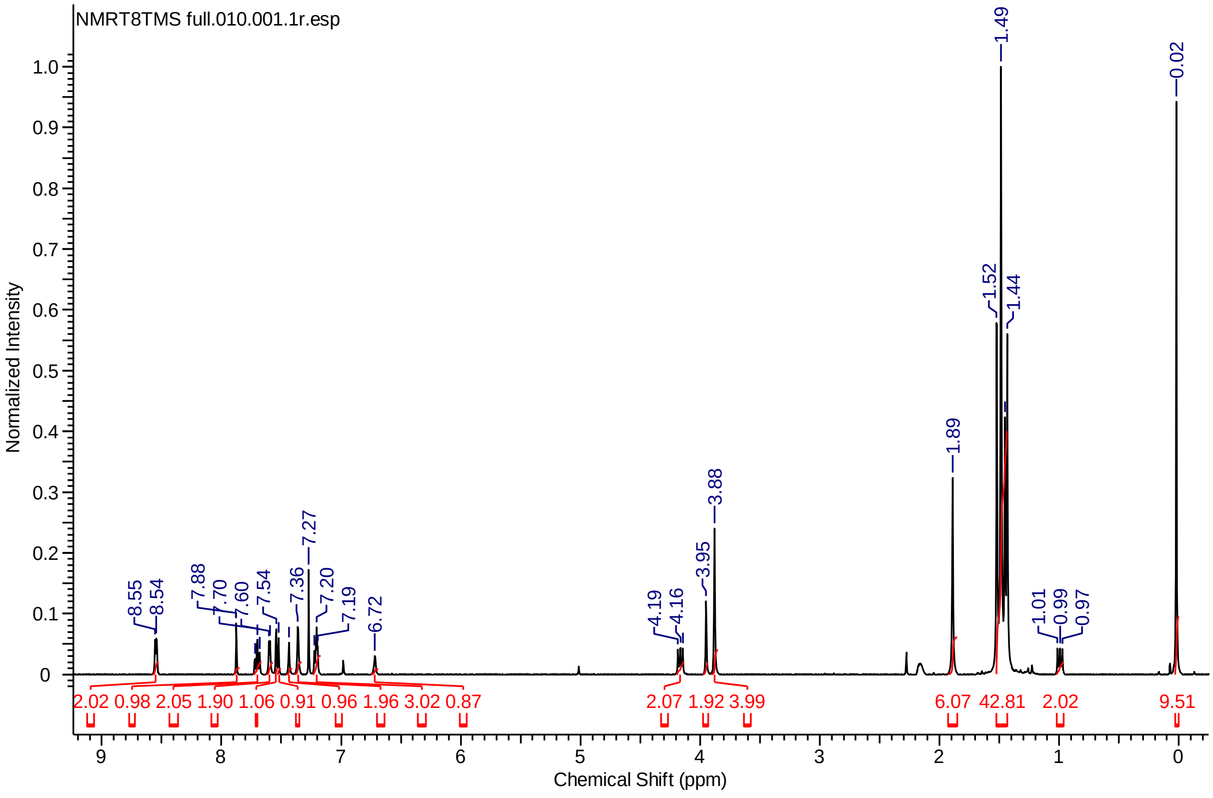

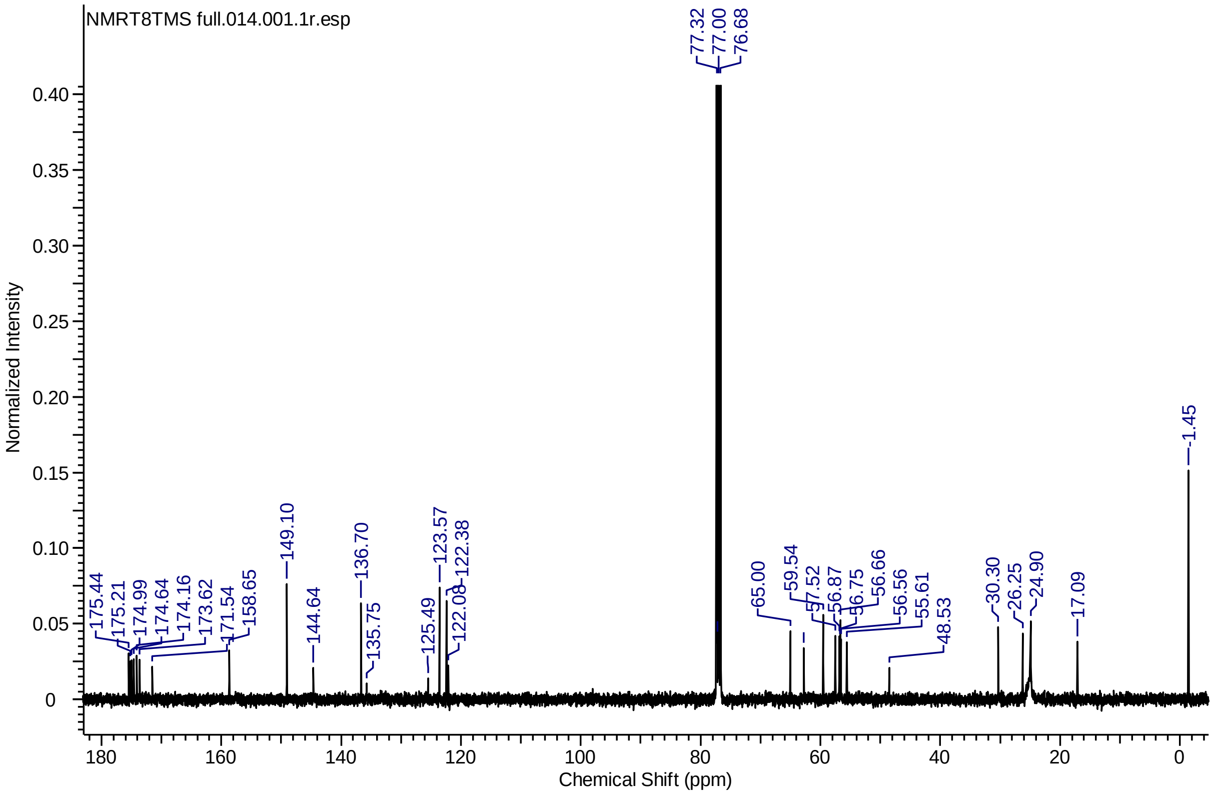

4.1.3 Foldamer 7

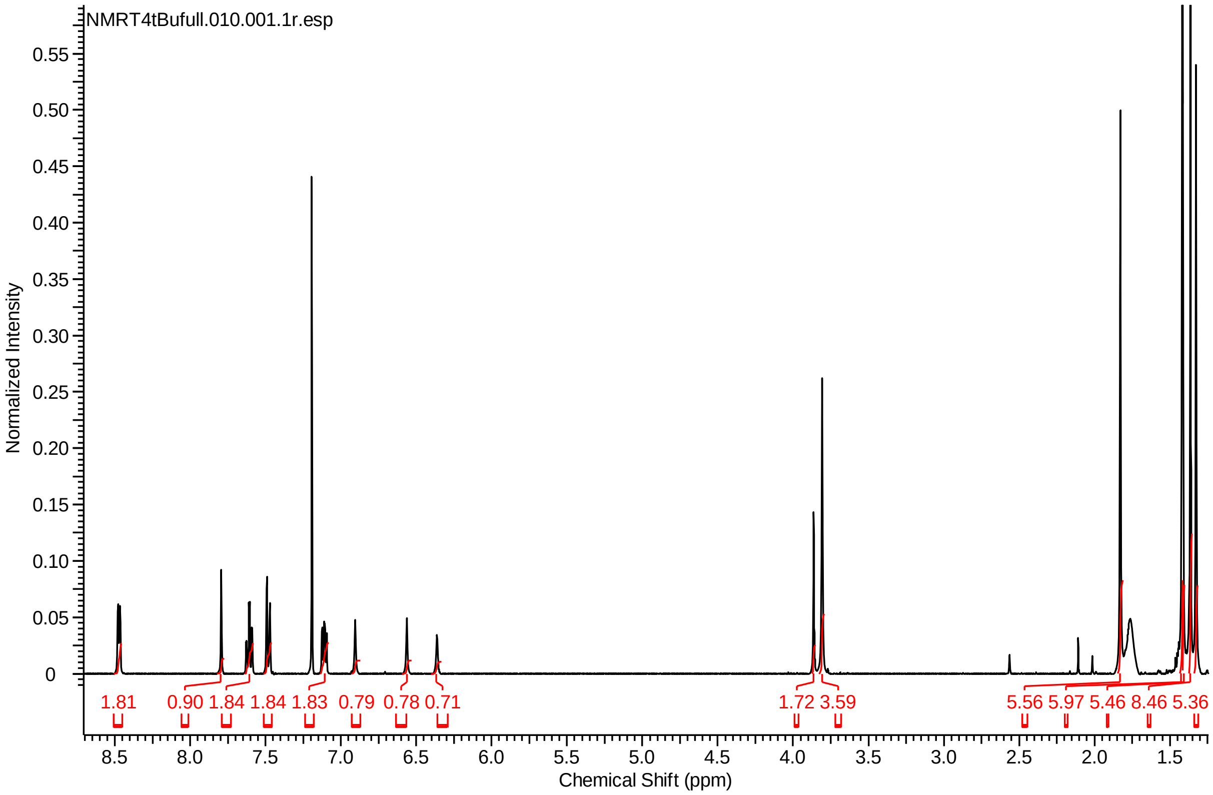

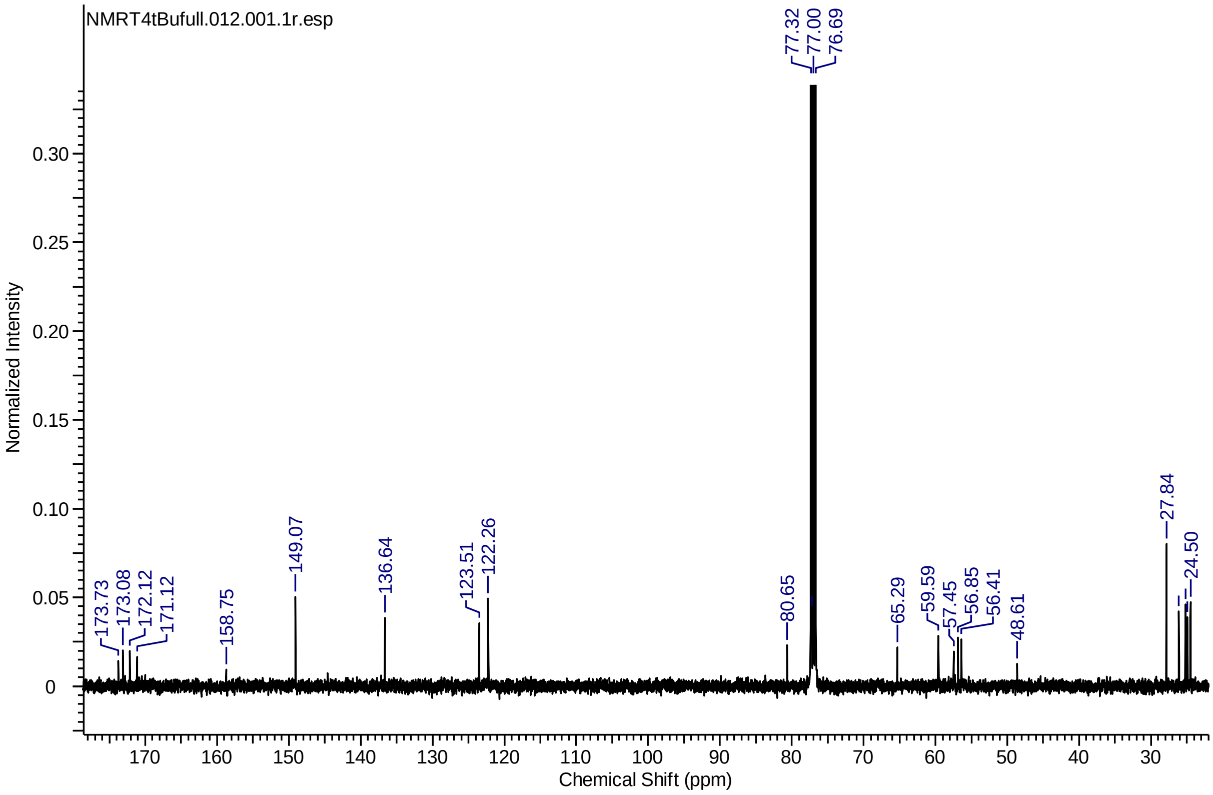

5. Vesicle studies

5.1 Preparation of large unilamellar vesicles for HPTS assays

The HPTS assays of **1**, **2**, Cu[**1**]Cl_2 and Cu[**2**]Cl_2 used 1:4 cholesterol:egg yolk phosphatidylcholine (EYPC) vesicles in MOPS buffer (pH 7.4) with 100 mM of an appropriate salt (e.g. KCl, KBr, NaCl).[5] Aliquots of **1**, **2**, Cu[**1**]Cl_2 or Cu[**2**]Cl_2 dissolved in methanol were mixed with the vesicle suspensions for 60 s before adding a base pulse to change the external pH to 8.4.[6] The resulting change in HPTS fluorescence was followed for 6 min, then Triton X-100 was added at 7 min to lyse the vesicles and allow. Each assay was repeated three times on new vesicles. The resulting normalized data were fitted to pseudo first-order rate equations as an approximation (see the ESI), similar to the procedure of Regen and co-workers.[7] Although the change in fluorescence after the "burst phase" is likely to arise from multiple processes, including inter-vesicle transfer of foldamers,[8] this fitting allows the relative effectiveness of each compound to be compared.

Egg yolk phosphatidylcholine (EYPC, 64 µmol) and cholesterol (16 µmol) were dissolved in 2 mL chloroform (spectroscopic grade). The solvent was removed under reduced pressure resulting in a thin film of lipid on the inside wall of the round bottom flask, which was dried further under vacuum for 2 hours. 1.2 mL of a trisodium 8-hydroxypyrene-1,3,6-trisulfonate (HPTS, 100 mM, pH = 7.4) dye in (3-(*N*-morpholino) propanesulfonic acid) buffer (20 mM MOPS, 100 mM MX (M = K or Na, X = Cl, Br or NO_3^- adjusted to pH 7.4 using NaOH) were added to the lipid film. Afterwards, the thin film was detached from the sides of the flask by vortex mixing. The resulting lipid suspension was then extruded 19 times through an 800 nm polycarbonate membrane in an Avestin Liposofast extruder to give a suspension of 800 nm large unilamellar vesicles. Unencapsulated HPTS dye was removed by gel permeation chromatography on PD-10 SEC columns (Sephadex G-25). Hereby 1 mL of the 800 nm vesicle suspension was diluted to 2.5 mL with 20 mM MOPS buffer solution and loaded onto the GPC column. The suspension run onto the column, followed by 3.5 mL of 20 mM MOPS buffer, which were collected, giving a stock vesicle solution of 3.5 mL (final concentration of lipids = 15.23 mM). This solution of vesicles was used directly in the HPTS experiments.

5.2 Procedure for HPTS assays

100 µL of the vesicle stock solution was diluted to 2 mL with 20 mM MOPS buffer (final lipid concentration in cuvette 0.762 mM). To this vesicle suspension was then added 20 µL of the principle compound in methanol (1 mM or 0.6 mM for Cu[**2**]Cl_2) and the cuvette was equipped with a stirrer bar. The cuvette was then placed in the fluorimeter with fast stirring mode, and the fluorescence emission at 510 nm observed, resulting from the simultaneous excitation at 405 nm and 460 nm over a period of seven minutes (420 seconds) at 25°C. After one minute (60 s) the base pulse was provided by adding 13 µL of a 1M NaOH (aq). At seven minutes (420 s) 40 µL of a 10 % v/v solution of Triton X-100 detergent in MOPS was added to lyse the vesicles. Fluorescence time courses were normalised using the equation

$I_n = (F_t - F_0) / (F_\infty - F_0)$

with $F_0 = F_t$ at addition of base pulse, $F_\infty = F_t$ at saturation after complete leakage.

5.2.1 Procedure for the determination of first order rate constants

The normalised data (I_n) was fitted to first order kinetics using an equation of the general form:

$I_t = I_\infty - [(I_\infty - I_o)exp(-k_{obs}t)]$.

with t in seconds. I_n is the emission intensity at time t, I_∞ is the final normalised fluorescence intensity at t = ∞ and I_o is the estimated initial normalised fluorescence intensity at t = 0 minutes.

The fit was started from t = 0, as this is the point of compound addition.

HPTS assays were repeated three times and showed good experimental reproducibility. Repeated fitting to pseudo-first order kinetics and assessment of the goodness of fit provided an approximation of the errors inherent in the curve fitting process, estimated as ±0.001 s^{-1}.

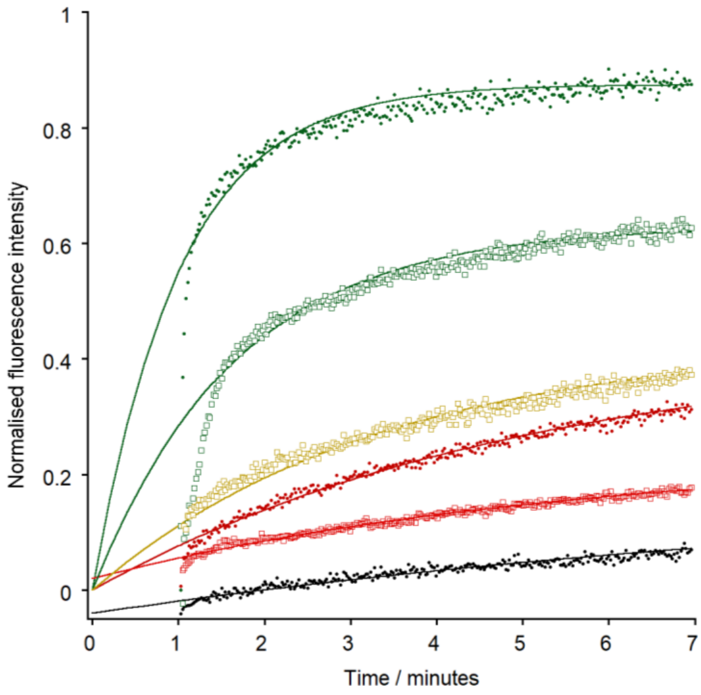

Figure S4: Comparison of the HPTS curves in the presence of 100 mM KCl for: methanol, (●, fit shown for k_{obs} = 0.0016 s^{-1}); Cu(II)[**1**]Cl_2, (□, fit shown for k_{obs} = 0.0100 s^{-1}); Cu(II)[**2**]Cl_2 (●, fit shown for k_{obs} = 0.0165 s^{-1}); Cu(II)[**7**]Cl_2 (□, fit shown for k_{obs} = 0.005 s^{-1}); **1** (□, fit shown for k_{obs} = 0.0030 s^{-1}); **2**, (●, fit shown for k_{obs} = 0.0033 s^{-1}).

5.2.2 $CuCl_2$ gating of ionophoric activity of 1 and 2

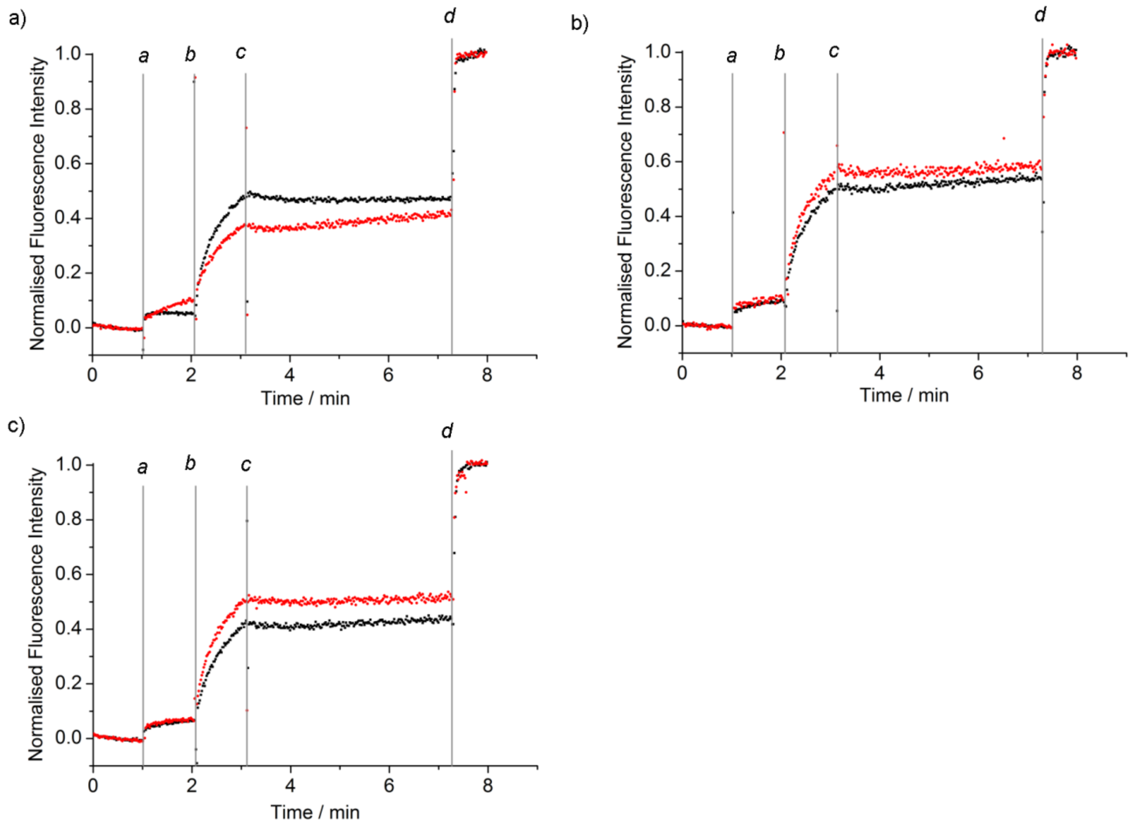

Figure S5: Switching of ionophoric activity for **1** (10 µM, ●) and **2** (6 µM, ●) in the presence of (a) NaCl (100 mM), (b) KCl (100 mM), (c) KBr (100 mM). *a* Base pulse at 1 min; *b* $CuCl_2$ addition (2 eq. at 120 s) switches activity "on"; *c* EDTA addition (2.2 eq. at 180 s) switches activity "off"; *d* TX-100 added at 7 min. The corresponding data for the addition of MeOH (20 µL) has been subtracted from these data.

5.2.3 Effect of $CuCl_2$ addition on the HPTS assay

To confirm that neither the addition of $CuCl_2$ solution, nor the addition of EDTA solution had an ionophoric effect, the "gating" experiment was performed in the same way but in the absence of the active compounds (**1** or **2**).

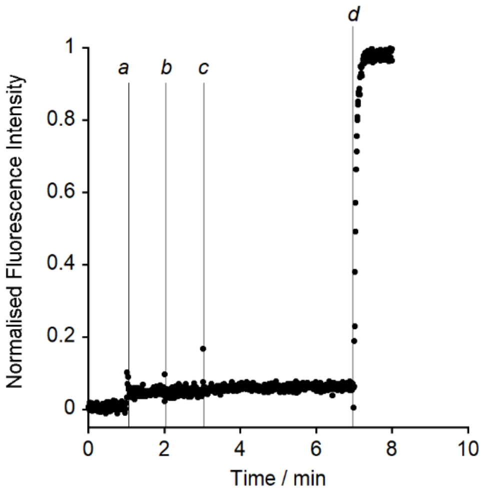

Figure S6: Effect of $CuCl_2$ addition on the HPTS assay in the presence of KCl (100 mM). *a* Base pulse at 1 min; *b* $CuCl_2$ addition (20 µM); *c* EDTA addition (2.2 eq. at 180 s); *d* TX-100 added at 7 min.

5.2.4 HPTS curves for 1 ,2, Cu(II)[1]Cl_2, Cu(II)[2]Cl_2, Cu(II)[7]Cl_2 with different salts

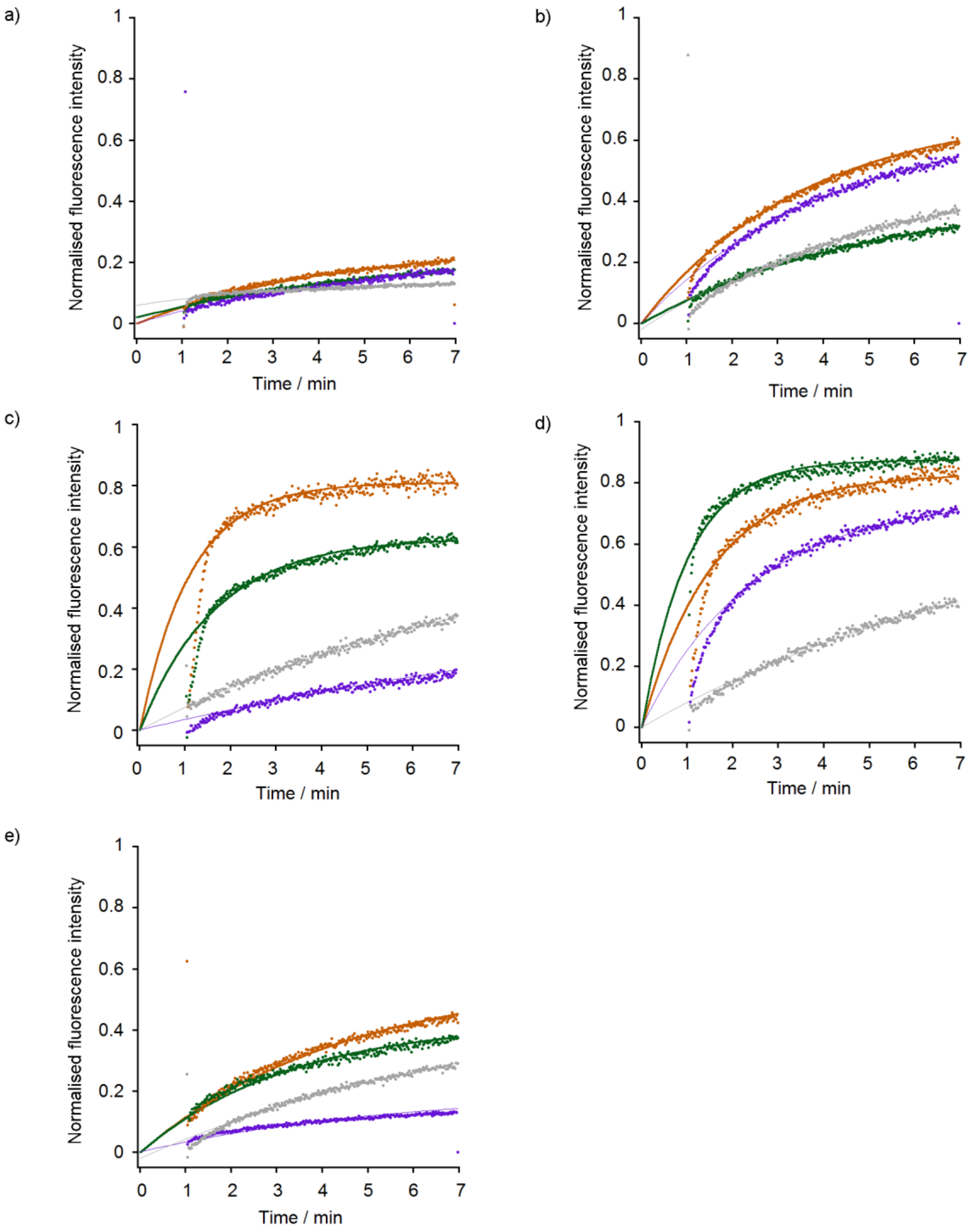

Figure S7: (a-e) HPTS curves for (a) **1** (b) **2**, (c) Cu(II)[**1**]Cl_2, (d) Cu(II)[**2**]Cl_2, (e) Cu(II)[**7**]Cl_2 (all 10 µM except Cu(II)[**2**]Cl_2 6 µM) in the presence of 100 mM of the salts: NaCl (●), KCl (●), KBr (●), KNO_3 (●)

Table S1: Apparent observed rate constants k_{obs} (10^{-3} s^{-1}). Methanol control is 1×10^{-3} s^{-1}.

Salt (100 mM)	Compound (concentration)				
	**1** (10 µM)	**2** (10 µM)	Cu[**7**]Cl_2 (10 µM)	Cu[**1**]Cl_2 (10 µM)	Cu[**2**]Cl_2 (6 µM)
NaCl	4	4.6	3.7	15	10.8
KCl	3	3.3	5.0	10	16.5
KBr	3.3	4.0	3.0	1.2	6.8
KNO_3	4	3.2	2.0	2.0	1.9

5.3 Carbonyl cyanide-*p*-trifluoromethoxyphenylhydrazone (FCCP) data

Analysis of the effect of FCCP on the measurement of ion transport by **1**, **2**, Cu(II)[**1**]Cl_2 and Cu(II)[**2**]Cl_2 in the HTPS assay were performed according to the procedure of Shinde and Talukdar.[9] FCCP was added at 2 minutes, but was only observed to have a significant effect on the membrane activity of the free foldamers **1** and **2**; the corresponding Cu(II)Cl_2 complexes showed little change. Addition of FCCP on its own did not cause a significant change.

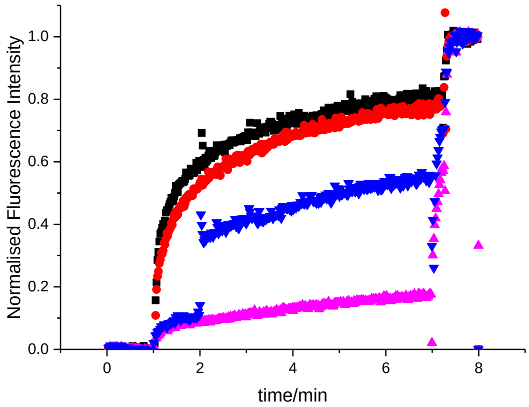

Figure S8: Ionophoric activity determined through an HPTS assay in the presence of NaCl (100 mM) for: **1** (10 µM) in the absence (pink inverted triangles) and presence of FCCP (20 µM, blue triangles) and Cu(II)[**1**]Cl_2 (10 µM) in the absence (red circles) and presence of FCCP (20 µM, black squares). Base pulse at 1 min, TX-100 added at 7 min.

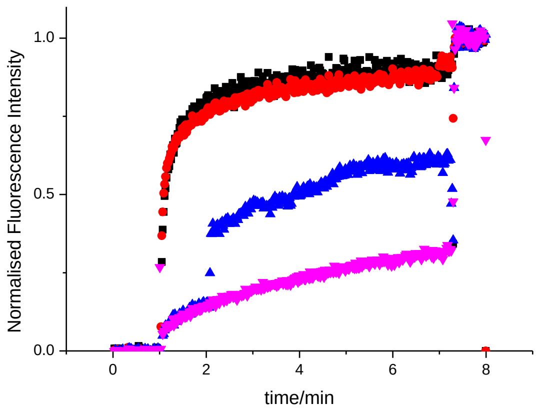

Figure S9: Ionophoric activity determined through an HPTS assay in the presence of NaCl (100 mM) for: **2** (10 µM) in the absence (pink inverted triangles) and presence of FCCP (20 µM, blue triangles) and Cu(II)[**2**]Cl_2 (6 µM) in the absence (red circles) and presence of FCCP (20 µM, black squares). Base pulse at 1 min, TX-100 added at 7 min.

5.4 Preparation of large unilamellar vesicles for lucigenin assays

Egg yolk phosphatidylcholine (EYPC, 64 µmol) and cholesterol (16 µmol) were dissolved in 2 mL chloroform (spectroscopic grade). The solvent was removed under reduced pressure resulting in a thin film of lipid on the inside wall of the round bottom flask, which was dried further under vacuum for 2 hours. 1.2 mL of a 1 mM lucigenin dye in (3-(*N*-morpholino) propanesulfonic acid) buffer (200 mM $NaNO_3$ in water, pH = 7.4) were added to the lipid film. Afterwards, the thin film was detached from the sides of the flask by vortex mixing. The resulting lipid suspension was then extruded 19 times through an 800 nm polycarbonate membrane in an Avestin Liposofast extruder to give a suspension of 800 nm large unilamellar vesicles. Unencapsulated HPTS dye was removed by gel permeation chromatography on PD-10 SEC columns (Sephadex G-25). Hereby 1 mL of the 800 nm vesicle suspension was diluted to 2.5 mL with 20 mM MOPS buffer solution and loaded onto the GPC column. The suspension run onto the column, followed by 3.5 mL of 20 mM MOPS buffer, which were collected, giving a stock vesicle solution of 3.5 mL (final concentration of lipids = 15.23 mM). This solution of vesicles was used directly in the lucigenin experiments.

5.5 Procedure for lucigenin assays

Lucigenin assays procedure and fluorescence normalisation were developed by a modification of published methods.[10] An aliquot of the vesicle stock solution (100 µL) was diluted to 1.9 mL with 200 mM $NaNO_3$ in water (pH = 7.4) (final lipid concentration in cuvette 0.762 mM). To this vesicle suspension was then added 20 µL of the principal compound in methanol (1 mM) and the cuvette was equipped with a stirrer bar. The cuvette was then placed into a Varian Cary Eclipse fluorescence spectrophotometer in the moderate stirring mode. The fluorescence emission at 505 nm was observed, resulting from excitation at 455 nm, over a period of seven minutes (420 seconds) at 25°C. After one minute (60 s), an aliquot of a NaCl solution in water (100 µL, 480 mM) was added. The change in fluorescence was followed for six minutes before final lysis of the vesicles at seven minutes (420 s) by adding an aliquot of a 10 % v/v solution of Triton X-100

detergent in MOPS (40 µL). Triton X-100 lysed the vesicles and quenched all the lucigenin, allowing data normalisation. Data was collected for a further minute (60 s). After normalisation, the data was fitted to first-order kinetics as an approximation (see the ESI).

Fluorescence time courses were normalised using the equation

$I_n = (F_t - F_0) / (F_\infty - F_0)$

with $F_0 = F_t$ at addition of NaCl pulse, $F_\infty = F_t$ at saturation after complete leakage (Triton X-100 addition).

The normalised data (I_n) was fitted to first order kinetics using an equation of the general form:

$I_t = I_\infty + [(I_o - I_\infty)exp(-k_{obs}t)]$.

with t in seconds. I_n is the emission intensity at time t, I_∞ is the final normalised fluorescence intensity at t = ∞ and I_o is the estimated initial normalised fluorescence intensity at t = 0 minutes (and equal to 1).

The fit was started from t = 0, as this is the point of compound addition.

5.6 Curve fitting of lucigenin assays and data for Cu(II)[1]Cl_2

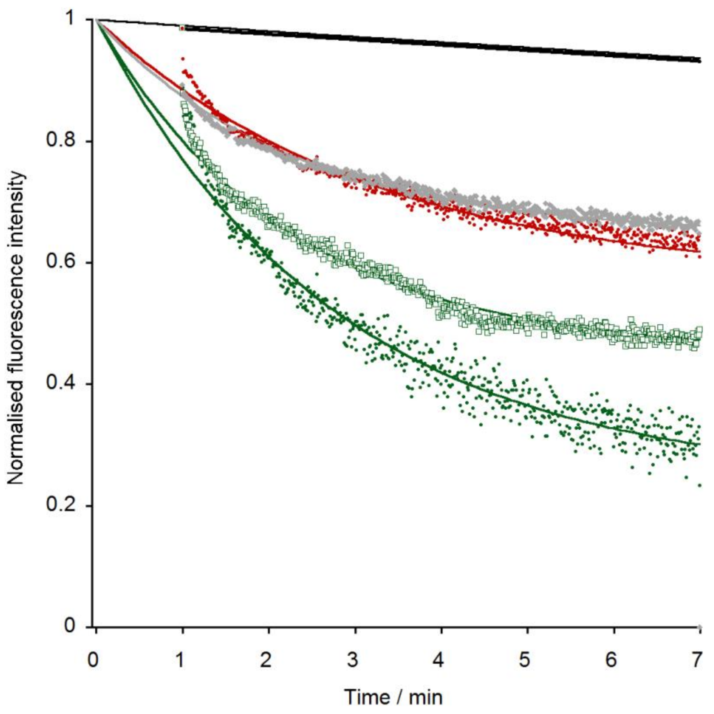

Figure S10: Lucigenin assays of Cl^- transport by MeOH (●, curve fit shown for k_{obs} = 0.00029 s^{-1}), **2** (●,curve fit shown for k_{obs} = 0.0052 s^{-1}), Cu(II)[**1**]Cl_2 (□, curve fit shown for k_{obs} = 0.0075 s^{-1}) and Cu(II)[**2**]Cl_2 (●, curve fit shown for k_{obs} = 0.006 s^{-1}) (all 10 µM) and alamethicin (●, curve fit shown for k_{obs} = 0.0073 s^{-1}) (100 µM) with interior $NaNO_3$ (200 mM $NaNO_3$) and exterior NaCl (2 M). Compounds added at 0 min, NaCl added at 1 min, TX-100 added at 7 min.

5.7 Preparation of large unilamellar vesicles for CF release assays

A 50 mM 5(6)-carboxyfluorescein (CF) solution in MOPS buffer (20 mM MOPS, 100 mM NaCl, pH 7.4) was prepared by dissolving 5/6-CF in MOPS buffer, adding a sodium hydroxide (1M) solution dropwise until pH 11 was reached. The resulting solution was the slowly acidified to pH 7.4.

EYPC-cholesterol (4:1) 800 nm vesicles were prepared as described above, using the above mentioned 5(6)-carboxyfluorescein (CF) (50 mM) solution.

Unencapsulated 5/6-CF was removed by gel permeation chromatography on PD-10 SEC columns (Sephadex G-25) as described for the procedure for LUV preparation.

5.8 U-tube metal picrate transport experiments

The used procedure was developed by modification of published methods.[2] A solution of the compound of interest (foldamer **1**, Cu[**2**]Cl_2, Cu[**1**]Cl_2, dibenzo-18-crown-6) in chloroform (1 mM, 10 mL) was set in a glass U-tube (20 mm internal diameter) to form the bottom layer in the tube. To the right side of the U-tube was added a sodium picrate solution (2.5 mL, [picrate] = 436 µM, 20 mM MOPS, 100 mM NaCl, pH 7.4) and to the left side was added MOPS buffer (2.5 mL, 20 mM MOPS, 100 mM NaCl, pH 7.4) as a receiving phase. The U-tube was incubated in a water bath at 25 °C and the chloroform phase was stirred at 300 rpm during the entire experiment, ensuring efficient diffusion of any potential carrier-ion complex to the receiving phase. Aliquots (1 mL) were taken from the receiving phase and analysed for the presence of picrate by UV spectroscopy (at 356 nm). After measurement, the sample was immediately replaced back in the U-tube. Measurements were then taken at 0 h, 1 h, 2 h and 24 h.

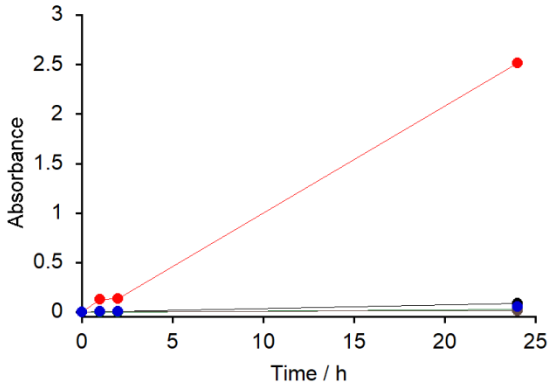

Figure S11: U-tube metal picrate transport data for foldamer **1** (brown), Cu[**2**]Cl_2 (black), Cu[**1**]Cl_2 (green), and the control transporter, dibenzo-18-crown-6 (red). Absorbance at 356 nm.

5.9 U-tube lucigenin transport experiments

The used procedure was developed by modification of published methods.[11] A solution of either **2**, Cu[**1**]Cl_2, Cu[**2**]Cl_2, alamethicin or 2-aminopentane in chloroform (1 mM, 10 mL) was carefully added in a glass U-tube (20 mm internal diameter) to form the bottom layer in the tube. To the right side of the U-tube was added a lucigenin solution (2.5 mL, [lucigenin] = 500 µM, 20 mM MOPS, 100 mM NaCl, pH 7.4) and to the left side was added MOPS buffer (2.5 mL, 20 mM MOPS, 100 mM NaCl, pH 7.4) as a receiving phase. The U-tube was

incubated in a water bath at 25 °C and the chloroform phase was stirred at 300 rpm during the entire experiment, ensuring efficient diffusion of any potential carrier-ion complex to the receiving phase. Aliquots (1 mL) were taken from the receiving phase and analysed for the presence of lucigenin by UV spectroscopy (at 455 nm). After measurement, the sample was immediately replaced back in the U-tube. Measurements were then taken at 0 h, 1 h, 2 h, 3 h, 4 h and 24 h.

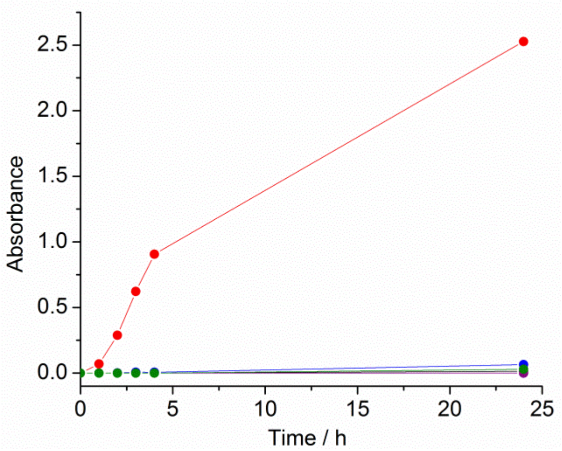

Figure S12: Lucigenin U-tube experiment: 2-aminopentane (●), alamethicin (●), compound **2** (●), Cu[**2**]Cl_2 (●), Cu[**1**]Cl_2 (●). Absorbance at 455 nm.

5.10 Procedure for CF assays

Samples were prepared as described for the procedure for HPTS assays. After 1 min 20 µL of the principal compound in methanol was added then after 7 min 40 µL of a 10 % v/v solution of Triton X-100 detergent in MOPS was added to lyse the vesicles. The release of 5/6-carboxyfluorescein from the prepared vesicles was measured by observing the 5/6-CF emission intensity at 517 nm following excitation at 492 nm.

For each experiment, the initial (F_0) and total (F_{Triton}, 10% Triton X-100) fluorescence was determined and used to determine the final value: $(F-F_0)/(F_{Triton}-F_0)$. Following normalization of the 5/6-CF data, the background methanol was subtracted from the raw data, and the intensity of emission (IF) plotted.

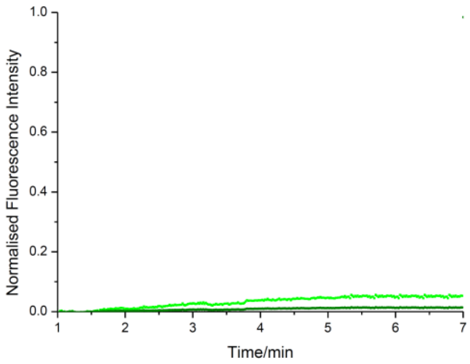

Figure S13: 5/6-CF data for compound Cu[**1**]Cl_2 (pale green squares) and Cu[**2**]Cl_2 (olive green circles).

6. Planar bilayer conductance (PBC) studies

Alamethicin provides a useful comparison to the data reported in the manuscript and in the ESI. Alamethicin is a mildly cation selective channel-former[12] shows non-ohmic behavior and produces discrete events with multiple conductance levels (0.02 nS to 0.15 nS, with open lifetimes in the order of 50 to 80 ms).[13,14] The values found can also be compared to an *N*-acetylated Aib octamer studied previously (0.34 nS at 100 mV)[15] and the conductances typically found for channels (1 to 100 pS) and pores (0.1 to 5 nS).[16]

6.1 Procedure

Single channel experiments were performed in a custom built cell. The cell (Figure S13) was formed of two Teflon blocks, each with a machine-drilled well (approx. 10 mm diameter and 1 mL volume). Each well contained a side opening, such that when the blocks were bolted together the adjacent side openings connected the two wells. Additionally, each well contained two access channels (approx. 2 mm diameter), drilled at a 45° angle, such that the channels joined the bottom of the main well. An aperture (approx. 100 mm diameter) was created in a Teflon sheet (Goodfellow, 25 mm thick) using a 30 kV spark gap generator (Ealing Spark Source). The Teflon sheet containing the aperture was clamped and sealed with silicone glue (3140 RTV coating, Dow Corning) between the two blocks, such that the aperture was positioned in the central lower half of the side opening between the wells.

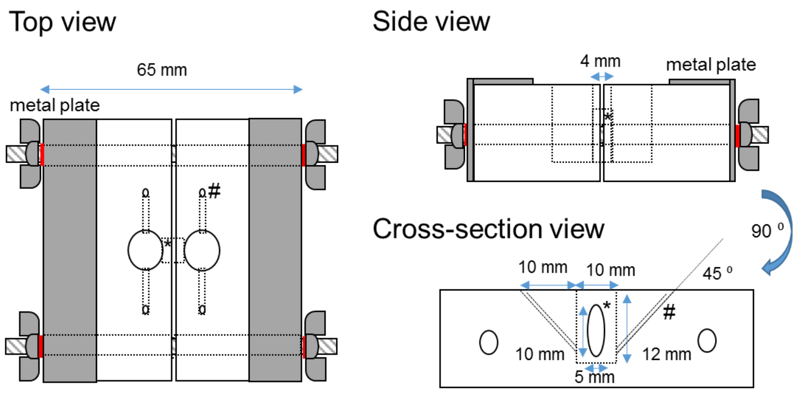

Figure S14: Schematic representation of the cell used in all single channel experiments.

A hanging drop of hexadecane in *n*-pentane (5 mL, 10%, *v*/*v*) was touched on each side of the Teflon sheet and allowed to dry for 1 minute. MOPS buffer (20 mM MOPS, 100 mM KCl or NaCl, pH 7.4) (600 mL) was added to the well each side of the aperture. EYPC lipid/cholesterol (4:1, *w*/*w*) solution (approx. 10 mL, 10 mg mL^{-1}) was added to each side of the well, and left for approx. 5 mins to allow the pentane to evaporate. The cell was subsequently placed into a Faraday cage and Ag/AgCl electrodes (Warner), connected to a patch clamp amplifier (Axopatch 200B, Molecular Devices), were suspended either side of the Teflon sheet. The buffer solution on both sides of the Teflon sheet was aspirated and dispensed using a Hamilton syringe

to ‘paint’ a phospholipid bilayer across the aperture. A ± 1 mV pulse was applied at 1333 Hz to determine when a bilayer was obtained (capacitance of 40 to 80 pF). The membrane was characterized with successive 2 second sweeps under an applied potential ranging from +100 to −100 mM. The membrane was deemed acceptable if the range of current flow across the membrane measured < 1 pA in > 10 consecutive characterization sweeps.

The appropriate foldamer (5–10 mL of a 1 mM solution in MeOH) was added to the ground well. Characterization sweeps were continued for 2 hours, or until substantial channel-forming activity was observed.

All data were collected using the patch clamp amplifier, and digitised (Axon Instruments Digidata 1332A) at a sample rate of 50 kHz with a 2 kHz Lowpass Bessel Filter. All single channel experiments were conducted under an applied potential of +100 mV in 50 second sweeps. At the start and end of each sweep the applied potential was reduced to 0 mV. Single-channel ion current recordings were processed with Clampex 10.2 and Clampfit 10.2 software, where baseline correction was applied and the data were filtered with a Lowpass Bessel (8-pole) filter with a 200 Hz −3 dB cut off.

6.2 PBC data for foldamer 2 and complex Cu(II)[2]Cl_2

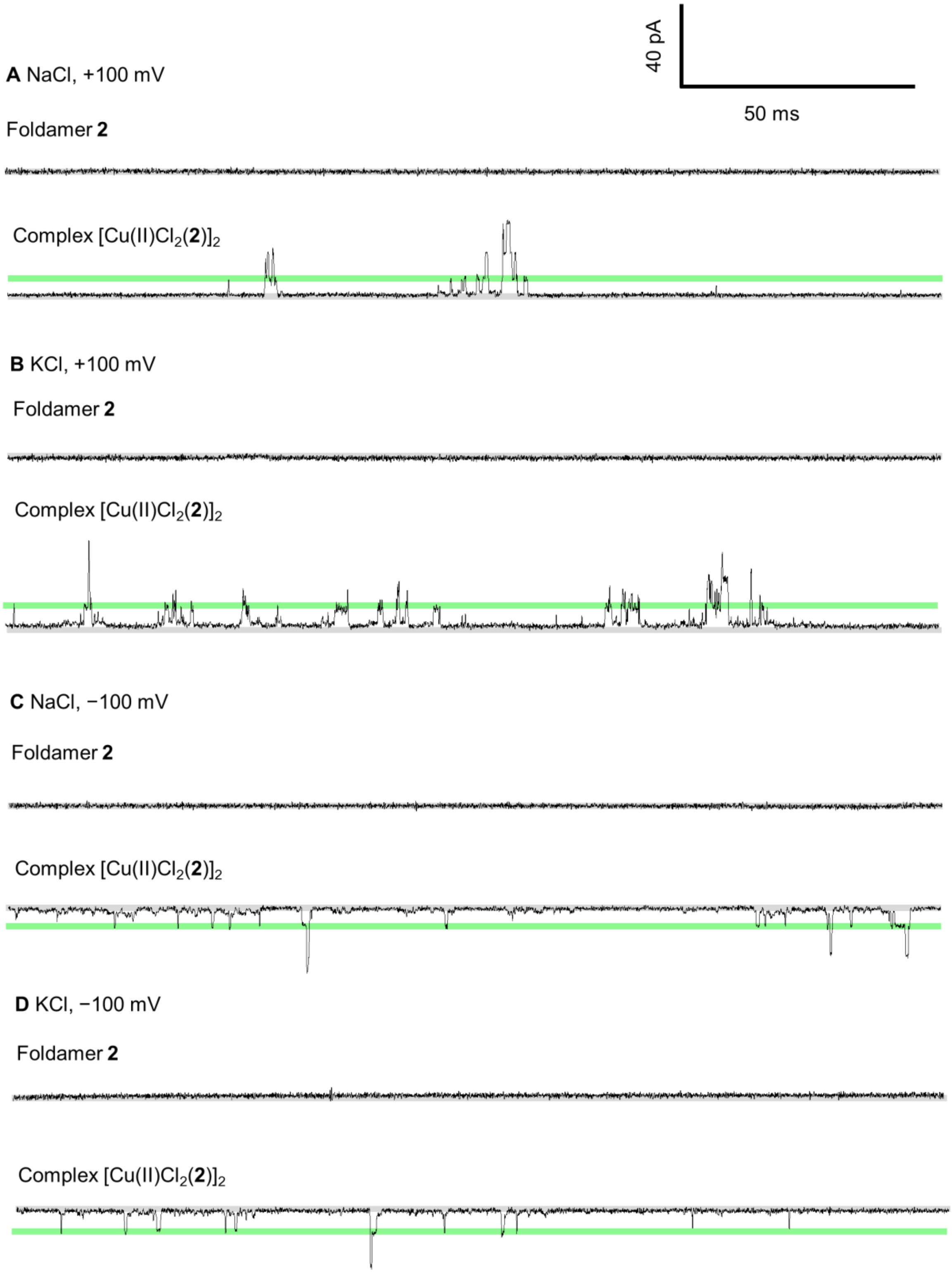

Figure S15: Step changes in conductance induced by foldamer 2 and complex Cu(II)[**2**]Cl_2 (final concentration 8.3 µM) added to cis side. Membranes were formed from EYPC lipid/cholesterol (4:1, w/w) in MOPS buffer at room temperature (20 mM MOPS, 100 mM NaCl or KCl, pH 7.4) at −100 mV or +100 mV. Green bars mark the approximate level of a single quantized current-step.

6.3 I-V curves for EYPC lipid/cholesterol bilayers with and without foldamer 2

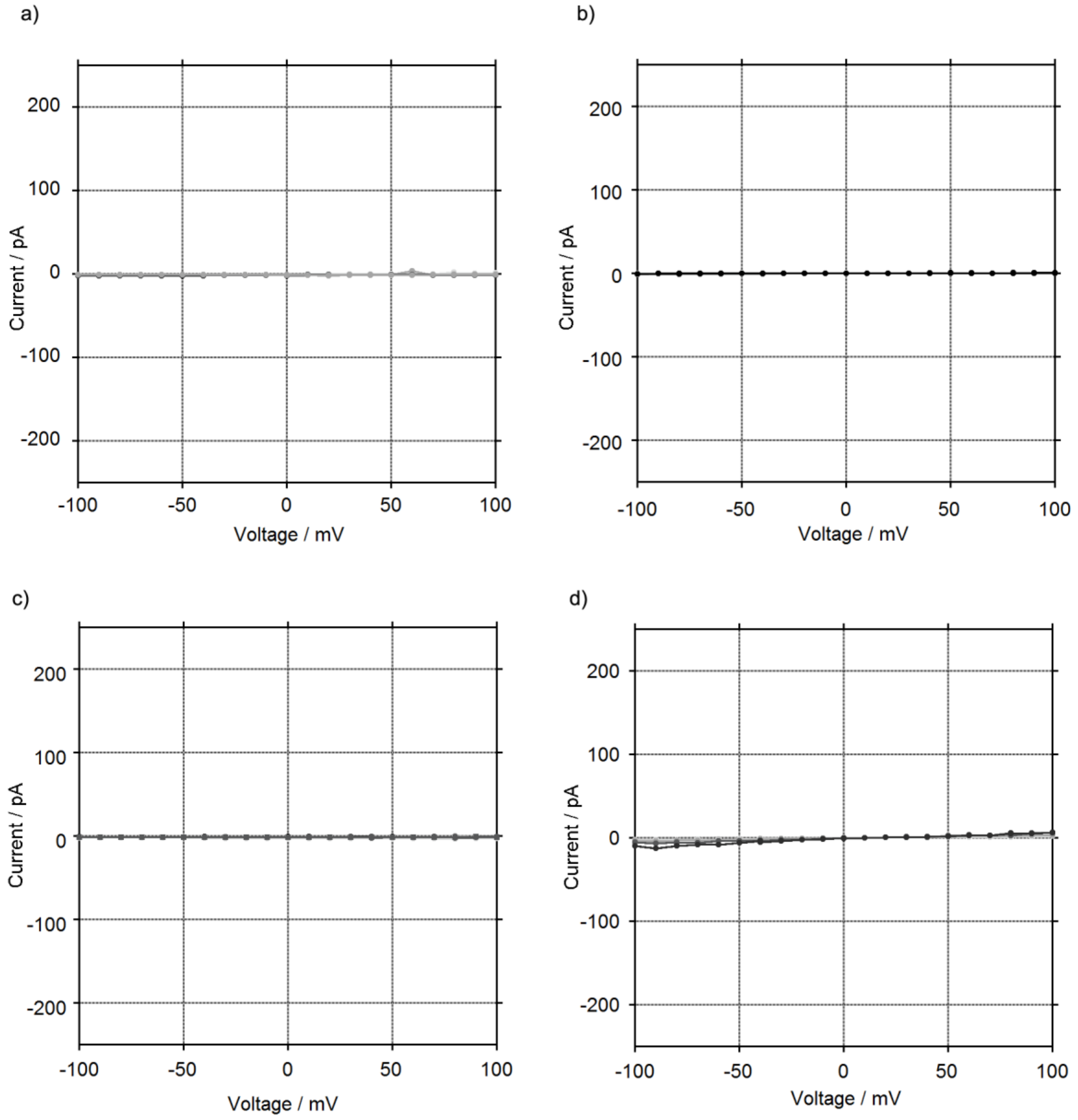

Figure S16: Current-voltage curves for EYPC lipid/cholesterol (4:1, w/w) in MOPS buffer at room temperature (20 mM MOPS, pH 7.4) with (a) 100 mM NaCl; (b) 100 mM NaCl with foldamer **2** (8.3 µM); (c) 100 mM KCl; (d) 100 mM KCl with foldamer **2** (8.3 µM).

6.4 Repeated I-V curves for EYPC lipid/cholesterol bilayers with complex Cu(II)[2]Cl_2

Conductance was measured in increasing increments of 10 mV up to +100 mV, before the current was reduced to -40 mV in the following steps (+80 mV, +40 mV, 0 mV, -40 mV). This procedure provided two measurements at +80 mV, +40 mV, 0 mV and -40 mV. The current was found to increase over time (Figure S16), which we ascribe to slow insertion of the foldamer into the membrane, an equilibration process that is accelerated under strongly positive and especially strongly negative potential differences.[17]

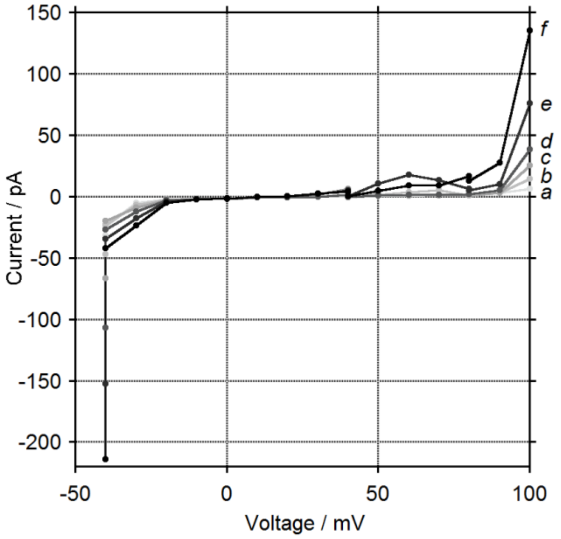

Figure S17: Six repeats (*a* to *f*) of the current-voltage trace for EYPC lipid/cholesterol (4:1, w/w) in MOPS buffer at room temperature (100 mM KCl, 20 mM MOPS, pH 7.4) with complex Cu(II)[**2**]Cl_2 (8.3 µM) added to *cis* side. Trace *a* is the first, *f* is the last. The increase at 60 mV in trace *e* is believed to be an artefact.

6.5 PBC data for the product from complexation of CuCl with 2 (Cu(II)[2]Cl.HCO_3)

Under the same conditions employed for foldamer **2** and complex Cu(II)[**2**]Cl_2, the complex Cu(II)[**2**]Cl.HCO_3 showed a mixture of behaviours. Short-lived "flicker"-type openings could be observed (Figure S18), which displayed conductance levels (0.07 ± 0.01 nS, 0.16 ± 0.01 nS) very similar to those observed for Cu(II)[**2**]Cl_2. In addition, much longer lived "square topped" openings could be observed (Figure S19), which had very regular well-defined quantized current steps with large current levels (0.18 ± 0.01 nS) that were open for up to 20 ms at +100 mV). Although small differences in pore structure are possible between complex Cu(II)[**2**]Cl_2 and complex Cu(II)[**2**]Cl.HCO_3, we expect respective pore structures to be similar as the precursor complexes only differ in counterion; indeed the higher conductance level of the "blue" complex (0.18 ± 0.01 nS) is not dissimilar to the higher conductance level of the "green" complex (0.15 ± 0.01 nS). These small differences in conductance level could arise as a result of experimental variation in these EPYC/cholesterol membranes, which are less stable than the standard diphytanoyl phosphatidylcholine membranes typically used for PBC measurements (EPYC/cholesterol bilayers typically displayed capacitance values between 30 and 60 pF). We speculate the greater prevalence of the high conductance channels and their longer open duration in some samples might be attributed to a greater concentration of a channel forming species in the membrane for the more soluble "blue" complex, which diffuses more rapidly into the membrane to produce more frequent, longer-lived openings of larger, higher nuclearity pores.

6.5.1 Short lived openings

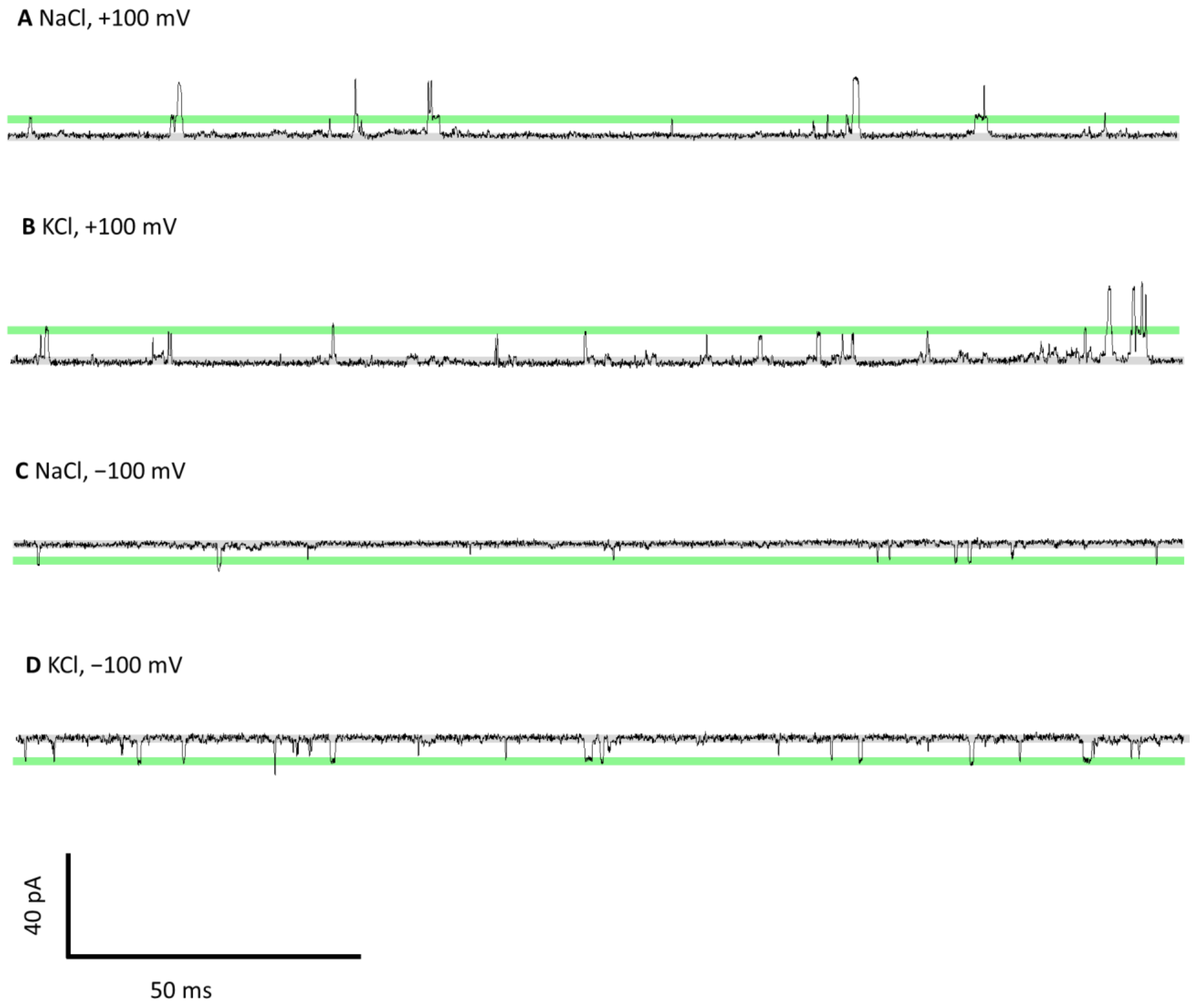

Figure S18: Step changes in conductance induced by Cu(II)[**2**]Cl.HCO_3 (final concentration 8.3 µM) added to *cis* side. Membranes were formed from EYPC lipid/cholesterol (4:1, w/w) in MOPS buffer at room temperature (20 mM MOPS, 100 mM NaCl or KCl, pH 7.4) at −100 mV or +100 mV. Green bars mark the approximate level of a single quantized current-step.

6.5.2 Long lived openings

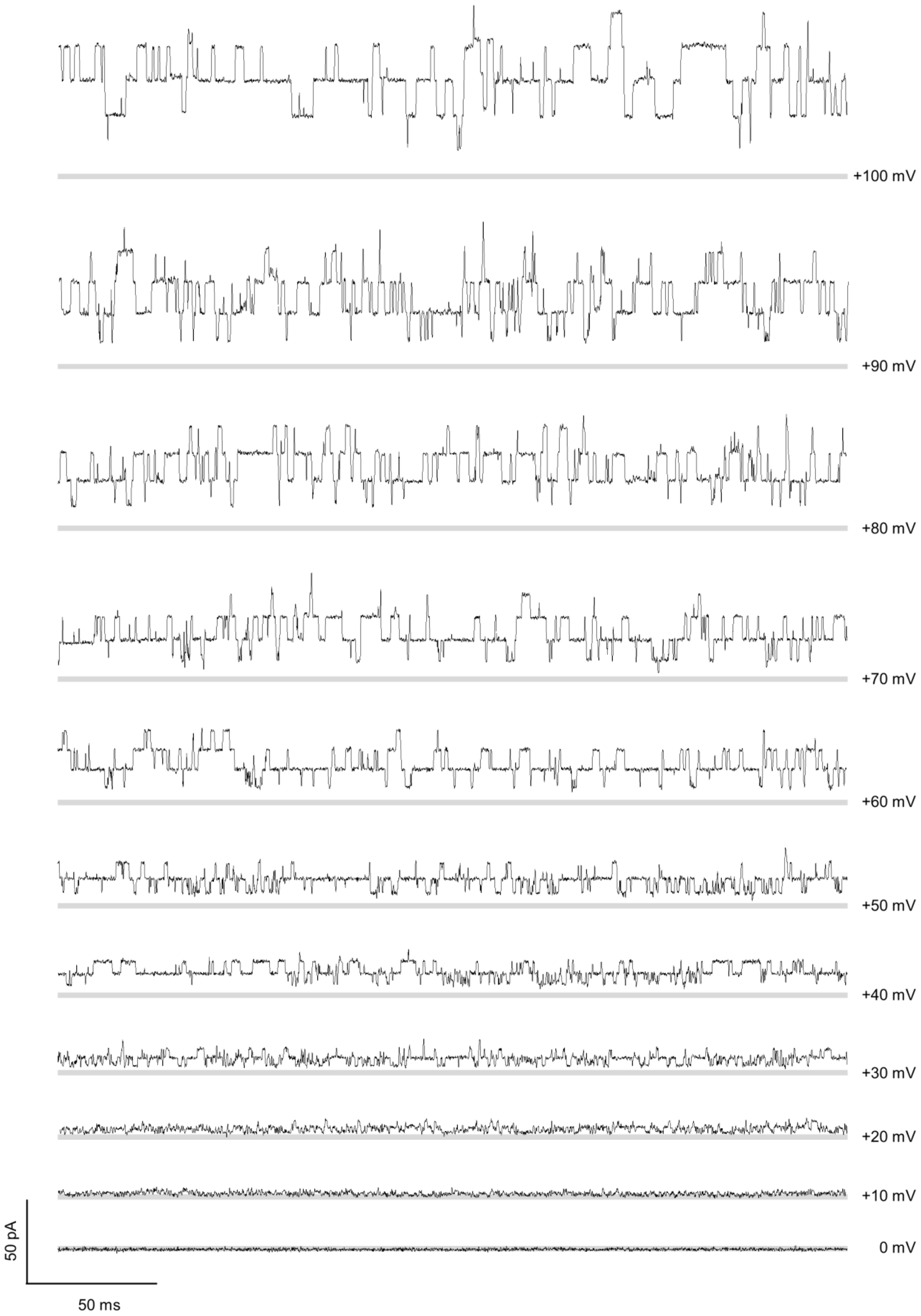

Figure S19: PBC data for Cu(II)[**2**]Cl.HCO_3 (8.3 mM) in MOPS buffer (20 mM MOPS, 100 mM NaCl, pH 7.4).

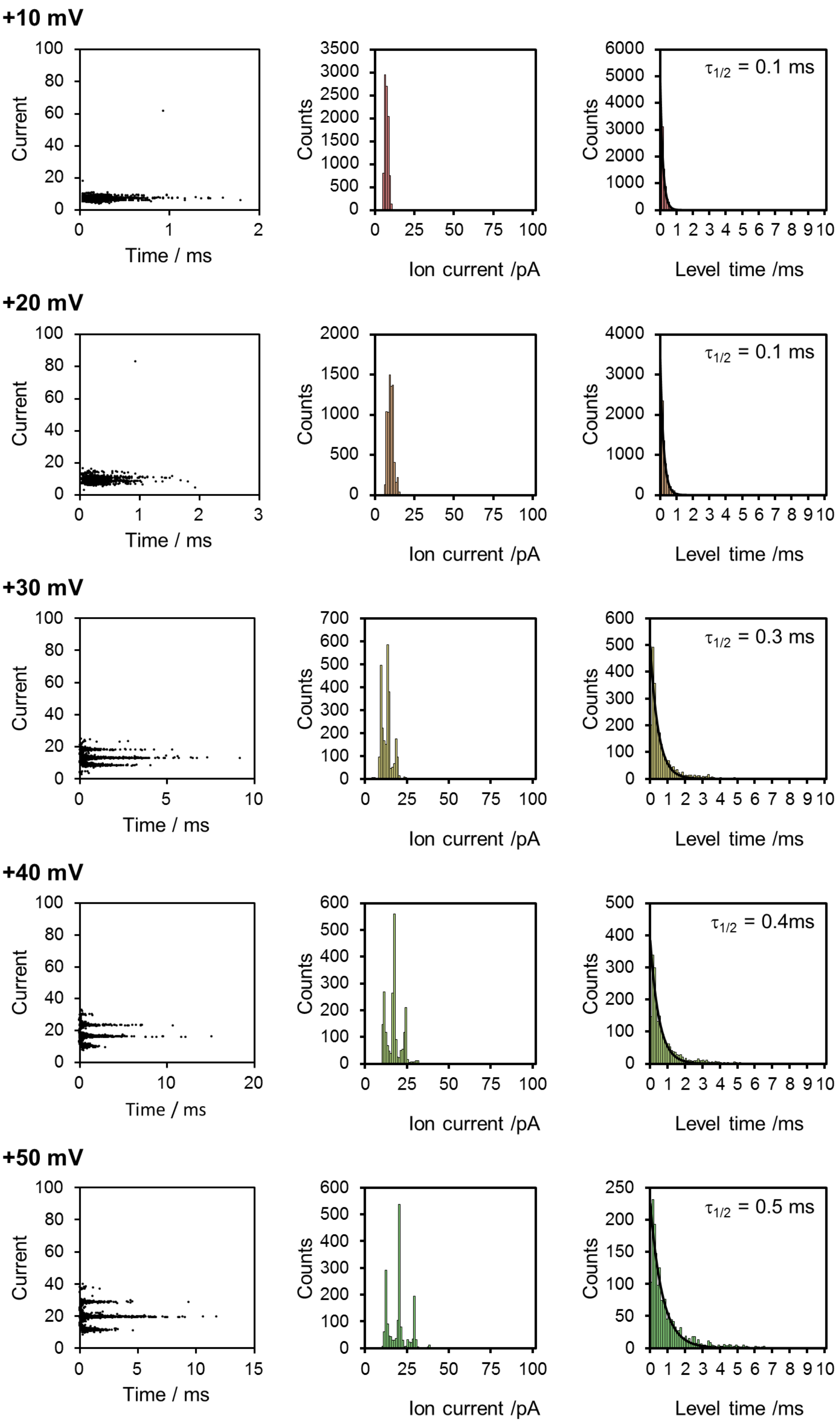

Figure S20: Quantized behaviour of channel formation of Cu(II)[**2**]Cl.HCO_3 (8.3 mM) in MOPS buffer (20 mM MOPS, 100 mM NaCl, pH 7.4).

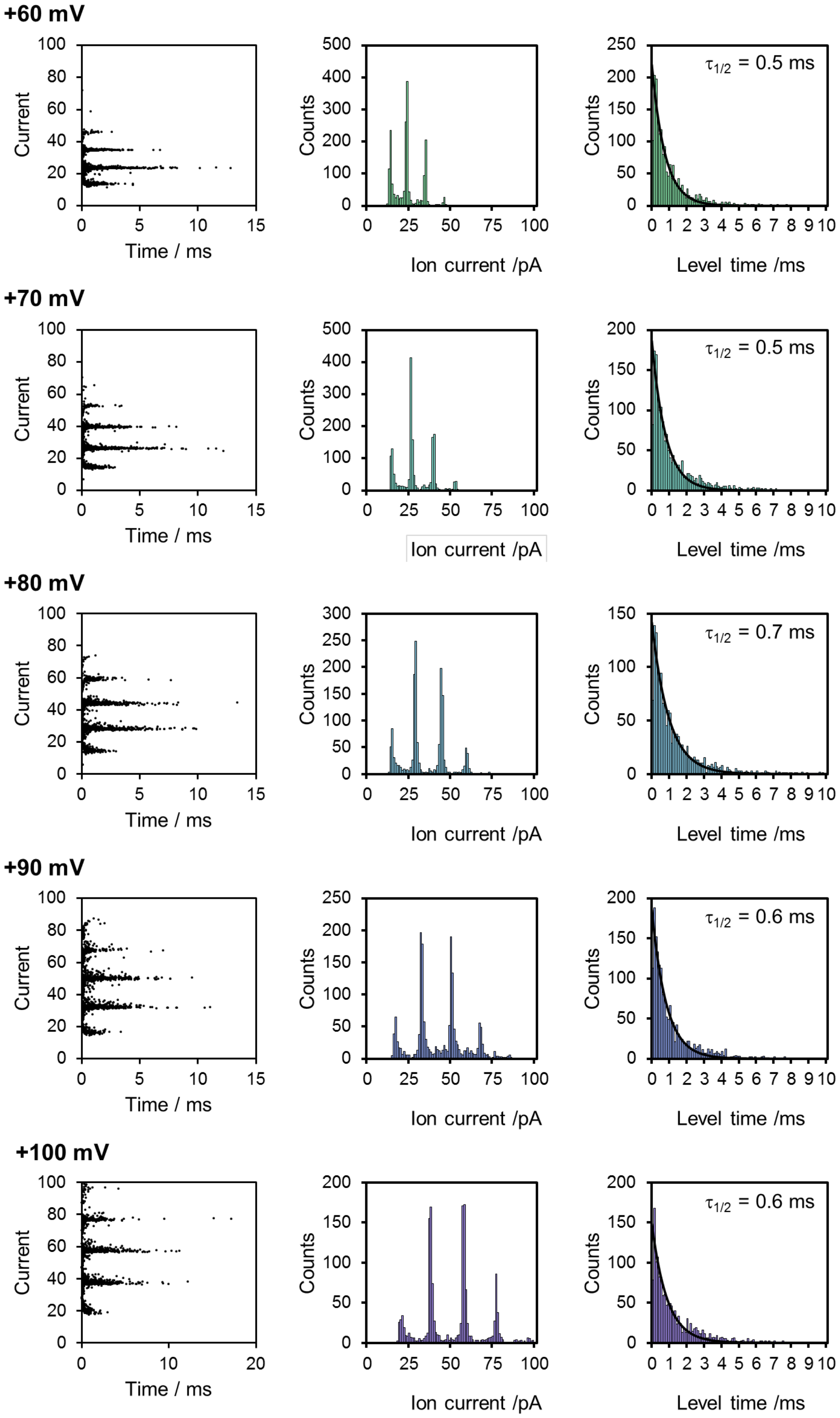

Figure S21: Quantized behaviour of channel formation of Cu(II)[**2**]Cl.HCO_3 (8.3 mM) in MOPS buffer (20 mM MOPS, 100 mM NaCl, pH 7.4).

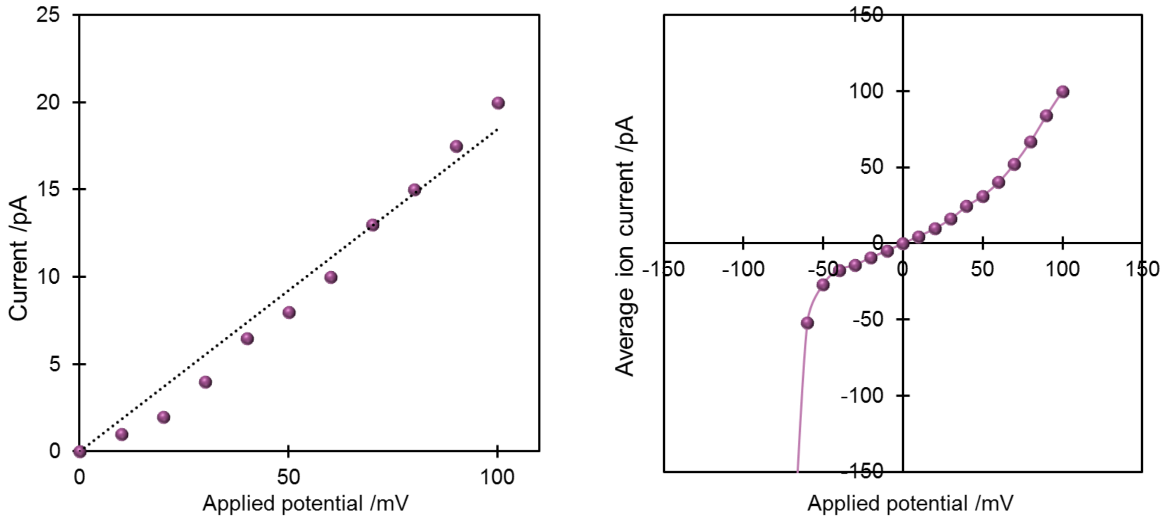

Figure S22: The current of a single quantized current-step at a range of applied voltages reveals an approximately linear relationship. Cu(II)[**2**]Cl.HCO_3 (8.3 mM) in MOPS buffer (20 mM MOPS, 100 mM NaCl, pH 7.4). Left: 0 to 100 mV. Right: −150 to 150 mV.

6.6 Estimation of channel diameter

We have used the Hille equation[18] to estimate the pore sizes that correspond to the different conductance levels observed:

Table S2. Corrected Hille calculation for compound ([Cu(II)[**2**](μ-Cl)]$_2$Cl$_2$

Conditions	Conductance	Correction factor	Estimated diameter
+100 mV in NaCl	~0.08 ± 0.01 nS	5.51 (alamethicin hexamer)	1.45 - 1.67 nm
	~0.14 ± 0.01 nS	5.05 (alamethicin heptamer)	1.97 – 2.15 nm
+100 mV in KCl	~0.10 ± 0.01 nS	5.51 (alamethicin hexamer)	1.49 - 1.67 nm
-100 mV in NaCl	~0.08 ± 0.01 nS	5.51 (alamethicin hexamer)	1.45 - 1.67 nm
-100 mV in KCl	~0.10 ± 0.01 nS	5.51 (alamethicin hexamer)	1.49 - 1.67 nm

Table S3. Corrected Hille calculation for compound ([Cu(II)[**2**]Cl].HCO_3

Conditions	Conductance	Correction factor	Estimated diameter
+100 mV in NaCl	~0.07 ± 0.01 nS	5.51 (alamethicin hexamer)	1.33 - 1.56 nm
	~0.18 ± 0.01 nS	4.64 (alamethicin octamer)	2.20 - 2.35 nm
+100 mV in KCl	~0.11 ± 0.01 nS	5.51 (alamethicin hexamer)	1.58 – 1.76 nm
-100 mV in NaCl	~0.07 ± 0.01 nS	5.51 (alamethicin hexamer)	1.33 - 1.56 nm
-100 mV in KCl	~0.09 ± 0.01 nS	5.51 (alamethicin hexamer)	1.39 - 1.58 nm

These different conductance levels were assumed to be due to the formation of different multimeric pores, with the lower levels proposed to be due to structures analogous to hexameric alamethicin pores, the smallest stable alamethicin pore, intermediate levels due to structures analogous to heptameric alamethicin pores, and highest level due to structures analogous to octameric alamethicin pores. The correction factors calculated by Sansom and co-workers[19] for these different multimeric alamethicin pores were applied to these experimental conductances (a factor of 5.51 for hexameric pores, a factor of 5.05 heptameric pores, and 4.64 for octameric pores). The corrected values (g) were applied in the Hille equation:

$$(g)^{-1} = \frac{4l\rho}{\pi d^2} + \frac{\rho}{d}$$

Where l = 3.4 nm (channel length, the approximate width of an EYPC bilayer),[20] and ρ = 0.77 (for 0.1 M KCl)[20,21] and ρ = 0.94 (for 0.1 M NaCl).[21]

These calculations provide approximate pore diameters of 1.5 nm, 2.1 nm and 2.3 nm for the different conductance levels. A good correlation is observed between pore diameters calculated in KCl and NaCl for the lowest conductance levels. Comparison to published data for the size of pores formed by alamethicin shows these estimates have a reasonable correlation with proposed inner diameters of hexameric pores (1.1 nm diameter, determined by high resolution electrochemical scanning tunnelling microscopy)[22] and octameric pores (1.8 nm diameter, determined by x-ray diffraction)[23] formed by alamethicin.

7. Antimicrobial assays

Metal ion chelating Aib foldamers **1** and **2** bear a novel amino-terminal copper and nickel (ATCUN) binding motif; other ATCUN motifs are reported to increase antibiotic properties by generating reactive oxygen species upon metal binding.[24] Furthermore many copper-containing metallodrugs have been studied for their anticancer[25] and antibiotic properties.[26] Introducing the Cu(II)(BPTA) group onto the Aib octamer unit also increased water solubility, mitigating to some extent the solubility problems that hampered earlier antibiotic studies of long Aib_n oligomers ($n \geq 10$).[15]

7.1 Antimicrobial assays: minimum inhibitory concentrations (MIC)

The used procedure was developed by modification of published methods.[27] The antimicrobial activity of the appropriate compound was tested against *B. megaterium* strain DSM319. Bacteria were grown over night at 30 °C in standard lysogeny broth (LB) broth from a single colony overnight. An aliquot from this suspension was taken and diluted (1:100) in fresh LB broth until the suspension reached the mid-logarithmic phase (OD_{600} = 0.4).

Aliquots of this suspension were diluted with LB broth dispensed into sterile flat bottom 96 well plates to obtain approximately 3×10^6 CFU/mL in a total volume of 200 µL. Serial solutions of the appropriate compound (foldamers **1**, **2**, Cu[**1**]Cl_2 and Cu[**2**]Cl_2) dissolved in DMSO were added into the wells in triplicate. The plates were shaken for about 30 seconds and the optical density (OD) was measured at 600 nm. Afterwards the plates were incubated at 30 °C under aerobic conditions with shaking overnight. The absorbance was then measured at 600 nm using a ClarioStar plate reader. The minimum inhibitory concentration (MIC) was defined as the lowest antibiotic concentration that completely inhibited the growth of the tested bacteria. The assays were performed in three biological repeats with three technical repeats of each experiment.

The concentration of the tested compound which caused an OD equal to the OD of LB (± 5%) was considered as the MIC.

7.2 Haemolysis assays

Red blood cells were collected with full Research Ethics Committee (REC) approval for use in evaluation of new treatment strategies (REC reference 10/H1017/73). All samples were collected with full consent for research use with identity traced only through the collecting medical institution (Manchester Royal Infirmary, custodian Dr John Burthem). There are no direct or indirect medical implications for the donor. The used procedure was developed by modification of published methods.[28] Freshly taken blood was diluted with PBS buffer (1:2). Aliquots (500 µL) of this diluted blood were placed into an Eppendorf tube. Aliquots (5 µL) of serial solutions of the foldamers **1**, **2**, Cu[**1**]Cl_2 and Cu[**2**]Cl_2 in DMSO, as well as DMSO itself as a blank and 1% Triton X-100 as 100% lysis of the cells were added to the Eppendorf tubes in triplicates. The resulting mixture was gently shaken to mix well and incubated at 37 °C for 30 min, followed

by centrifugation at 1200 rpm for 30 seconds. Aliquots (50 µL) of the supernatant (50 µL) were transferred into a new sterile 96-well plate containing PBS buffer (150 µL) in each well. The release of haemoglobin was measured at 560 nm using a microtiter plate reader. Percentage haemolysis was calculated using:

$$\text{percentage haemolysis} = (A_{\text{compound}} - A_{\text{DMSO}}) / (A_{\text{complete haemolysis}} - A_{\text{DMSO}}) \times 100\%$$

where complete haemolysis was achieved by mixing the erythrocytes with 1% Triton X-100.

The channel concentration required to cause 50% haemolysis (HC_{50}) was determined directly from the graph.

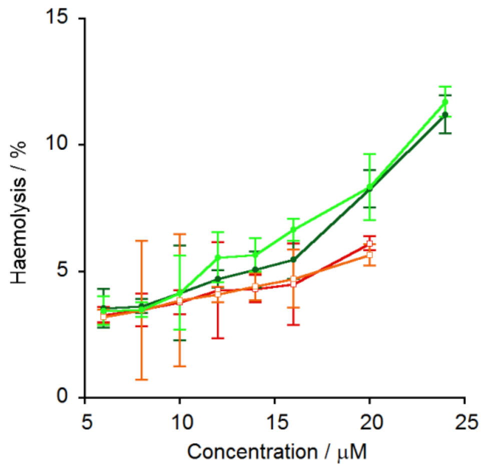

Figure S23: Haemolysis of human erythrocytes caused by alamethicin (black) and compound **1** (orange), **2** (red), Cu[**1**]Cl_2 (pale green) and Cu[**2**]Cl_2 (dark green).

8. Crystal data and structure refinement

8.1 Foldamer 2

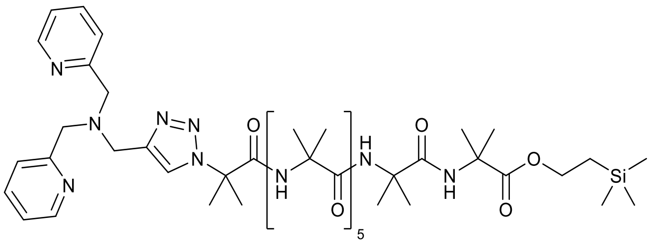

Single crystals suitable for X-ray diffraction analysis were grown by slow diffusion of diethyl ether in with compound **2** saturated solution of chloroform. Data were collected on a dual source Rigaku FR-X rotating anode diffractometer using CuK_α wavelength radiation (λ = 1.54184) at a temperature of 220 K. The data were reduced using CrysAlisPro 171.39.27b and absorption correction was performed using empirical methods (SCALE3 ABSPACK) based upon symmetry-equivalent reflections combined with measurements at different azimuthal angles.[29] The structure was solved and refined against F^2 using Shelx-2014/6 implemented through Olex2 v1.2.9.[30] Crystals of the peptide **2** are triclinic, space group = P-1, with unit-cell dimensions of a = 9.2070(3), b = 15.6088(3), c = 23.6308(5) Å. R-factor (%) = 8.09.

Table S4. Crystal data and structure refinement for foldamer **2**

Identification code	s4824l
Empirical formula	$C_{53.81}H_{87.52}N_{13}O_{9.45}Si$
Formula weight	1095.83
Temperature/K	220.01(10)
Crystal system	triclinic
Space group	P-1
a/Å	9.2070(3)
b/Å	15.6088(3)
c/Å	23.6308(5)
α/°	85.168(2)
β/°	79.223(2)
γ/°	76.681(2)
Volume/Å^3	3243.35(14)
Z	2
ρ_{calc}g/cm^3	1.122
μ/mm^{-1}	0.801
F(000)	1182.0
Crystal size/mm^3	0.25 × 0.1 × 0.1
Radiation	CuKα (λ = 1.54184)
2Θ range for data collection/°	3.81 to 145.764
Index ranges	-9 ≤ h ≤ 11, -19 ≤ k ≤ 19, -29 ≤ l ≤ 29
Reflections collected	73644
Independent reflections	12599 [R_{int} = 0.0399, R_{sigma} = 0.0230]
Data/restraints/parameters	12599/832/866
Goodness-of-fit on F^2	1.050
Final R indexes [I>=2σ (I)]	R_1 = 0.0809, wR_2 = 0.2505
Final R indexes [all data]	R_1 = 0.0939, wR_2 = 0.2641
Largest diff. peak/hole / e Å^{-3}	1.12/-0.50

8.2 Copper complexes

The structure of copper complexes of **2** can be compared to published structures of copper salts with *N*,*N*,*N*-tris(pyridin-2-ylmethyl)amine (TPA). Cu(II)(TPA)Cl^+ complexes show a small outwards displacement (*ca*. 0.34 Å), Cu(II)-Cl bond lengths in the range 2.233 to 2.244 Å, with distances between the metal and the central nitrogen typically 2.03 to 2.06 Å.[31,32,33,34] In contrast, Cu(I)(TPA)Cl complexes show more pronounced outwards displacement of the copper(I) from the plane derived from the equatorial N atoms (0.534 Å, 0.578 Å), longer Cu(I)-Cl bond lengths of 2.398 and 2.360 Å and very weak interaction with the central alkyl nitrogen (distances from 2.437 to 2.468 Å).[31,35]

8.2.1 Complex Cu(II)[2]Cl.HCO_3

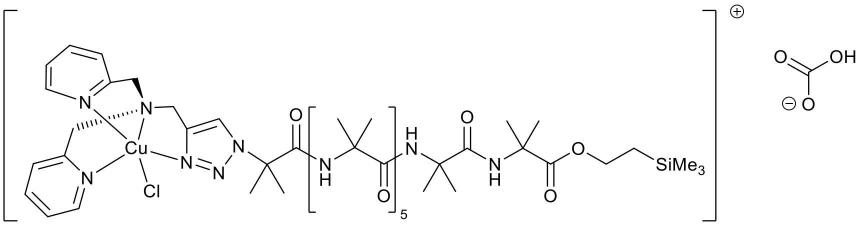

Single crystals suitable for X-ray diffraction analysis were grown by slow diffusion of diethyl ether in with compound Cu[**2**]Cl.HCO_3 saturated solution of chloroform. Data were collected on a dual source Rigaku FR-X rotating anode diffractometer using CuK_α wavelength radiation (λ = 1.54184) at a temperature of 150 K. The data were reduced using CrysAlisPro 171.39.30c and absorption correction was performed using empirical methods (SCALE3 ABSPACK) based upon symmetry-equivalent reflections combined with measurements at different azimuthal angles.[29] The structure was solved and refined against F^2 using Shelx-2018/3 implemented through Olex2 v1.2.9.[30] Crystals of the peptide Cu[**2**]Cl.HCO_3 are orthorhombic, space group = $P2_12_12_1$, with unit-cell dimensions of a = 9.1099(2), b = 24.1351(9), c = 34.1137(11) Å. R-factor (%) = 7.18.

Table S5. Crystal data and structure refinement for Cu[**2**]Cl.HCO_3

Identification code	s5009r
Empirical formula	$C_{53}H_{84}ClCuN_{13}O_{12}Si$
Formula weight	1222.41
Temperature/K	150.00(2)
Crystal system	orthorhombic
Space group	$P2_12_12_1$
a/Å	9.1099(2)
b/Å	24.1351(9)
c/Å	34.1137(11)
α/°	90
β/°	90
γ/°	90
Volume/Å^3	7500.6(4)
Z	4
ρ_{calc}g/cm^3	1.083
μ/mm^{-1}	1.354
F(000)	2596.0
Crystal size/mm^3	0.329 × 0.014 × 0.01
Radiation	CuKα (λ = 1.54184)
2Θ range for data collection/°	4.484 to 117.844
Index ranges	-10 ≤ h ≤ 9, -24 ≤ k ≤ 26, -37 ≤ l ≤ 29
Reflections collected	40375
Independent reflections	10629 [R_{int} = 0.1119, R_{sigma} = 0.0849]
Data/restraints/parameters	10629/253/785
Goodness-of-fit on F^2	1.052
Final R indexes [I>=2σ (I)]	R_1 = 0.0715, wR_2 = 0.1715
Final R indexes [all data]	R_1 = 0.1081, wR_2 = 0.1949
Largest diff. peak/hole / e Å^{-3}	0.34/-0.50
Flack parameter	0.04(2)

8.2.2 Complex Cu(II)[2]Cl_2

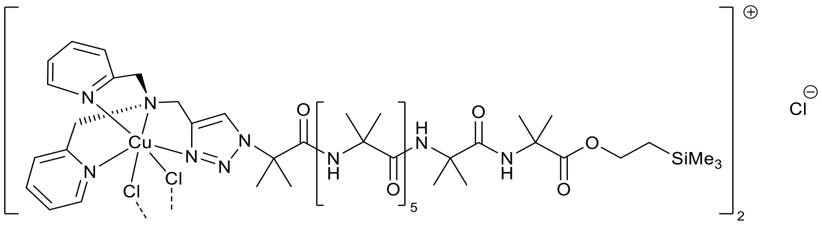

Single crystals suitable for X-ray diffraction analysis were grown by slow diffusion of diethyl ether in with compound Cu[**2**]Cl_2 saturated solution of chloroform. Data were collected on a dual source Rigaku FR-X rotating anode diffractometer using CuK_α wavelength radiation (λ = 1.54184) at a temperature of 150 K. The data were reduced using CrysAlisPro 171.39.30c and absorption correction was performed using empirical methods (SCALE3 ABSPACK) based upon symmetry-equivalent reflections combined with measurements at different azimuthal angles.[29] The structure was solved and refined against F^2 using Shelx-2018/3 implemented through Olex2 v1.2.9.[30] Crystals of the peptide Cu[**2**]Cl_2 are triclinic, space group = P-1, with unit-cell dimensions of a = 8.9253(5), b = 15.5724(9), c = 25.2322(14), Å. R-factor (%) = 7.16.

The copper ions show a Jahn-Teller distortion that is consistent with Cu(II), with elongation of one Cu-Cl bond (2.836 Å) compared to the other (2.241 Å). The triazole opposite the elongated Cu(II)-Cl bond is inclined relative to the N-Cu-Cl axis and has a lengthened bond to Cu(II) of 2.663 Å, much longer than the bonds to the pyridyl nitrogens (1.985 and 1.981 Å). The Jahn-Teller distortion around the Cu(II) centers is similar to that reported for a dimeric $CuCl_2$/TPA complex, which shows an inclined pyridyl group with a long bond to Cu(II) of 2.52 Å.[36]

Table S6. Crystal data and structure refinement for Cu[**2**]Cl_2**:**

Identification code	s5222r
Empirical formula	$C_{52}H_{83}Cl_2CuN_{13}O_9Si$
Formula weight	1196.84
Temperature/K	149.99(10)
Crystal system	triclinic
Space group	P-1
a/Å	8.9253(5)
b/Å	15.5724(9)
c/Å	25.2322(14)
α/°	98.168(5)
β/°	91.134(5)
γ/°	103.075(5)
Volume/Å^3	3376.5(3)
Z	2
ρ_{calc}g/cm^3	1.177
μ/mm^{-1}	1.815
F(000)	1270.0
Crystal size/mm^3	0.093 × 0.046 × 0.013
Radiation	CuKα (λ = 1.54184)
2Θ range for data collection/°	3.542 to 89.406
Index ranges	-8 ≤ h ≤ 8, -14 ≤ k ≤ 14, -23 ≤ l ≤ 22
Reflections collected	23647
Independent reflections	5344 [R_{int} = 0.0689, R_{sigma} = 0.0684]
Data/restraints/parameters	5344/0/663
Goodness-of-fit on F^2	1.048
Final R indexes [I>=2σ (I)]	R_1 = 0.0716, wR_2 = 0.2020
Final R indexes [all data]	R_1 = 0.0924, wR_2 = 0.2197
Largest diff. peak/hole / e Å^{-3}	0.72/-0.37

9. Electron paramagnetic resonance (EPR) studies

Electron paramagnetic resonance (EPR) spectroscopy was performed on an EMX with a 1.8 T electromagnet in the range from 20 K to 130 K.

The Q band (34 GHz) electron paramagnetic resonance (EPR) data (Figures 24-26) unpaired electrons were present (consistent with Cu(II)). It was consistent with a Cu(II) center with Jahn-Teller distortion,[37] and did not show significant differences upon changing the temperature from 20 K to 130 K (Figure S24), which suggests the $Cu(II)_2$[**2**]$_2Cl_2$ unit has a relatively a rigid structure.

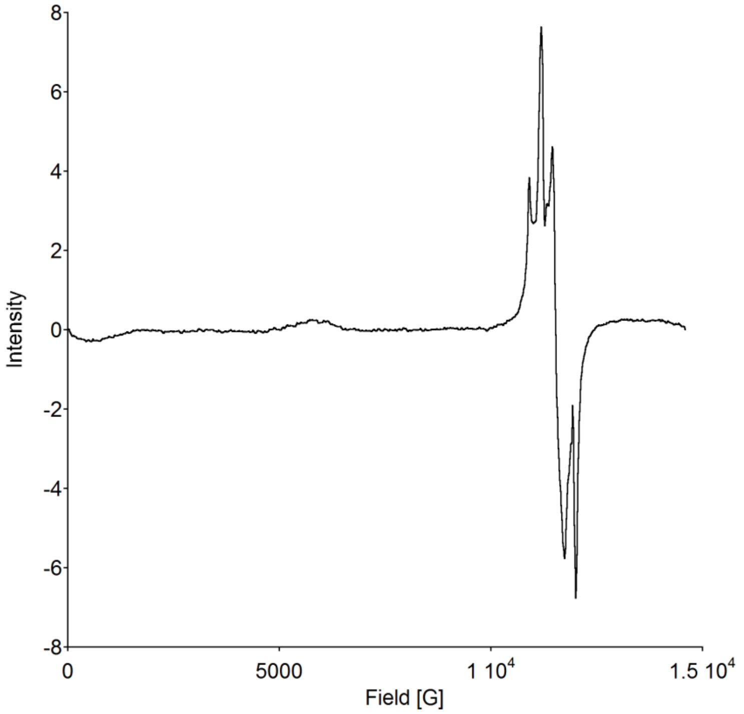

Figure S24: Q-band EPR spectrum of compound Cu[**2**]Cl_2 at 130 K

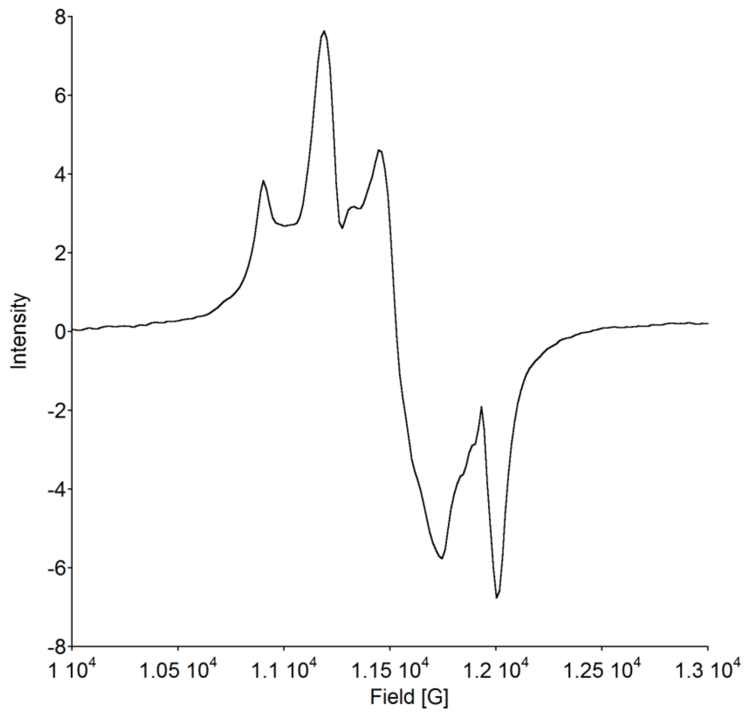

Figure S25: Expanded view of Q-band EPR spectrum of compound Cu[**2**]Cl_2 at 130 K

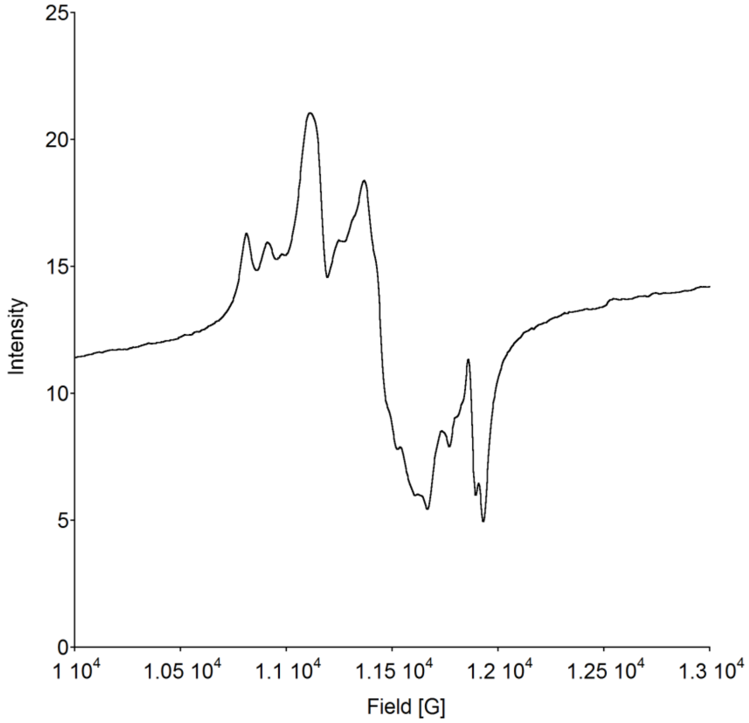

Figure S26: Expanded view of EPR spectrum of compound Cu[**2**]Cl_2 at 20 K (no baseline correction).

10. References

[1] J. Clayden, A. Castellanos, J. Solà and G. A. Morris, *Angew. Chem. Int. Ed.*, 2009, **48**, 5962–5965.
[2] J. E. Jones, V. Diemer, C. Adam, J. Raftery, R. E. Ruscoe, J. T. Sengel, M. I. Wallace, A. Bader, S. L. Cockroft, J. Clayden and S. J. Webb, *J. Am. Chem Soc*. 2016, **138**, 688–695
[3] S. J. Pike, V. Diemer, J. Raftery, S. J. Webb and J. Clayden, *Chem. Eur. J.*, 2014, **20**, 15981–15990.
[4] S. Huang, R. J. Clark and L. Zhu, *Org. Lett.*, 2007, **9**, 4999–5002.
[5] K. Kano and J. H. Fendler, *BBA Biomembranes* 1978, **509**, 289–299.
[6] C. P. Wilson, C. Boglio, L. Ma, S. L. Cockroft and S. J. Webb, *Chem. Eur. J.* 2011, **17**, 3465–3473.
[7] A. F. DiGiorgio, S. Otto, P. Bandyopadhyay and S. L. Regen, *J. Am. Chem. Soc*. 2000, **122**, 11029–11030.
[8] S. M. Elvington and J. W. Nichols, *BBA Biomembranes* 2007, **1768**, 502–508.
[9] S. V. Shinde and P. Talukdar, *Chem. Commun.*, 2018, **54**, 10351-10354.
[10] J. L. Seganish, J. C. Fettinger and J. T. Davis, *Supramol. Chem.*, 2006, **18**, 257–264.
[11] J. Gravel and A. R. Schmitzer, *J. Supramol. Chem*. 2015, **27**, 364–371.
[12] J. E. Hall, I. Vodyanoy, T. M. Balasubramanian and G. R. Marshall, *Biophys. J*. 1984, **45**, 233–247.
[13] C. U. Hjørringgaard, B. S. Vad, V. V. Matchkov, S. B. Nielsen, T. Vosegaard, N. C. Nielsen, D. E. Otzen and T. Skrydstrup, *J. Phys. Chem. B*, 2012, **116**, 7652−7659.
[14] M. S. P. Sansom, *Eur. Biophys. J*. 1993, **22**, 105–124.
[15] C. Adam, A. D. Peters, M. G. Lizio, G. F. S. Whitehead, V. Diemer, J. A. Cooper, S. L. Cockroft, J. Clayden and S. J. Webb, *Chem. Eur. J.* 2018, **24**, 2249–2256.
[16] N. Sakai, J. Mareda and S. Matile, *Acc. Chem. Res*. 2005, **38**, 79–87.
[17] R. U. Muller and O. S. Andersen, *J. Gen. Physiol*. 1982, **80**, 427-449.
[18] Hille B. Ionic Channels of Excitable Membranes, 3rd Edition, Sinauer, Sunderland, MA, 2001.
[19] O. S. Smart, J. Breed, G. R. Smith and M. S. P. Sansom, *Biophys. J.*, 1997, **72**, 1109-1126
[20] N. Sakai, Y. Kamikawa, M. Nishii, T. Matsuoka, T. Kato and S. Matile, *J. Am. Chem. Soc*. 2006, **128**, 2218-2219
[21] P. Vanýsek in "*CRC Handbook of Chemistry and Physics*", 91st edition, Ed. W. M. Haynes, 2010, pg. 5-74
[22] P. Pieta, J. Mirza and J. Lipkowski, *Proc. Natl. Acad. U.S.A.*, 2012, **109**, 21223–21227.
[23] S. Qian, W. Wang, L. Yang and H. W. Huang, *Biophys. J.*, 2008, **94**, 3512–3522.
[24] M. D. Libardo, J. L. Cervantes, J. C. Salazar and A. M. Angeles-Boza, *ChemMedChem*, 2014, **9**, 1892–1901.
[25] C. Santini, M. Pellei, V. Gandin, M. Porchia, F. Tisato and C. Marzano, *Chem. Rev*. 2014, **114**, 815–862.
[26] (a) A. Allam, L. Maigre, M. Alimi, R. Alves de Sousa, A. Hessani, E. Galardon, J.-M. Pagès and I. Artaud, *Bioconjugate Chem*. 2014, **25**, 1811–1819. (b) H. S. Nagendra Prasada, H. M. Manukumara, C. S. Karthika, L. Malleshab and P. Mallu, *Bioorg. Med. Chem*. 2019, **27**, 841–850.
[27] D. B. Adimpong, K. I. Sørensen, L. Thorsen, B. Stuer-Lauridsen, W. S. Abdelgadir, D. S. Nielsen, P. M. F. Derkx and L. Jespersen, *Appl. Environ. Microbiol.*, 2012, **78**, 7903–7914.
[28] G. Irmscher and G. Jung, *Eur. J. Biochem.*, 1977, **80**, 165–174.
[29] (a) Winter, G. *J. Appl. Cryst*. 2010, **43**, 186-190. (b) Bruker 2001, SADABS 2008/1. Bruker AXS Inc., Madison, Wisconsin, USA.
[30] (a) G.M. Sheldrick, *Acta Cryst*. 2015, **A71**, 3-8. (b) G.M. Sheldrick, *Acta Cryst*. 2015, **C71**, 3-8. (c) O. V. Dolomanov, L. J. Bourhis, R. J. Gildea, J. A. K. Howard and H. Puschmann, *J. Appl. Cryst*. 2009, **42**, 339-341.
[31] W. T. Eckenhoff and T. Pintauer, *Inorg. Chem*. 2007, **46**, 5844-5846.
[32] I. J. Bazley, E. A. Erie, G. M. Feiereisel, C. J. LeWarne, J. M. Peterson, K. L. Sandquist, K. D. Oshin and M. Zeller, *J. Chem. Educ*. 2018, **95**, 876−881.
[33] K. D. Karlin, J. C. Hayes, S. Juen, J. P. Hutchinson and J. Zubieta, *Inorg. Chem*. 1982, *21*, 4106-4108
[34] W. Xu, J. A. Craft, P. R. Fontenot, M. Barens, K. D. Knierim, J. H. Albering, F. A. Mautner and S. S. Massoud, *Inorg. Chim.* Acta, 2011, **373**, 159–166.
[35] S. C. N. Hsu, S. S. C. Chien, H. H. Z. Chen and M. Y. Chiang, *J. Chin. Chem. Soc*. 2007, **54**, 685-692.
[36] N. A. C. dos Santos, F. Lorandi, E. Badetti, K. Wurst, A. A. Isse, A. Gennaro, G. Licini and C. Zonta, *Polymer* 2017, **128**, 169-176.
[37] (a) E. Garribba and G. Micera, *J. Chem. Ed.*, 2006, *83*, 1229-1232. (b) N. Hall, M. Orio, M. Gennari, C. Wills, F. Molton, C. Philouze, G. B. Jameson, M. A. Halcrow, A. G. Blackman and C. Duboc, *Inorg. Chem*. 2016, **55**, 1497−1504.
